# Publisher Correction: Novel immunotherapeutics against LGR5 to target multiple cancer types

**DOI:** 10.1038/s44321-024-00139-6

**Published:** 2024-09-25

**Authors:** Hung-Chang Chen, Nico Mueller, Katherine Stott, Chrysa Kapeni, Eilidh Rivers, Carolin M Sauer, Flavio Beke, Stephen J Walsh, Nicola Ashman, Louise O’Brien, Amir Rafati Fard, Arman Ghodsinia, Changtai Li, Fadwa Joud, Olivier Giger, Inti Zlobec, Ioana Olan, Sarah J Aitken, Matthew Hoare, Richard Mair, Eva Serrao, James D Brenton, Alicia Garcia-Gimenez, Simon E Richardson, Brian Huntly, David R Spring, Mikkel-Ole Skjoedt, Karsten Skjødt, Marc de la Roche, Maike de la Roche

**Affiliations:** 1 University of Cambridge, Cancer Research UK Cambridge Institute, Robinson Way, Cambridge, CB2 0RE UK; 2 University of Cambridge, Department of Biochemistry, Tennis Court Road, Cambridge, CB2 1QW UK; 3 University of Cambridge, Yusuf Hamied Department of Chemistry, Lensfield Road, Cambridge, CB2 1EW UK; 4 University of Cambridge, Department of Pathology, Tennis Court Road, Cambridge, CB2 1QP UK; 5 Institute of Pathology, University of Bern, Murtenstrasse 31, CH-3008 Bern, Switzerland; 6 University of Cambridge, MRC Toxicology Unit, Tennis Court Road, Cambridge, CB2 1QR UK; 7 Department of Histopathology, Cambridge University Hospitals, NHS Foundation Trust, Main Drive, Cambridge, CB2 0QQ UK; 8 University of Cambridge, Department of Haematology, Puddicombe Way, Cambridge, CB2 0AW UK; 9 Rigshospitalet—University Hospital Copenhagen, Blegdamsvej 9, 2100 Copenhagen, Denmark; 10 Institute of Immunology and Microbiology, University of Copenhagen, Blegdamsvej 3B, 2200 Copenhagen, Denmark; 11 University of Southern Denmark Campusvej 55, Odense M, DK-5230 Denmark; 12 Present Address: Astra Zeneca, Cambridge, UK; 13 Present Address: MRC-University of Glasgow Centre for Virus Research, Glasgow, UK; 14 Present Address: Bicycle Therapeutics, Cambridge, UK; 15 Present Address: Charles River Laboratories, Saffron Walden, UK; 16 Present Address: Novo Nordisk, Måløv, Denmark

## Abstract

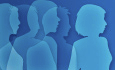

**Correction to:** *EMBO Mol Med* (2024) 16:2233–2261. 10.1038/s44321-024-00121-2 | Published online 21 August 2024

**The 12th author’s name is corrected**.

The 12th author’s name is corrected from: Arman Godsinia

To: (Changes in bold). Arman **Ghodsinia**

The original article has been corrected.

