# Supplementary material for: Novel immunotherapeutics against LGR5 to target multiple cancer types
Source: EMBO Mol Med. 2024 Aug 21;16(9):2233–61. doi: 10.1038/s44321-024-00121-2 (PMC11393416; doi:10.1038/s44321-024-00121-2)
Supplement: Supplementary file 1 — Appendix [file 44321_2024_121_MOESM1_ESM.pdf]

Appendix

Table of content:

Appendix Figure S1. LGR5 expression analysis in cancers. p2

Appendix Figure S2. Validation of α-LGR5v4. p8

Appendix Figure S3. LGR5 internalisation in NALM6 and LoVo cells. p9

Appendix Figure S4. Lack of adverse effects of ADC treatment on the small intestinal epithelia. p12

Appendix Figure S5. *In vitro* NALM6 targeting activity of purified CL-BiTE. p13

Appendix Figure S6. Specificity and *in vitro* efficacy of LGR5scFv-CAR-NK92 cells. p14

Appendix Figure S7. Antibody map of target epitopes on LGR5 for α-LGR5 and other reported antibodies. p15

Appendix Table S1. Binding affinities for therapeutic LGR5 antibodies. p16

Appendix Table S2. Antibodies and probes used in the study. p17

Appendix Table S3. Sequences of primers used in the study. p19

Appendix Table S4. Summary of statistical tests and p-values. P21

Supplemental references p25

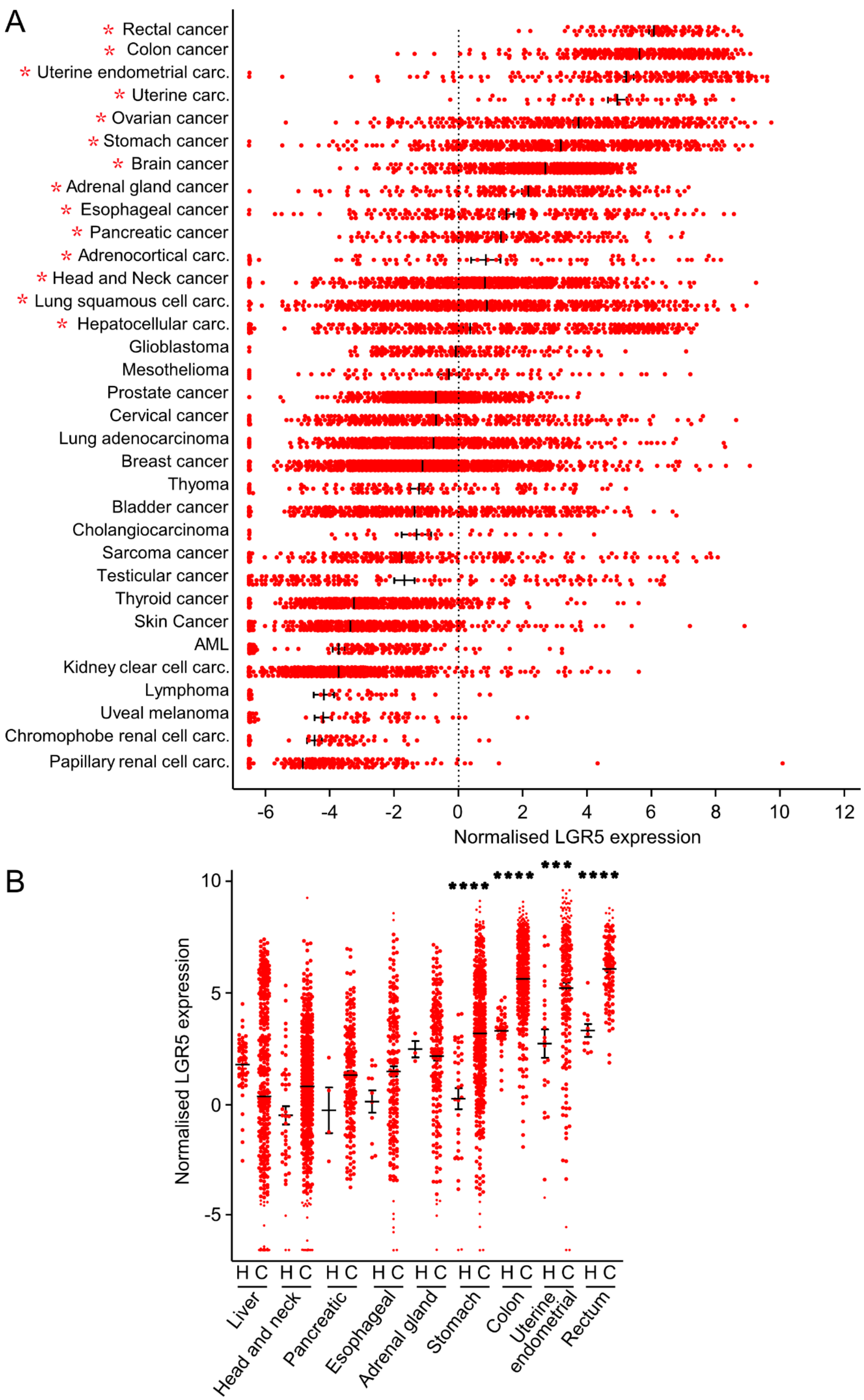
A
* Rectal cancer
* Colon cancer
* Uterine endometrial carc.
* Uterine carc.
* Ovarian cancer
* Stomach cancer
* Brain cancer
* Adrenal gland cancer
* Esophageal cancer
* Pancreatic cancer
* Adrenocortical carc.
* Head and Neck cancer
* Lung squamous cell carc.
* Hepatocellular carc.
Glioblastoma
Mesothelioma
Prostate cancer
Cervical cancer
Lung adenocarcinoma
Breast cancer
Thyoma
Bladder cancer
Cholangiocarcinoma
Sarcoma cancer
Testicular cancer
Thyroid cancer
Skin Cancer
AML
Kidney clear cell carc.
Lymphoma
Uveal melanoma
Chromophobe renal cell carc.
Papillary renal cell carc.
-6 -4 -2 0 2 4 6 8 10 12
Normalised LGR5 expression
B
Normalised LGR5 expression
10
5
0
-5
**** **** *** ****
H C H C H C H C H C H C H C H C H C
Liver
Head and neck
Pancreatic
Esophageal
Adrenal gland
Stomach
Colon
Uterine endometrial
Rectum

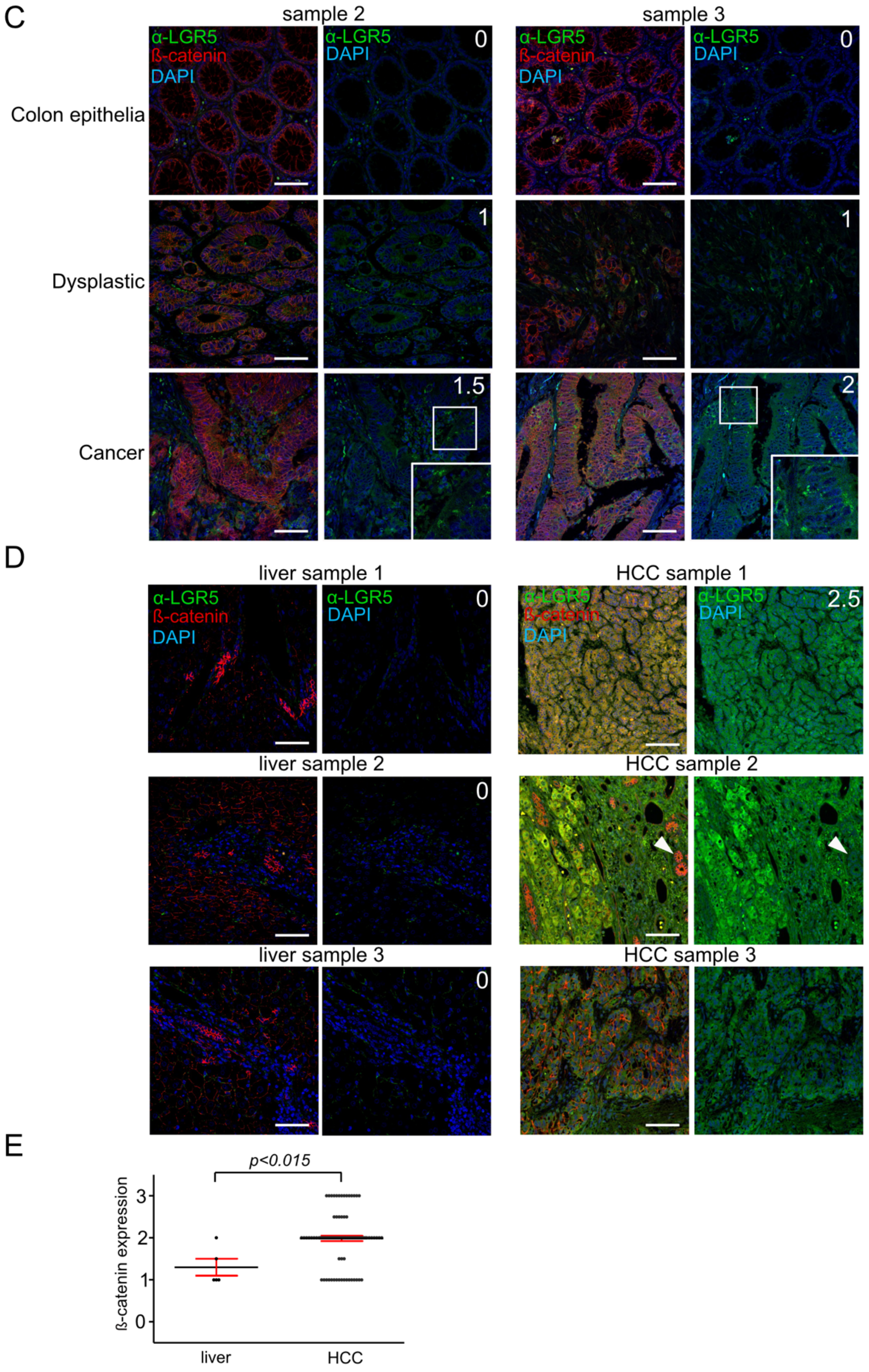
C
sample 2
sample 3
α-LGR5
ß-catenin
DAPI
Colon epithelia
Dysplastic
Cancer
0
1
1.5
2
D
liver sample 1
liver sample 2
liver sample 3
HCC sample 1
HCC sample 2
HCC sample 3
2.5
E
p<0.015
ß-catenin expression
liver
HCC

F

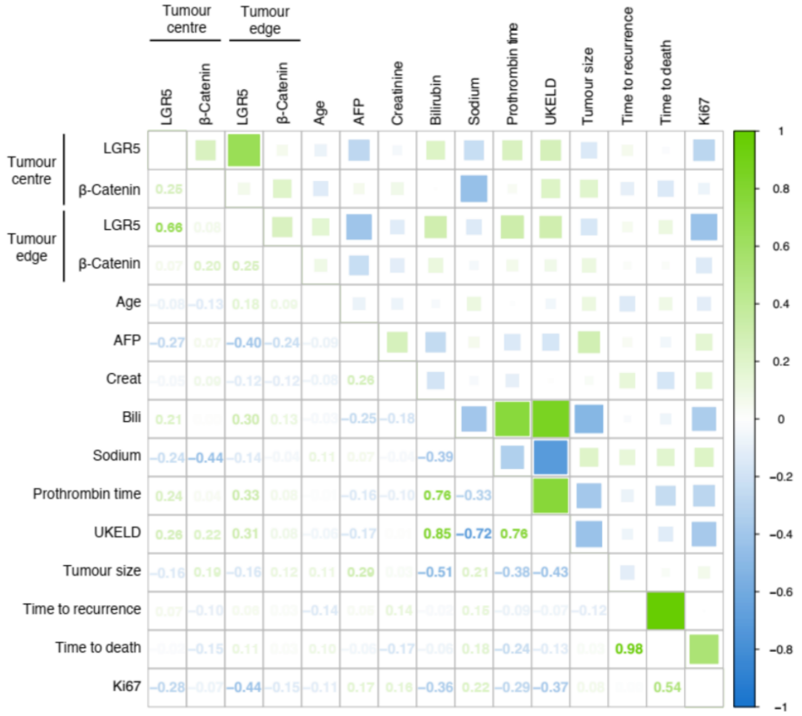

Tumour centre
Tumour edge
LGR5
β-Catenin
Age
AFP
Creatinine
Bilirubin
Sodium
Prothrombin time
UKELD
Tumour size
Time to recurrence
Time to death
Ki67
Creat
Bili

G

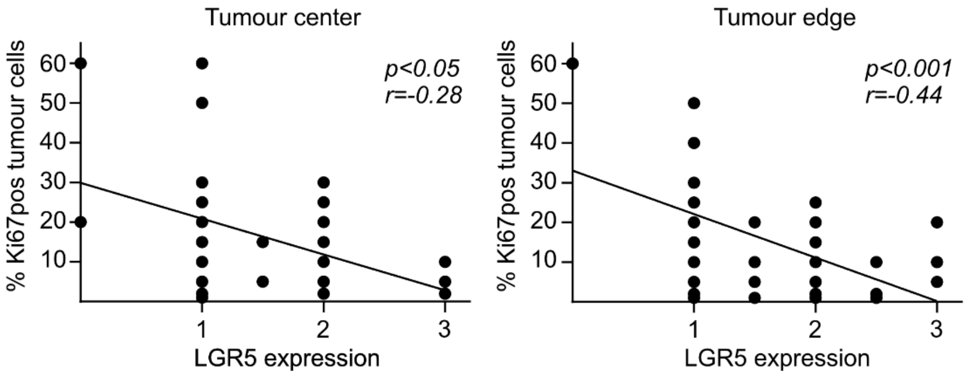

Tumour center
Tumour edge
% Ki67pos tumour cells
LGR5 expression
p<0.05
r=-0.28
p<0.001
r=-0.44

H

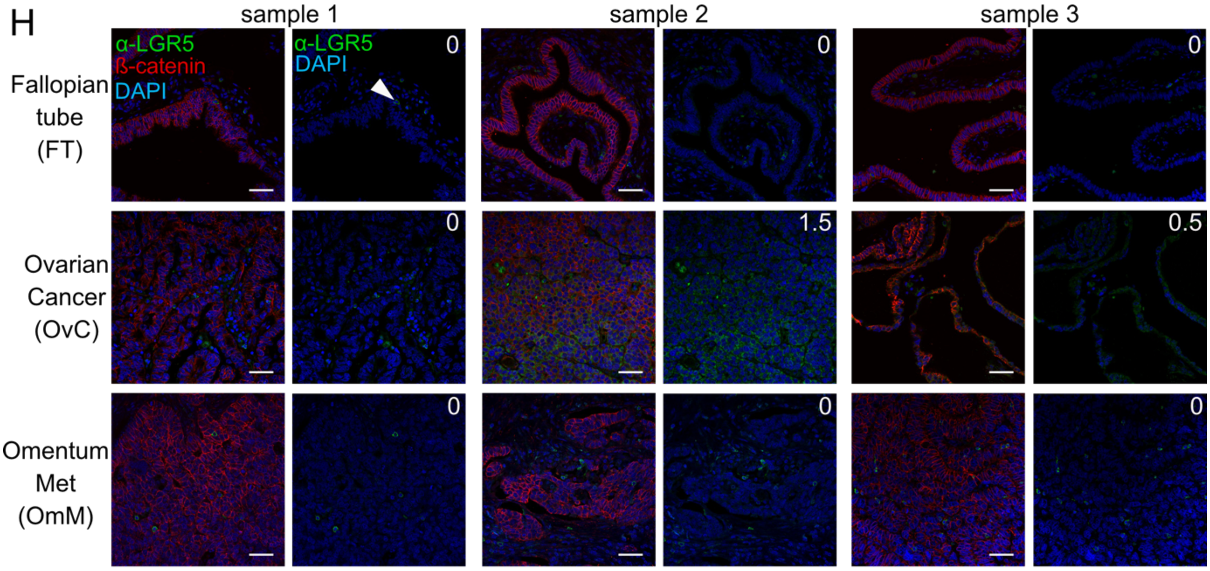
sample 1
sample 2
sample 3
Fallopian tube (FT)
Ovarian Cancer (OvC)
Omentum Met (OmM)
α-LGR5
ß-catenin
DAPI
α-LGR5
DAPI
0
0
0
0
1.5
0.5
0
0
0

I

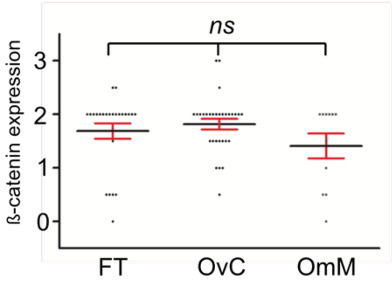
ns
ß-catenin expression
3
2
1
0
FT
OvC
OmM

J

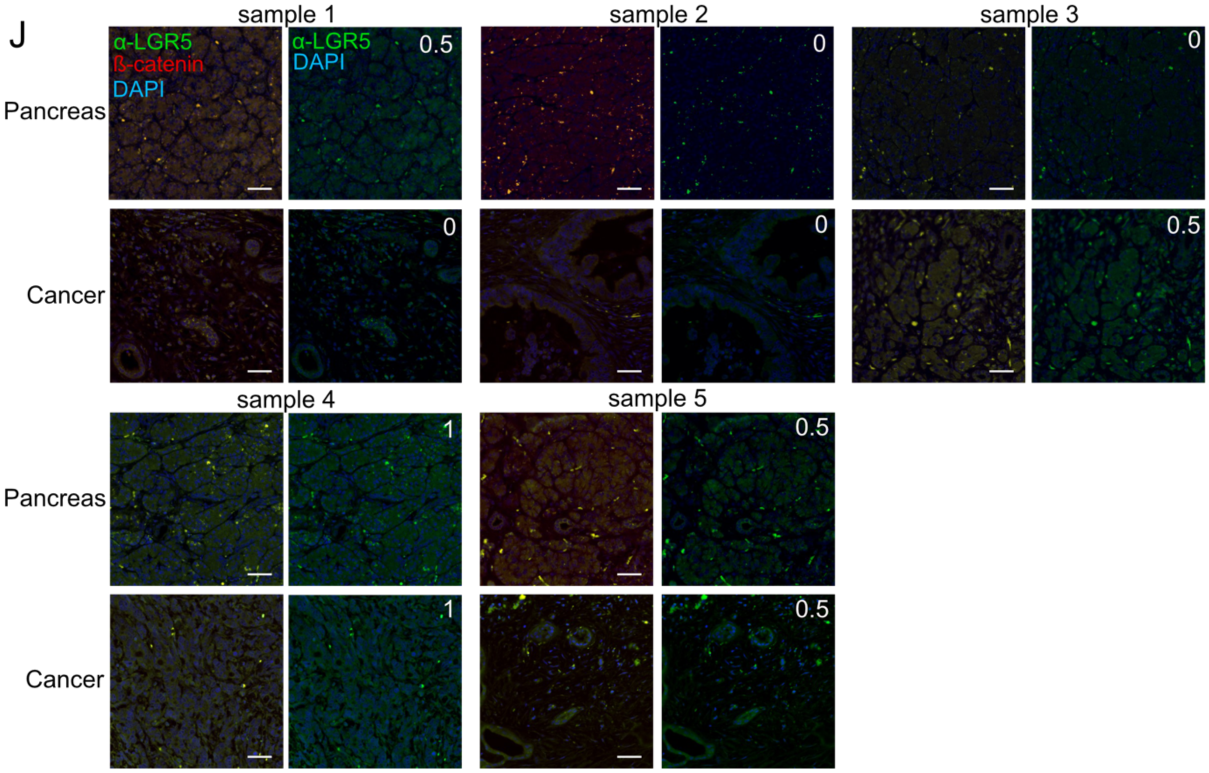
sample 1
sample 2
sample 3
Pancreas
Cancer
α-LGR5
ß-catenin
DAPI
α-LGR5
DAPI
0.5
0
0
0
0
0.5
sample 4
sample 5
Pancreas
Cancer
1
0.5
1
0.5

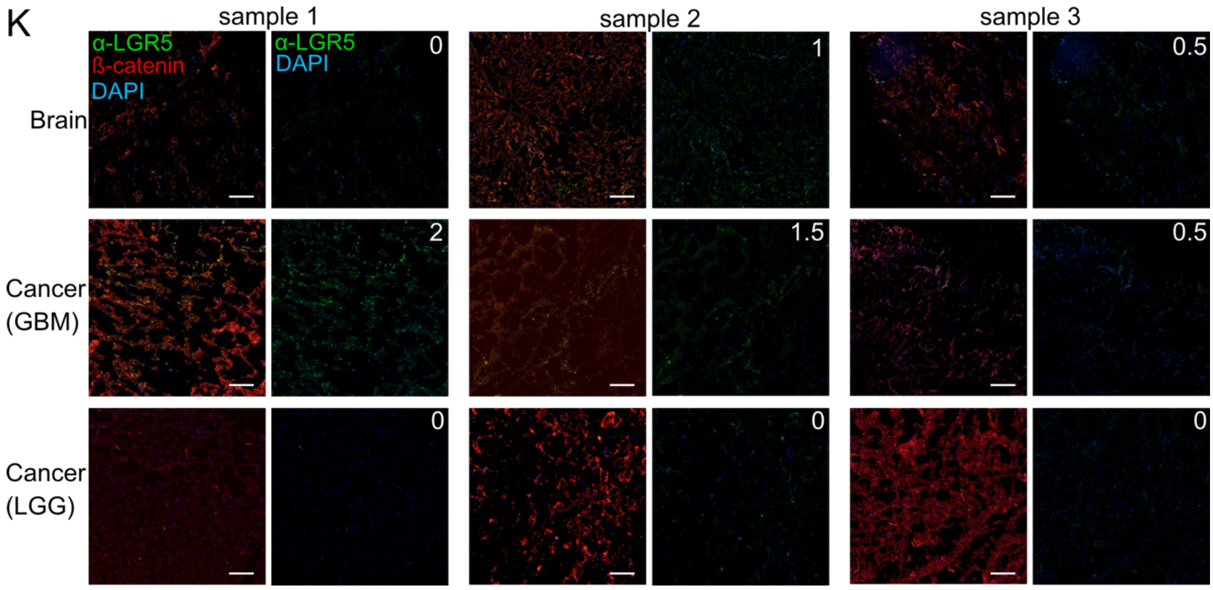

Appendix Figure S1. LGR5 expression analysis in cancers.

A. Normalised (log2 median-centred) LGR5 gene expression levels for cancer subtypes, ordered by median LGR5 gene expression. Read counts were quantile normalized across the genome for direct comparison amongst cancer subtypes and sample sets and median expression levels for extracted LGR5 data determined across the entire dataset. The dotted line indicates the median LGR5 expression across all pan-cancer tumours. Tumour subtypes for which more than 70% of samples had higher than median LGR5 expression (dashed line) were defined as "high LGR5 tumours" are denoted with a red *.
B. Comparison of LGR5 gene expression between healthy tissue (H) and cancer (C) for selected high LGR5 tumours. Significance difference in LGR5 expression between cancer and healthy tissue (Wilcoxon test) are indicated: **, *p<0.01*, ***, *p<0.001*, **** *p<0.0001*.
C. LGR5 and β-catenin protein levels in sections from two CRC tumour resections with regions of normal colon epithelia (*top panels*), dysplastic tissue (*middle panels*) and cancer (*lower panels*). White numbers are the relative values for LGR5 protein expression using the scoring system applied to all tissue and cancer biopsies. Scale bars, 10 μM.
D. Representative images from 3 liver samples (*left panels*) and 3 of the 95 HCC cases from the Cambridge HCC TMA (*right panels*). White numbers are the score for LGR5 expression levels. Scale bars, 10 μM.
E. Quantitation of β-catenin expression levels in 8 liver resections (liver) and the HCC cases from the Cambridge HCC TMA. Level of significant difference, *p-value*, between the sample sets was determined by two-tailed t-test.
F. Correlation matrix between LGR5 or β-catenin protein expression levels and phenotypic metrics determined for the biopsies comprising the Cambridge HCC TMA. AFP, levels of the tumour serum biomarker alpha-fetoprotein at the time of transplant; UKELD, UK Model for End-Stage Liver Disease score (Barber *et al* 2011), Ki67, quantification of Ki67 immunohistochemistry antigen used as an index of proliferation. Correlation values were computed pairwise using complete observations (i.e., removing missing values) using the Spearman Rank method (values shown left of the diagonal). Level of correlation amongst phenotypic metrics is scaled from highly positive - dark green colour and large squares, to highly negative - dark blue and large squares (shown to the right of the diagonal).
G. Plot of Ki67 levels versus LGR5 protein expression in the tumour centre (left graph) or the tumour edge (right edge) for HCC cases with clinical and molecular features of the non-proliferative HCC sub-class. Significance for inverse correlation of LGR5 expression levels and

percent cells expressing Ki67 determined by Spearman correlation test and represented by p-value (*p*) and correlation coefficient (*r*).

H. Representative images for fallopian tube tissue (FT), ovarian cancers (OvC;) and omentum metastasis (OmM). Arrowhead shown on the first fallopian tube sample indicate a very rare instance of epithelial cells containing LGR5 positive intracellular puncta. White numbers, scored values for relative LGR5 protein expression. Scale bars, 40 μM.

I. Relative expression levels of β-catenin in the fallopian tube, ovarian cancer and omentum cancer sample sets. There was no significance in β-catenin protein levels amongst fallopian tube (FT), OvC or OmM samples, determined using two-tailed t-test.

J. Images of β-catenin and LGR5 expression in the five matched pancreas samples and pancreatic cancer cases (Cancer). White numbers, scored values for relative LGR5 protein expression. Low level expression of the two proteins was apparent for all samples. Scale bars, 40 μM.

K. Representative images for brain tissue (*left panel set*), GBM (*middle panel set*) and LGG (*right panel set*). White numbers, scored values for relative LGR5 expression. Scale bars, 40 μM.

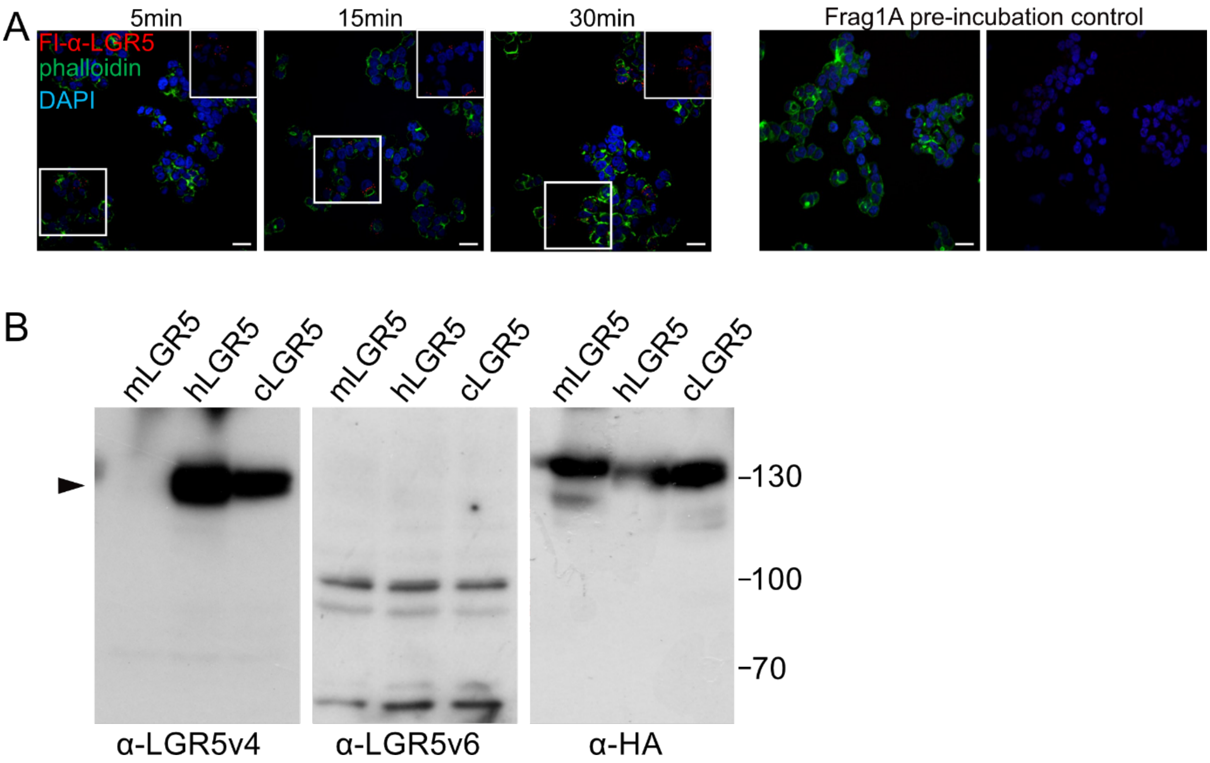

Appendix Figure S2. Validation of α-LGR5v4.

A. Time course of Fl-α-LGR5 (red) internalisation by LoVo cells. F-actin and nuclei were visualised by Alexa488-Phalloidin and Hoechst probes, respectively. For the two panels on the right, Fl-α-LGR5 was pre-incubated with Frag1A. In the rightmost image, the signal from Alexa488 phalloidin has been omitted. n=2 independent experiments. Scale bars, 10 μM.

B. Western blot analysis of LGR5 protein levels in lysates from HEK293T cell overexpressing, *lanes 1-3*, mLGR5-eGFP, hLGR5-eGFP and cLGR5-eGFP using humanised α-LGR5v4, α-LGR5v6 and α-HA. Arrow denotes approximate migration distance of eGFP fusions.

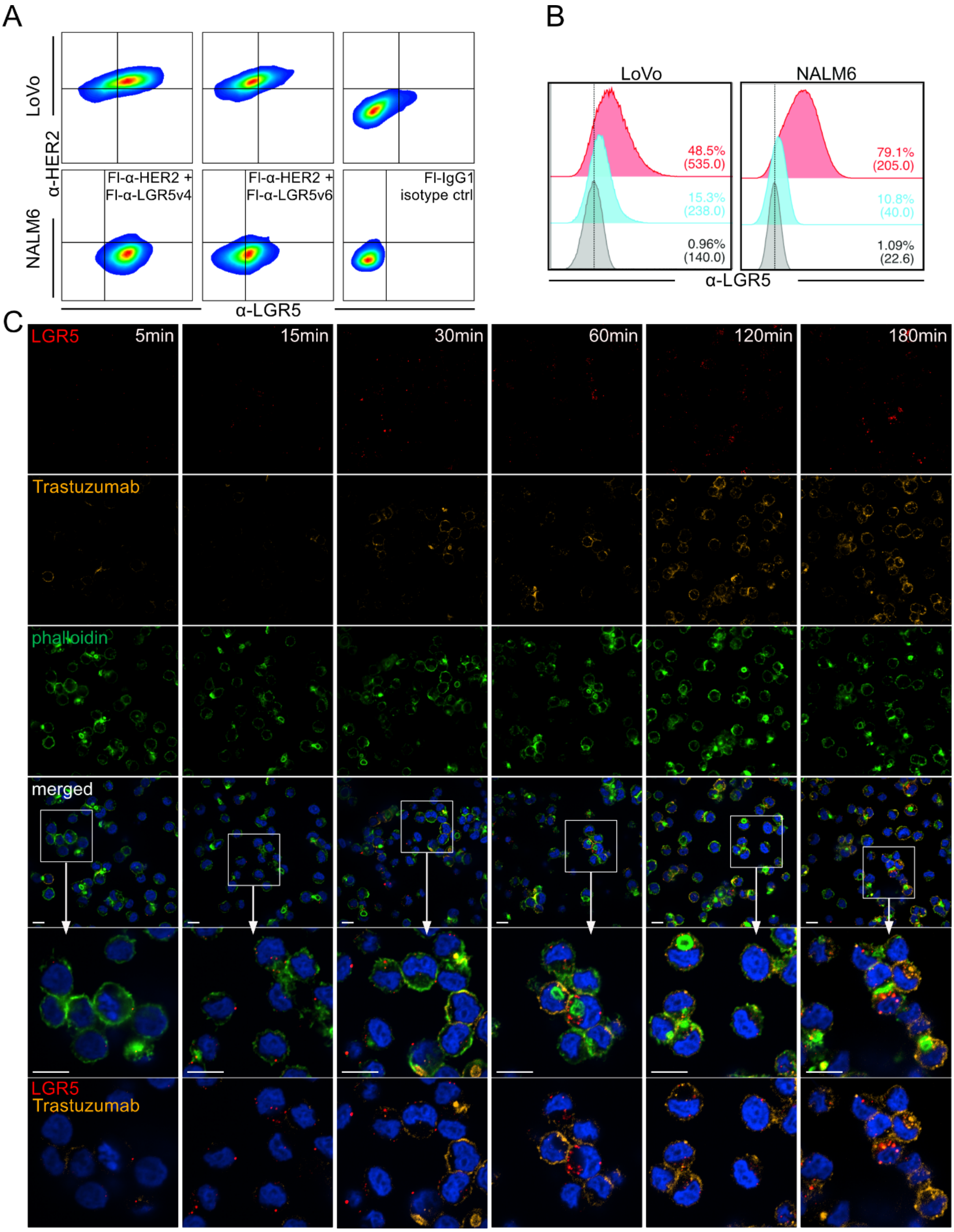
A
LoVo
α-HER2
NALM6
Fl-α-HER2 +
Fl-α-LGR5v4
Fl-α-HER2 +
Fl-α-LGR5v6
Fl-IgG1
isotype ctrl
α-LGR5
B
LoVo
NALM6
48.5%
(535.0)
15.3%
(238.0)
0.96%
(140.0)
79.1%
(205.0)
10.8%
(40.0)
1.09%
(22.6)
α-LGR5
C
LGR5
5min
15min
30min
60min
120min
180min
Trastuzumab
phalloidin
merged
LGR5
Trastuzumab

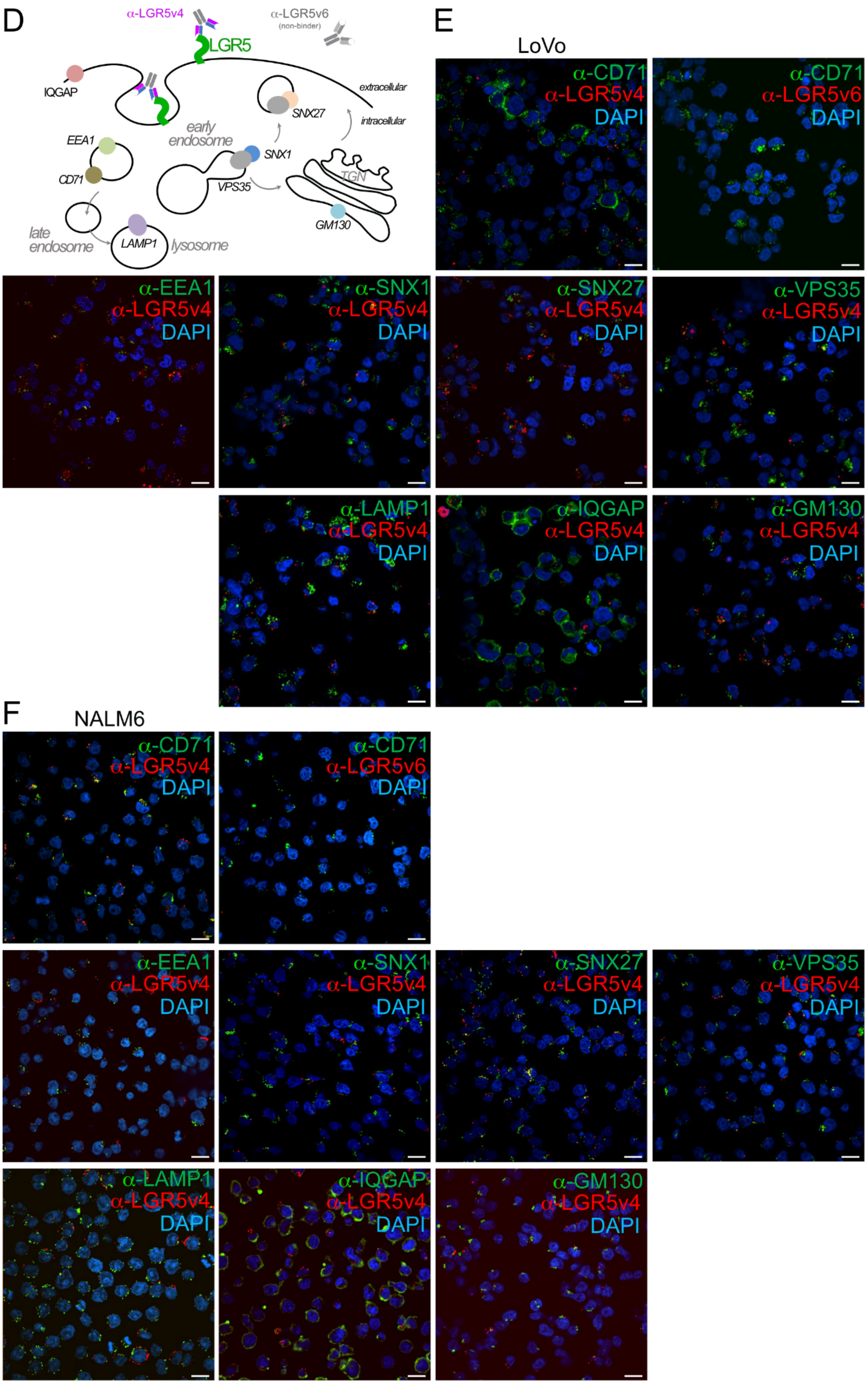
D
α-LGR5v4
α-LGR5v6
(non-binder)
LGR5
IQGAP
extracellular
intracellular
SNX27
early endosome
EEA1
SNX1
CD71
VPS35
TGN
GM130
late endosome
LAMP1
lysosome
E
LoVo
α-CD71
α-LGR5v4
DAPI
α-CD71
α-LGR5v6
DAPI
α-EEA1
α-LGR5v4
DAPI
α-SNX1
α-LGR5v4
DAPI
α-SNX27
α-LGR5v4
DAPI
α-VPS35
α-LGR5v4
DAPI
α-LAMP1
α-LGR5v4
DAPI
α-IQGAP
α-LGR5v4
DAPI
α-GM130
α-LGR5v4
DAPI
F
NALM6
α-CD71
α-LGR5v4
DAPI
α-CD71
α-LGR5v6
DAPI
α-EEA1
α-LGR5v4
DAPI
α-SNX1
α-LGR5v4
DAPI
α-SNX27
α-LGR5v4
DAPI
α-VPS35
α-LGR5v4
DAPI
α-LAMP1
α-LGR5v4
DAPI
α-IQGAP
α-LGR5v4
DAPI
α-GM130
α-LGR5v4
DAPI

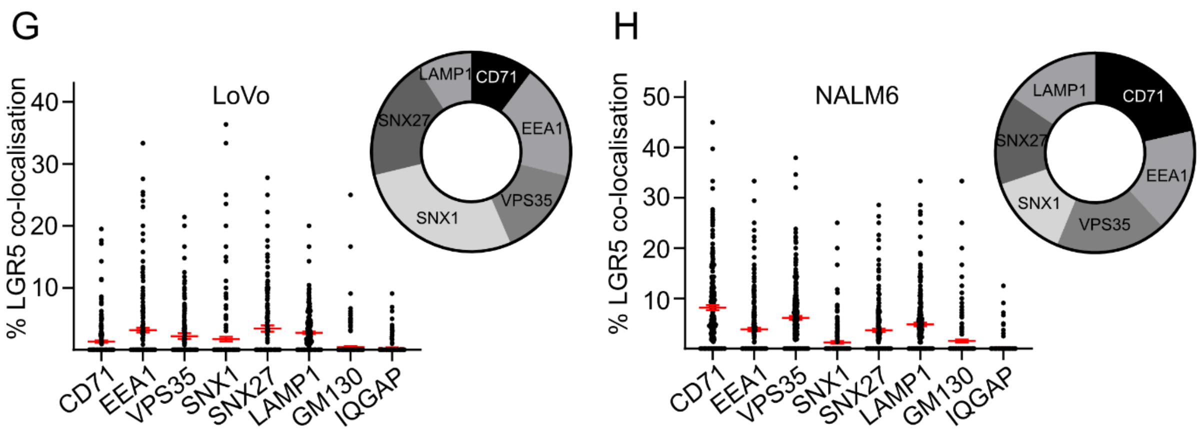

Appendix Figure S3. LGR5 internalisation in NALM6 and LoVo cells.

A. Flow cytometric analysis of LoVo and NALM6 cells after 60 minutes co-incubation with Fl-α-LGR5v4 and Fl-α-HER2 (*left panels*), Fl-α-LGR5v6 and Fl-α-HER2 (middle panels) or Fl-IgG1 isotype controls, n=one experiment.
B. Flow cytometric analyses of LoVo and NALM6 cells after 60 minutes incubation with Fl-α-LGR5v4 (red) or Fl-α-LGR5v4 pre-incubated with Frag1A (cyan) or Fl-IgG1 isotype control (grey), n=3 independent experiments.
C. Representative fields of view for time course of Fl-α-LGR5v4 and Fl-α-HER2 association and internalisation by NALM6 cells. Scored association and internalisation data is summarised in **Fig. 4D and E**. Scale bars, 20 μM.
D. Graphical representation of the intracellular markers used for immunofluorescence detection and co-localisation with internalised Fl-α-LGR5v4.
E. Representative single plane images showing immune detection of the intracellular markers (green) in LoVo cells that have been incubated with Fl-α-LGR5v4 (red) or Fl-α-LGR5v6 (second panel only) for 30 minutes. Scale bars, 10 μM. n=3 independent experiments.
F. Representative single plane images showing immune detection of the intracellular markers (green) in NALM6 cells that have been incubated with Fl-α-LGR5v4 (red) or Fl-α-LGR5v6 (second panel only) for 30 minutes. Scale bars, 10 μM. n=3 independent experiments.
G. Analysis of co-localisation between internalized Fl-α-LGR5v4 puncta and markers of various intracellular compartments and IQGAP1 in LoVo cells after a 60-minute incubation. Representative images shown above in **Suppl. Fig. 4F**. *Left* - Co-localisation data were derived from automatic scoring of puncta in images of internalised Fl-α-LGR5v4 and intracellular markers detected by indirect immunofluorescence. *Right* - wheel graph indicating fractional association of internalised Fl-α-LGR5v4 with specific intracellular vesicle markers in LoVo cells. The level of co-localisation between Fl-α-LGR5v4 and the either GM130 (2.9%) or IQGAP (2.1%) did not significantly deviate from the null hypothesis (no interaction) and are excluded from these analyses. Error bars indicate standard deviation, data is the composite of a minimum of 200 cells over two independent experiments.
H. Co-localisation between internalized FL-α-LGR5 puncta and markers of various intracellular compartments and IQGAP1 in NALM6 cells after a 60-minute incubation, scoring as above. Representative images used for co-localisation datasets are shown above in **Suppl. Fig. 4G**. *Right* - wheel graph showing fractional association of internalised Fl-α-LGR5v4 with specific intracellular vesicle markers in NALM6 cells. The level of co-localisation between Fl-α-LGR5v4 and the either GM130 (2.9%) or IQGAP (2.1%) did not significantly deviate from the null hypothesis (no interaction) and are excluded from these analyses. Data is derived from two independent experiments.

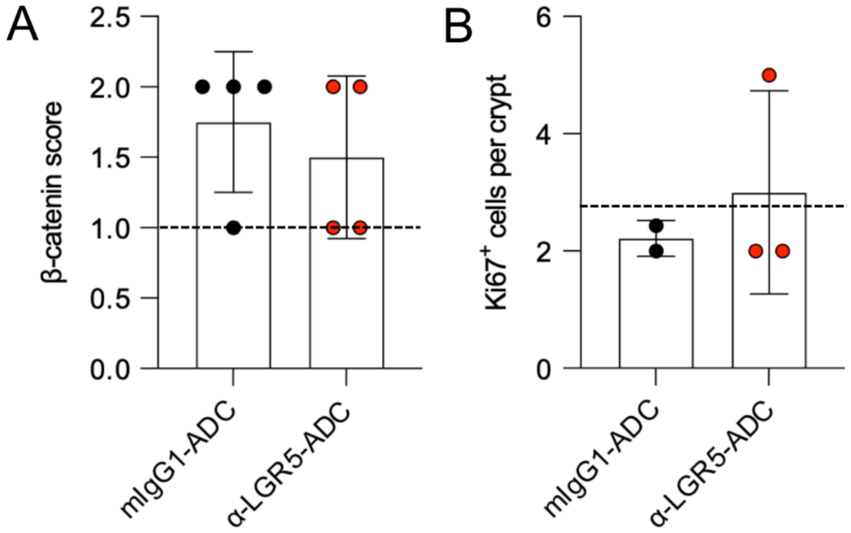

Appendix Figure S4. Lack of adverse effects of ADC treatment on the small intestinal epithelia.

A. Relative β-catenin score based on fluorescent staining intensity relative to intensity of DAPI nuclear stain.
B. Quantification of number of Ki67+ cells per crypt in a single section of small intestinal epithelia of ADC treated mice and untreated controls. There were no significant differences for the β-catenin score or Ki67 enumeration between the treatment groups and untreated controls. n=2-4 mice per condition from experiment shown in **Figure 5**.

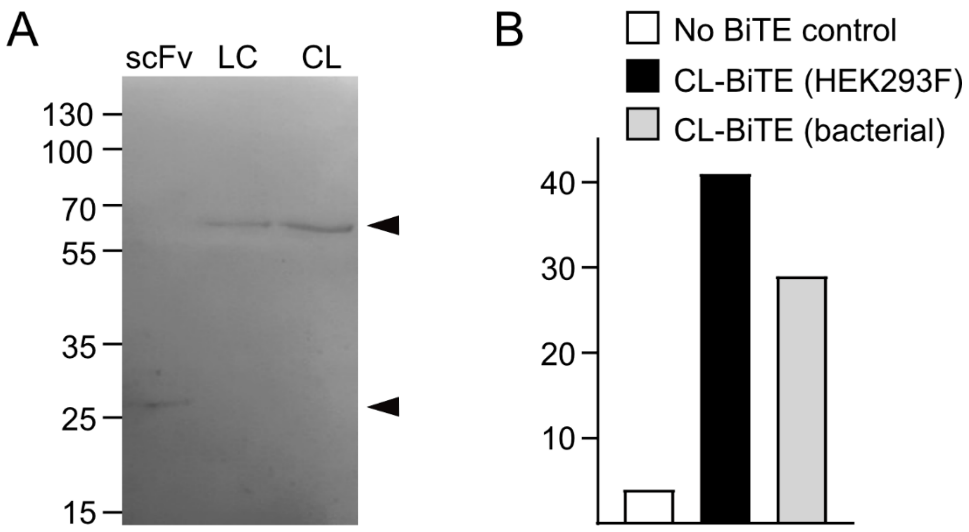

Appendix Figure S5. *In vitro* NALM6 targeting activity of purified CL-BiTE.

A. SDS-PAGE gel of CL-BiTE and LC-BiTE produced from HEK293F cell expression.
B. Comparative NALM6 target cell killing by cytotoxic $CD8^+$ T cells stimulated by the addition of CL BiTE. Control values are cell killing by cytotoxic $CD8^+$ T cells in the absence of CL-BiTE. Values are a single replicate killing assay.

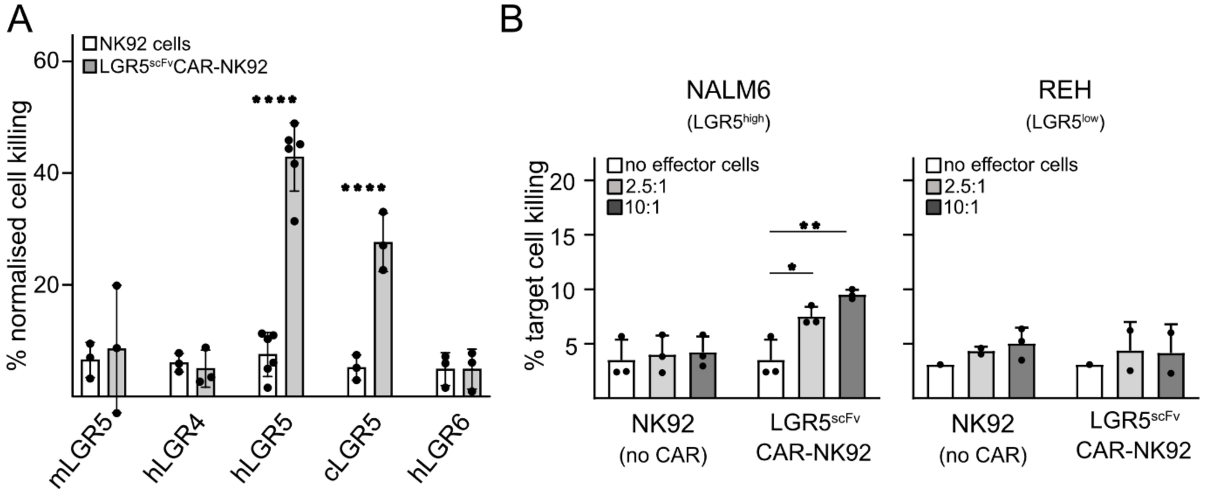

Appendix Figure S6. Specificity and *in vitro* efficacy of LGR5^{scFv}CAR-NK92 cells.

- **A.** HEK293T target cells were transfected with eGFP-fused LGR5 family transgenes and incubated with either effector NK92 cells (parental line) or NK92 cells stably transduced with LGR5^{scFv}CAR at an effector to target ratio of 10:1 for 9 hours. Specific killing of HEK293T target cells is shown. Error bars represent SD of n=3-6 independent experiments. Significant differences in target cell killing between parental NK92 and LGR5^{scFv}CAR -NK92 cells was determined by 2-way ANOVA, ****, $p<0.0001$
- **B.** Killing of NALM6 (left) and REH (right) target cells by parental NK92 cells or LGR5^{scFv}CAR-NK92 cells at effector to target ratios of 2.5:1 and 10:1 after 12 hours was assessed. Error bars represent SD of n=3 independent experiments. Significant differences in target cell killing were determined by two-tailed t-test. *, $p<0.05$ and **, $p<0.01$.

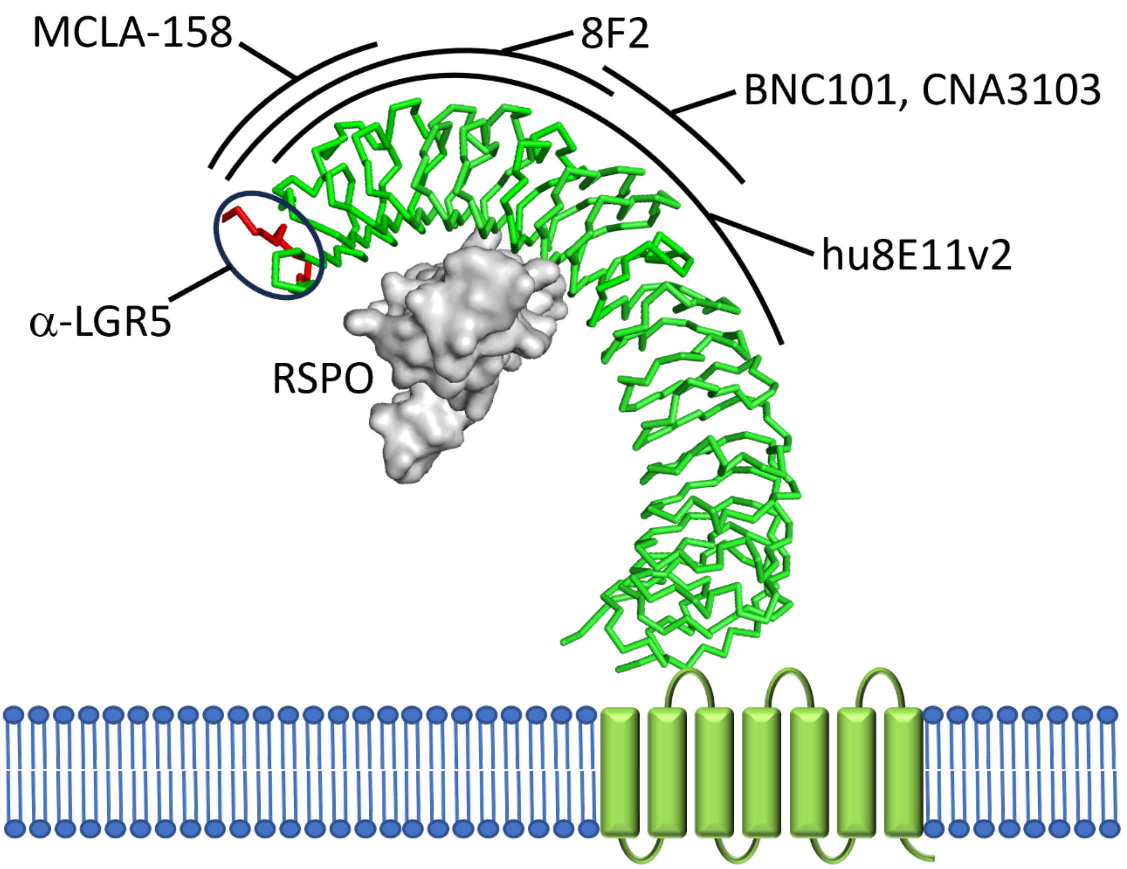

Appendix Figure S7. Antibody map of target epitopes on LGR5 for α-LGR5 and other reported antibodies.

Structure of the LGR5 extracellular domain (stick structure representation in green and red) bound to R-spondin (surface structure representation in gray) derived from PDB structure 1JE6. The circled red coloured N-terminus marks the location of the α-LGR5 target epitope. The MCLA-158, 8F2 and hu8E11vs epitopes overlap with that of α-LGR5, however these binding interfaces also require amino acids within LRRs1-9. BNC101 LGR5 binding site is located within LRR2 6-9. The binding sites for α-LGR5 and MCLA-158 do not overlap with regions of the extracellular domain that interact with R-spondin ligands that bind to the concave surface contained within LRRs 4-9. The 8F2, BNC101 and hu8E11v2 antibodies bind to the convex surface of LGR5, and as with α-LGR5 and MCLA-158, do not interfere with R-spondin signalling.

Appendix Table S1. Binding affinities for therapeutic LGR5 antibodies.

Name of antibody	Reference	*Kd* (nM)	Apparent *Kd* (nM)*	Therapeutic modality	Clinical stage
α-LGR5 (murine)	This study	1.1		ADC	Pre-clinical
α-LGR5v4	This study	2.0		ADC	Pre-clinical
α-LGR5^{scFv}	This study	0.77		CAR and BiTE	Pre-clinical
MCLA-158	(51)		0.86	Bispecific, fused to α-EGFR^{scFv}	Phase I
BNC101**	(52)	16		ADCC	Phase I
CNA3103	(53)	***			Phase I/IIa
8F2	(27)		6	ADC	Pre-clinical
hu8E11v2	(26)		0.2****	ADC	Pre-clinical

*Apparent *Kd* values determined using measurements of antibody binding to cells overexpressing LGR5.

**The Phase I clinical trial for BNC101 (NCT02726334) has been terminated by the sponsor and the antibody been repurposed as an scFv fragment in the CAR-T modality as CNA3103.

*** No binding data available for the scFv fragment.

**** The humanised hu8E11v2 antibody was not tested whereas the parental murine antibody has an apparent *Kd* value of 0.2 nM.

Appendix Table S2. Antibodies and probes used in the study.

Antibodies/probes	Clone	Conjugate	Application	Dilution	Manufacturer
Primary antibodies					
Human α-vinculin	4650	none	WB	1:5000	Cell Signaling Technology (4650T)
Human α-β-actin	AC-15	none	WB	1:5000	Sigma-Aldrich (A5441)
Human α-HER2	Trastuzumab	none	IF and ADC	1:50	Roche
Human α-CD71	OKT9	none	IF	1:100	Thermo Fisher Scientific (14-0719-82)
Human α-SNX1	51/SNX1	none	IF	1:25	BD Biosciences (611482)
Human α-SNX27	1C6	none	IF	1:100	Abcam (ab77799)
Human α-EEA1	14/EEA1	none	IF	1:100	BD Biosciences (610457)
Human α-VPS35	Goat polyclonal	none	IF	1:100	Novus biologicals (NB100-1397)
Human α-IQGAP1	D6E3J	none	IF	1:50	Cell Signaling Technology (29016)
Human α-LAMP1	H4A3	none	IF	1:100	Developmental Studies Hybridoma Bank
Human α-GM130	35/GM130	none	IF	1:500	BD Biosciences (610822)
Human α-CC3 (Asp175)	5A1E	none	IF	1:500	Cell Signaling Technology (9664)
Human α-HA	12CA5	none	WB	1:2000	a kind gift from Sean Munroe
Human α-B220	RA3-6B2	BV785	FACS	1:200	Biolegend (103246)
Human α-CD3	UCHT1	BUV395	FACS	1:300	BD Biosciences (563546)
Human α-CD3	UCHT1	BV510	FACS	1:300	Biolegend (300448)
Human α-CD4	RPA-T4	BV605	FACS	1:300	Biolegend (300556)
Human α-CD8	RPA-T8	BV711	FACS	1:300/1:100	Biolegend (301044)
Human α-CD8	RPA-T8	BV785	FACS	1:100	Biolegend (301046)
Human α-CD19	HIB19	BV421	FACS	1:100	Biolegend (302234)
Human α-CD19	SJ25C1	BUV395	FACS	1:100	BD Biosciences (563551)
Human α-CD34	581	PE	FACS	1:100	Thermo Fisher Scientific (CD34-581-04)
Human α-CD45	HI30	BUV737	FACS	1:300/1:50	BD Biosciences (568524)
Human α-CD45	HI30	FITC	FACS	1:50	Biolegend (982316)

Human α-CD95	DX2	BV421	FACS	1:100	Biolegend (305624)
Human α-CD127	A019D5	BV711	FACS	1:50	Biolegend (351327)
Human α-CCR7	G043H7	PE-Cy7	FACS	1:100	Biolegend (353226)
Human α-HLA-DR	L243	BV510	FACS	1:50	Biolegend (307646)
Human α-IgM	MHM-88	PE-Cy7	FACS	1:50	Biolegend (314532)
Human CD45RA	HI100	PE-Cy7	FACS	1:100	Biolegend (304126)
Mouse α-CD45.1	A20	FITC	FACS	1:100	Biolegend (110706)
Mouse α-CD45	30-F11	BV605	FACS	1:100	Biolegend (103140)
Human α-CD25	M-A251	BV421	FACS	1:100	BD Biosciences (562443)
Human α-CD69	FN50	APC	FACS	1:100	Biolegend (310910)
α-FLAG	M2	Agarose beads	WB	0.5 ml	Sigma-Aldrich (M8823)
Secondary antibodies					
Goat-α-mouse IgG		HRP	WB	1:15000	Dako (P044701-2)
Goat-α-rabbit IgG		HRP	WB	1:15000	Dako (P044801-2)
Donkey-α-mouse IgG (H+L) Highly Cross-Absorbed		AF488	IF	1:400	Thermo Fisher Scientific (A10037)
Goat-α-rabbit IgG (H+L) Highly Cross-Absorbed		AF488	IF	1:400	Thermo Fisher Scientific (A11034)
Donkey-α-goat IgG (H+L) Highly Cross-Absorbed		AF488	IF	1:400	Thermo Fisher Scientific (A32814)
AffiniPure Donkey-α-human IgG (H+L)		AF647	IF	1:400	Jackson ImmunoResearch (709-005-149)
Probes					
Phalloidin		AF488	IF	1:100	Thermo Fisher Scientific (A12379)
Phalloidin		AF568	IF	1:100	Thermo Fisher Scientific (A12380)
Phalloidin		AF647	IF	1:100	Thermo Fisher Scientific (A30107)
Hoechst 33342		n/a	IF	1:5000	Thermo Fisher Scientific (H3570)

Appendix Table S3. Sequences of primers used in the study.

primer name	primer sequence (5'- 3')
Generation of CAR construct	
Forward_LGR5scFv	GACAGACTGAGTCGCCCGGGACGCGTCCCACCATGCCGCTGC
Reverse_LGR5scFv	GCGTCGTGGTGCTGCTCACGGTCACGG
Forward_CAR	CGTGAGCAGCACCACGACGCCAGCGC
Reverse_CAR	CACCATGGTGGCGACCGGTGGATCCCGAGGGGGCAGGGC
RAD display expression for epitope mapping	
Forward_SEQ1	GGCGGCGGGCTTAAGGGCAGCTCTCCCAGGTCTG
Reverse_SEQ1	CCGCCTCCCTTAAGCAACATCCTGCCGTCGGGC
Forward_SEQ1A	GGCGGCGGGCTTAAGGGCAGCTCTCCCAGGTCTG
Reverse_SEQ1A	CCGCCTCCCTTAAGATGACAGTGTGTGGGGCAGCC
Forward_SEQ1B	GGCGGCGGGCTTAAGAGGGGCTGCCCCACACACTG
Reverse_SEQ1B	CCGCCTCCCTTAAGCAACATCCTGCCGTCGGGCTC
Forward_SEQ2	GGCGGCGGGCTTAAGAGTATGAACAACATCAGTCAGCTG
Reverse_SEQ2	CCGCCTCCCTTAAGTGTCAGAGCGTTTCCCGCAAG
Forward_SEQ3	GGCGGCGGGCTTAAGGAGTTACGTCTTGCGGGAAAC
Reverse_SEQ3	CCGCCTCCCTTAAGATTCTGCAGCATAAGAACTTTAAGAC
Forward_SEQ4	GGCGGCGGGCTTAAGTACAGTCTTAAAGTTCTTATGCTGCAG
Reverse_SEQ4	CCGCCTCCCTTAAGCAGGGATTGAAGGCTTCGCAAATT
Generation of BiTE constructs	
CL_common Forward	CAGTCCAGCTTGAAGTTGGCGGTGGTGGATCGGGCGGTGGTGGATCGGCTGAGATC
CL_Reverse pcDNA	AGGGCCCTCTAGATGCATGCCTAGTGATGGTGATGGTGATGGCTGCTCACGGTCACGG
CL_Reverse pETDuet-1	AAGCATTATGCGAATTCTCAGTGGTGATGGTGGTGATGGCTGCTC
LC_Forward pcDNA	GACTCACTATAGGGAGACCCAGAATTCGCCGCCATGCCGCT<br>GCTGCTACTGCTGCCCCTGCTGTGGGCAGGGGCGCTAGCTG<br>AGATCGTGATGACTCAAAG

LC_Forward pETDuet-1	AAGGAGATATACGAATTCATGGCTGAGATCGTGATGACTCAAAG
LC_common Reverse	GATTGTTGCAGCTTGATGTCCGATCCACCACCGCCGCTGCTCACGGTCACGGTGGTG
Generation of murine, human and cyno LGR4/5/6 GFP fusion constructs	
Forward_mLgr4	GCCGTCGACGGTACCGCGCCACCTCTCTGCGCTG
Reverse_mLgr4	GTCCCCGGGCGGATCCGTCTCTGACTCTCGGTAGA
Forward_mLgr5	GACGGTACCGGCAGCTCACCGGGACCAGAT
Reverse_mLgr5	GTCCCCGGGCGGATCCGAGACATGGGACAAATGCA
Forward_hLGR4	CATGGTACCGCGCCGCCTCTCTG
Reverse_hLGR4	CGCACCGGTGTCTTTAACTCTTGGTAGATTG
Forward_hLGR6	GACGGTACCGCCCCCCAGCCCGGC
Reverse_hLGR6	CCCACCGGTCACGTGTGAAGCAAAGGCC
Forward_sub1 cynoLGR5	CCAGGTCTGGTGCGCTGCTGCGGGGCTGCCCCA
Reverse_sub1 cynoLGR5	TGGGGCAGCCCCGCAGCAGCGCACCAGACCTGG
Forward_sub2 cynoLGR5	TCGACGGTACCAGCAGCTCGCCCAGGTCTGG
Reverse_sub2 cynoLGR5	CCAGACCTGGGCGAGCTGCTGGTACCGTCGA
Generation of LGR5 construct for immunization	
Forward	GCGTGGATCCCCGGAATTCAGCTCTCCCAGGTCTGGTGTG
Reverse	GTCACGATGCGGCCGCTCGAGTTAATTCTGCAGCATAAGAACTTT

Appendix Table S4. Summary of statistical tests and p-values.

Figure	Group	Statistical analysis	p-value
Fig 2B	Colon epithelia vs cancer stage I	Kruskal-Wallis test with Dunn's multiple comparisons test	<0.0001
Fig 2B	Colon epithelia vs cancer stage II	Kruskal-Wallis test with Dunn's multiple comparisons test	0.0002
Fig 2B	Colon epithelia vs cancer stage III	Kruskal-Wallis test with Dunn's multiple comparisons test	<0.0001
Fig 2B	Colon epithelia vs cancer stage IV	Kruskal-Wallis test with Dunn's multiple comparisons test	0.0003
Fig 2D	Liver vs HCC	Mann-Whitney test	<0.0001
Fig 2E	Fallopian tube vs OvC	Kruskal-Wallis test with Dunn's multiple comparisons test	0.2395
Fig 2E	Fallopian tube vs OmM	Kruskal-Wallis test with Dunn's multiple comparisons test	>0.9999
Fig 2F	Brain vs LGG	Kruskal-Wallis test with Dunn's multiple comparisons test	0.1033
Fig 2F	Brain vs GBM	Kruskal-Wallis test with Dunn's multiple comparisons test	0.4551
Fig 2G	Healthy B cells vs B-ALL cell lines	Mann-Whitney test	0.0004
Fig 2G	Healthy B cells vs ALL PDXs	Mann-Whitney test	<0.0001
Fig 2G	Healthy B cells vs ALL primary tumor	Mann-Whitney test	<0.0001
**Figure**	**Group**	**Statistical analysis**	**p-value**
Fig 4D	Fl-a-HER2 vs Fl-a-LGR5v4 – 5 min	2way ANOVA with Šidák's multiple comparisons test	0.0053
Fig 4D	Fl-a-HER2 vs Fl-a-LGR5v4 – 15 min	2way ANOVA with Šidák's multiple comparisons test	0.0008
Fig 4D	Fl-a-HER2 vs Fl-a-LGR5v4 – 30 min	2way ANOVA with Šidák's multiple comparisons test	0.5764
Fig 4D	Fl-a-HER2 vs Fl-a-LGR5v4 – 60 min	2way ANOVA with Šidák's multiple comparisons test	0.1586
Fig 4D	Fl-a-HER2 vs Fl-a-LGR5v4 – 180 min	2way ANOVA with Šidák's multiple comparisons test	0.4515
Fig 4E	Fl-a-HER2 vs Fl-a-LGR5v4 – 5 min	2way ANOVA with Šidák's multiple comparisons test	<0.0001
Fig 4E	Fl-a-HER2 vs Fl-a-LGR5v4 – 15 min	2way ANOVA with Šidák's multiple comparisons test	<0.0001
Fig 4E	Fl-a-HER2 vs Fl-a-LGR5v4 – 30 min	2way ANOVA with Šidák's multiple comparisons test	<0.0001
Fig 4E	Fl-a-HER2 vs Fl-a-LGR5v4 – 60 min	2way ANOVA with Šidák's multiple comparisons test	<0.0001
Fig 4E	Fl-a-HER2 vs Fl-a-LGR5v4 – 180 min	2way ANOVA with Šidák's multiple comparisons test	<0.0001
**Figure**	**Group**	**Statistical analysis**	**p-value**
Fig 5C	IgG1-ADC vs a-LGR5-ADC	2way ANOVA	<0.0001
Fig 5E	Spleen mass (IgG1-ADC vs a-LGR5-ADC)	Unpaired t-test	0.0033

Fig 5E	Spleen NALM6 cell count (IgG1-ADC vs a-LGR5-ADC)	Unpaired t-test	0.0418
Fig 5E	Blood NALM6 cell count (IgG1-ADC vs a-LGR5-ADC)	Unpaired t-test	0.0078
Fig 5E	Bone NALM6 cell count (IgG1-ADC vs a-LGR5-ADC)	Unpaired t-test	0.0167
Fig 5F	a-LGR5v4-ADC vs a-LGR5v6-ADC	2way ANOVA	0.0343
Fig 5H	Spleen mass (a-LGR5v4-ADC vs a-LGR5v6-ADC)	Unpaired t-test	0.0002
Fig 5H	Spleen NALM6 cell count (a-LGR5v4-ADC vs a-LGR5v6-ADC)	Unpaired t-test	<0.0001
Fig 5H	Blood NALM6 cell count (a-LGR5v4-ADC vs a-LGR5v6-ADC)	Unpaired t-test	0.0077
Fig 5H	Bone NALM6 cell count (a-LGR5v4-ADC vs a-LGR5v6-ADC)	Unpaired t-test	0.7655
**Figure**	**Group**	**Statistical analysis**	**p-value**
Fig 6A	PBMC + PBMC & NALM6 (ctrl)	2way ANOVA with Šidák's multiple comparisons test	0.9192
Fig 6A	PBMC + PBMC & NALM6 (scFv)	2way ANOVA with Šidák's multiple comparisons test	0.7294
Fig 6A	PBMC + PBMC & NALM6 (LC)	2way ANOVA with Šidák's multiple comparisons test	0.0013
Fig 6A	PBMC + PBMC & NALM6 (CL)	2way ANOVA with Šidák's multiple comparisons test	<0.0001
Fig 6B	PBMC + PBMC & NALM6 (ctrl)	2way ANOVA with Šidák's multiple comparisons test	0.8622
Fig 6B	PBMC + PBMC & NALM6 (scFv)	2way ANOVA with Šidák's multiple comparisons test	0.6745
Fig 6B	PBMC + PBMC & NALM6 (LC)	2way ANOVA with Šidák's multiple comparisons test	0.1045
Fig 6B	PBMC + PBMC & NALM6 (CL)	2way ANOVA with Šidák's multiple comparisons test	<0.0001
Fig 6C	scFv vs LC (5:1)	2way ANOVA with Dunnett's multiple comparisons test	0.0643
Fig 6C	scFv vs CL (5:1)	2way ANOVA with Dunnett's multiple comparisons test	<0.0001
Fig 6C	scFv vs LC (10:1)	2way ANOVA with Dunnett's multiple comparisons test	0.0262
Fig 6C	scFv vs CL (10:1)	2way ANOVA with Dunnett's multiple comparisons test	<0.0001
Fig 6D	CD8 vs CD8+CL-BiTE	2way ANOVA	0.0028
Fig 6E	CD8 vs CD8+CL-BiTE	Unpaired t-test	0.0319
**Figure**	**Group**	**Statistical analysis**	**p-value**
Fig 7A	T cell vs LGR5^{scFv}CAR T (mLGR5)	2way ANOVA with Šidák's multiple comparisons test	>0.9999
Fig 7A	T cell vs LGR5^{scFv}CAR T (hLGR4)	2way ANOVA with Šidák's multiple comparisons test	<0.9997
Fig 7A	T cell vs LGR5^{scFv}CAR T (hLGR5)	2way ANOVA with Šidák's multiple comparisons test	<0.0001
Fig 7A	T cell vs LGR5^{scFv}CAR T (cLGR5)	2way ANOVA with Šidák's multiple comparisons test	<0.0001

Fig 7A	T cell vs LGR5^{scFv}CAR T (hLGR6)	2way ANOVA with Šidák's multiple comparisons test	<0.9998
Fig 7B	NALM6 vs T cell	2way ANOVA with Dunnett's multiple comparisons test	0.0026
Fig 7B	NALM6 vs LGR5^{scFv}CAR T	2way ANOVA with Dunnett's multiple comparisons test	<0.0001
Fig 7B	HepG2 vs T cell	2way ANOVA with Dunnett's multiple comparisons test	0.0197
Fig 7B	HepG2 vs LGR5^{scFv}CAR T	2way ANOVA with Dunnett's multiple comparisons test	<0.0001
Fig 7B	LoVo vs T cell	2way ANOVA with Dunnett's multiple comparisons test	0.0906
Fig 7B	LoVo vs LGR5^{scFv}CAR T	2way ANOVA with Dunnett's multiple comparisons test	<0.0001
Fig 7C	D3 (PBS vs T cell)	2way ANOVA with Dunnett's multiple comparisons test	0.9994
Fig 7C	D3 (PBS vs LGR5^{scFv}CAR T)	2way ANOVA with Dunnett's multiple comparisons test	0.7624
Fig 7C	D4 (PBS vs T cell)	2way ANOVA with Dunnett's multiple comparisons test	0.3657
Fig 7C	D4 (PBS vs LGR5^{scFv}CAR T)	2way ANOVA with Dunnett's multiple comparisons test	0.7882
Fig 7C	D7 (PBS vs T cell)	2way ANOVA with Dunnett's multiple comparisons test	0.2211
Fig 7C	D7 (PBS vs LGR5^{scFv}CAR T)	2way ANOVA with Dunnett's multiple comparisons test	0.6470
Fig 7C	D10 (PBS vs T cell)	2way ANOVA with Dunnett's multiple comparisons test	0.6504
Fig 7C	D10 (PBS vs LGR5^{scFv}CAR T)	2way ANOVA with Dunnett's multiple comparisons test	0.0155
Fig 7C	D11 (PBS vs T cell)	2way ANOVA with Dunnett's multiple comparisons test	0.5867
Fig 7C	D11 (PBS vs LGR5^{scFv}CAR T)	2way ANOVA with Dunnett's multiple comparisons test	0.0388
Fig 7E	PBS vs T cell	1way ANOVA with Tukey's multiple comparisons test	0.2395
Fig 7E	PBS vs LGR5^{scFv}CAR T	1way ANOVA with Tukey's multiple comparisons test	0.0118
Fig 7E	T cell vs LGR5^{scFv}CAR T	1way ANOVA with Tukey's multiple comparisons test	0.0010
**Figure**	**Group**	**Statistical analysis**	**p-value**
Fig EV1G	eGFP expressing, - R-spondin, IgG1 vs a-LGR5	2way ANOVA with Šidák's multiple comparisons test	0.9999
Fig EV1G	eGFP expressing, + R-spondin, IgG1 vs a-LGR5	2way ANOVA with Šidák's multiple comparisons test	0.0580
Fig EV1G	LGR5-eGFP expressing, - R-spondin, IgG1 vs a-LGR5	2way ANOVA with Šidák's multiple comparisons test	0.8487
Fig EV1G	LGR5-eGFP expressing, + R-spondin, IgG1 vs a-LGR5	2way ANOVA with Šidák's multiple comparisons test	0.3067
**Figure**	**Group**	**Statistical analysis**	**p-value**
Fig EV3A	697 (scFv vs BiTE) 5:1	2way ANOVA with Šidák's multiple comparisons test	0.0908
Fig EV3A	697 (scFv vs BiTE) 10:1	2way ANOVA with Šidák's multiple comparisons test	0.4626

Fig EV3A	NALM6 (scFv vs BiTE) 5:1	2way ANOVA with Šidák's multiple comparisons test	0.0006
Fig EV3A	NALM6 (scFv vs BiTE) 10:1	2way ANOVA with Šidák's multiple comparisons test	<0.0001
Fig EV3B	LoVo (scFv vs BiTE) 5:1	2way ANOVA with Šidák's multiple comparisons test	0.2111
Fig EV3B	LoVo (scFv vs BiTE) 10:1	2way ANOVA with Šidák's multiple comparisons test	0.0025
Fig EV3B	SW480 (scFv vs BiTE) 5:1	2way ANOVA with Šidák's multiple comparisons test	0.9922
Fig EV3B	SW480 (scFv vs BiTE) 10:1	2way ANOVA with Šidák's multiple comparisons test	0.9836
Fig EV3C	scFv vs CL BiTE (LC2-9h)	2way ANOVA with Šidák's multiple comparisons test	0.2526
Fig EV3C	scFv vs CL BiTE (CRH-9h)	2way ANOVA with Šidák's multiple comparisons test	0.0009
**Figure**	**Group**	**Statistical analysis**	**p-value**
Fig EV4A	697 vs NALM (E:T 0.625:1)	2way ANOVA with Šidák's multiple comparisons test	0.9443
Fig EV4A	697 vs NALM (E:T 1.25:1)	2way ANOVA with Šidák's multiple comparisons test	0.4912
Fig EV4A	697 vs NALM (E:T 2.5:1)	2way ANOVA with Šidák's multiple comparisons test	0.0536
Fig EV4A	697 vs NALM (E:T 5:1)	2way ANOVA with Šidák's multiple comparisons test	0.0166
Fig EV4A	697 vs NALM (E:T 10:1)	2way ANOVA with Šidák's multiple comparisons test	0.0013
Fig EV4B	T cell vs LGR5^{scFv}CAR T (LoVo)	2way ANOVA with Tukey's multiple comparisons test	<0.0001
Fig EV4B	T cell vs LGR5^{scFv}CAR T (SW480)	2way ANOVA with Tukey's multiple comparisons test	<0.0001
Fig EV4C	T cell vs LGR5^{scFv}CAR T (LC2-5h)	2way ANOVA with Šidák's multiple comparisons test	0.6358
Fig EV4C	T cell vs LGR5^{scFv}CAR T (CRH-5h)	2way ANOVA with Šidák's multiple comparisons test	0.0385
Fig EV4C	T cell vs LGR5^{scFv}CAR T (LC2-9h)	2way ANOVA with Šidák's multiple comparisons test	0.6909
Fig EV4C	T cell vs LGR5^{scFv}CAR T (CRH-9h)	2way ANOVA with Šidák's multiple comparisons test	0.0023

Supplemental reference

Barber K, Madden S, Allen J, Collett D, Neuberger J and Gimson A. Elective liver transplant list mortality: Development of a United Kingdom end-stage liver disease score. *Transplantation* 2011 92 469–76.
