# Supplementary material for: Novel immunotherapeutics against LGR5 to target multiple cancer types
Source: EMBO Mol Med. 2024 Aug 21;16(9):2233–61. doi: 10.1038/s44321-024-00121-2 (PMC11393416; doi:10.1038/s44321-024-00121-2)
Supplement: Supplementary file 3 — Source data Fig. 1 [file 44321_2024_121_MOESM3_ESM.zip › Figure 1/Figure 1B/Figure1B_Domain_mapping_Western_Blot.pptx]

## Slide 1
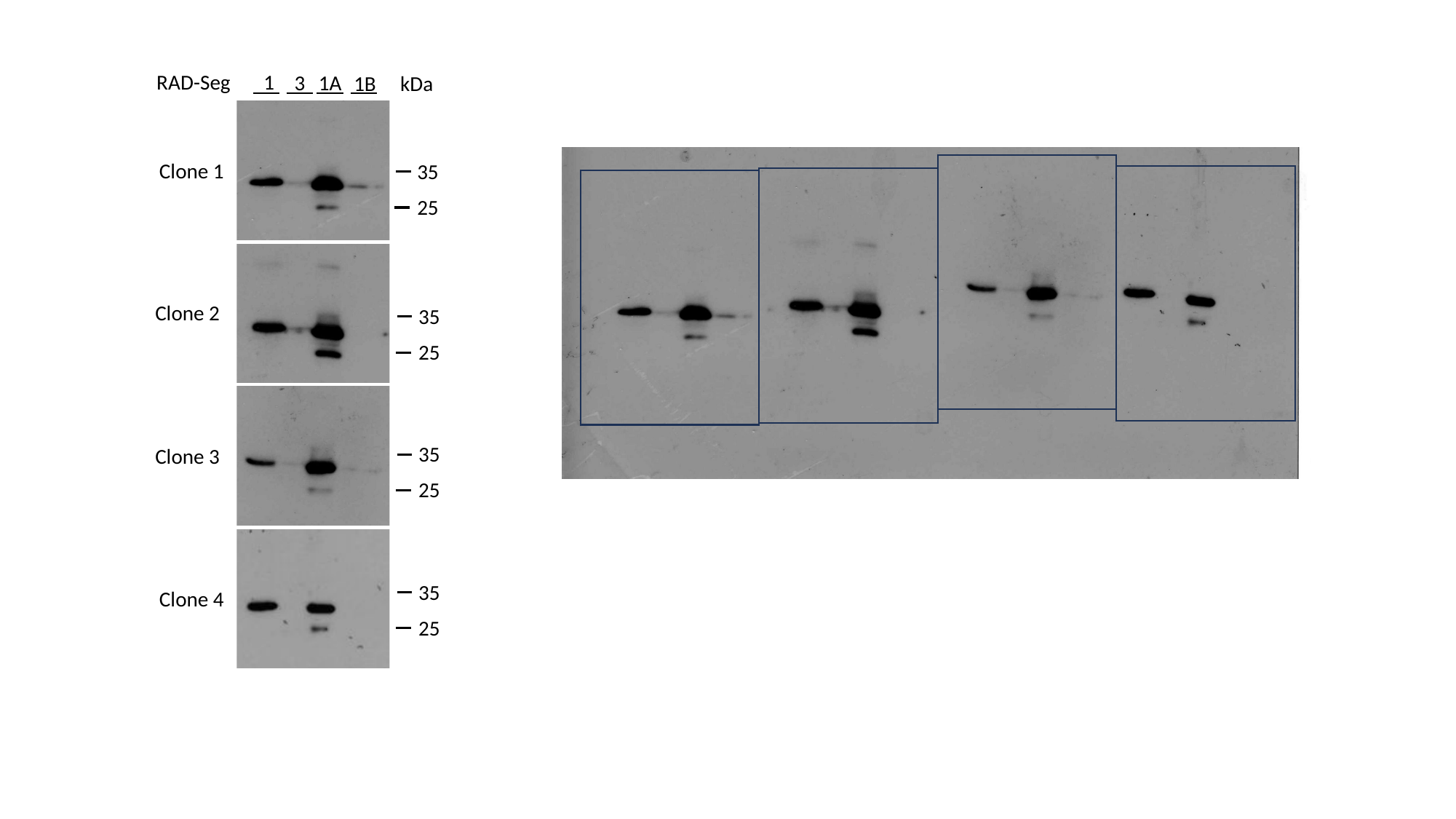

RAD-Seg
1
3
1A
kDa
1B
Clone 1
35
25
Clone 2
35
25
35
25
Clone 3
35
25
Clone 4

## Slide 2
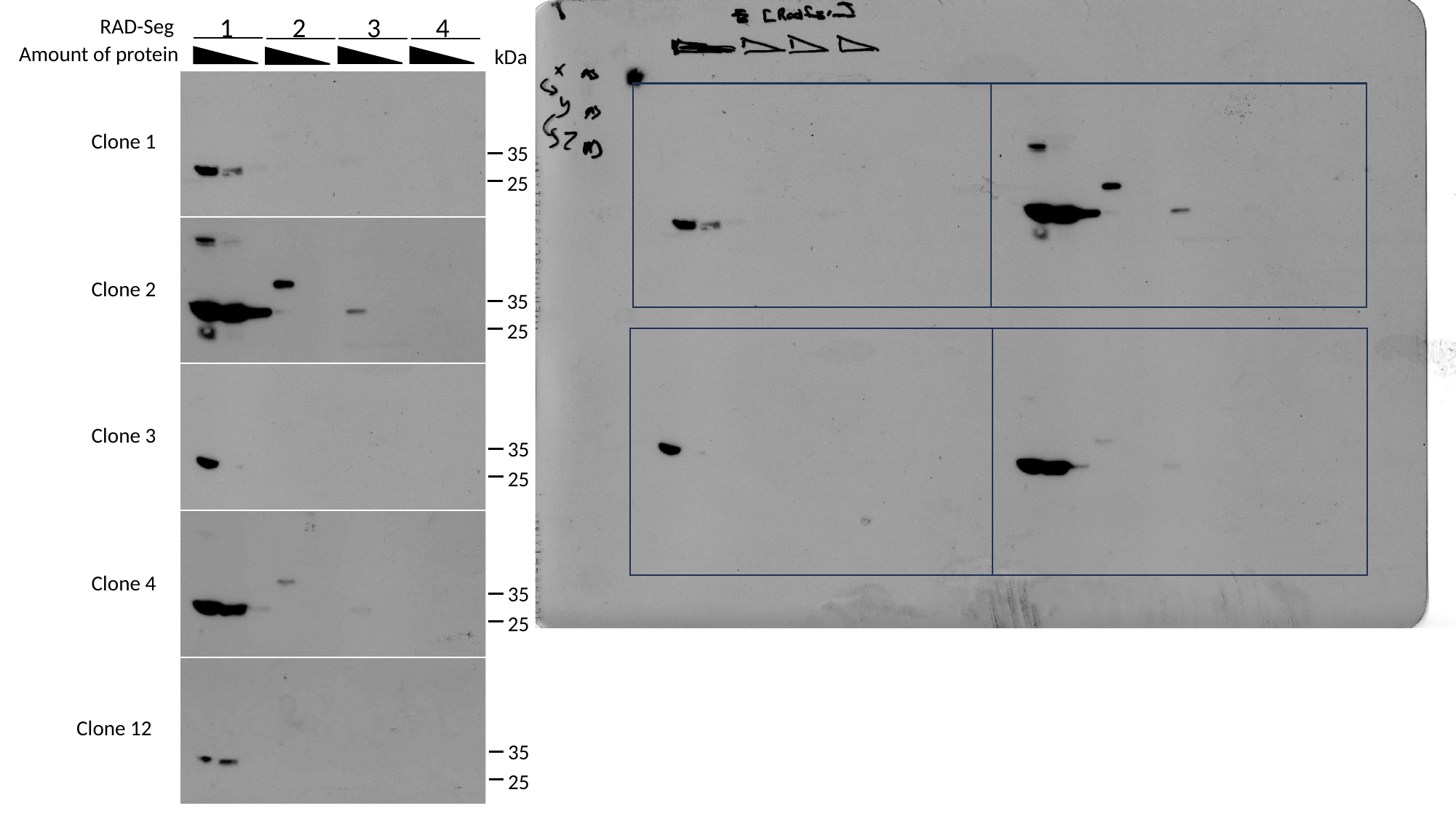

2
1
3
4
RAD-Seg
Amount of protein
Clone 1
Clone 2
Clone 3
Clone 4
Clone 12
kDa
35
25
35
25
35
25
35
25
35
25

## Slide 3
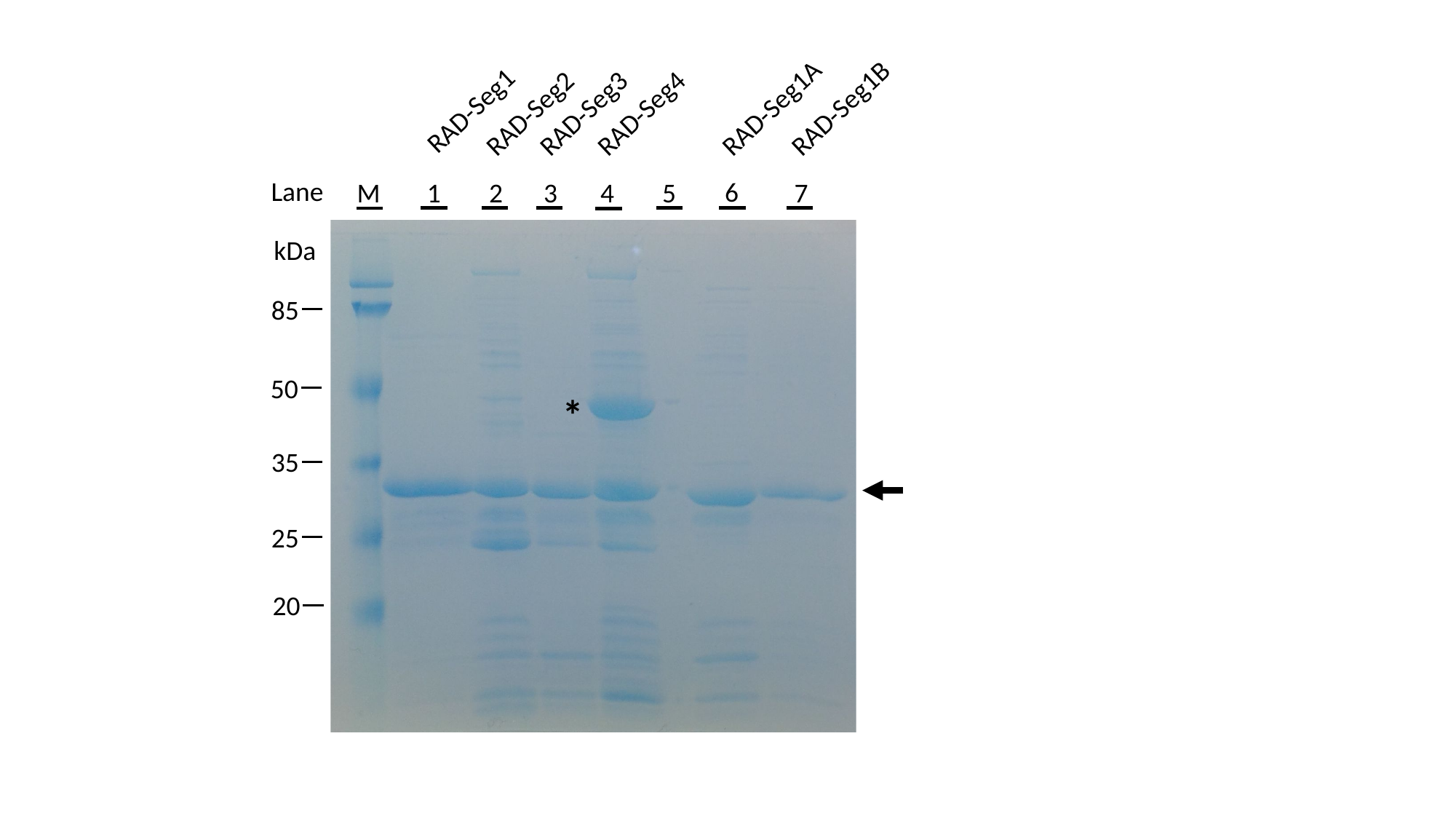

RAD-Seg1
RAD-Seg1B
RAD-Seg2
RAD-Seg1A
RAD-Seg3
RAD-Seg4
Lane
6
7
M
1
2
3
4
5
kDa
85
50
*
35
25
20
