# Supplementary figures and images for: Novel immunotherapeutics against LGR5 to target multiple cancer types
Source: EMBO Mol Med. 2024 Aug 21;16(9):2233–61. doi: 10.1038/s44321-024-00121-2 (PMC11393416; doi:10.1038/s44321-024-00121-2)

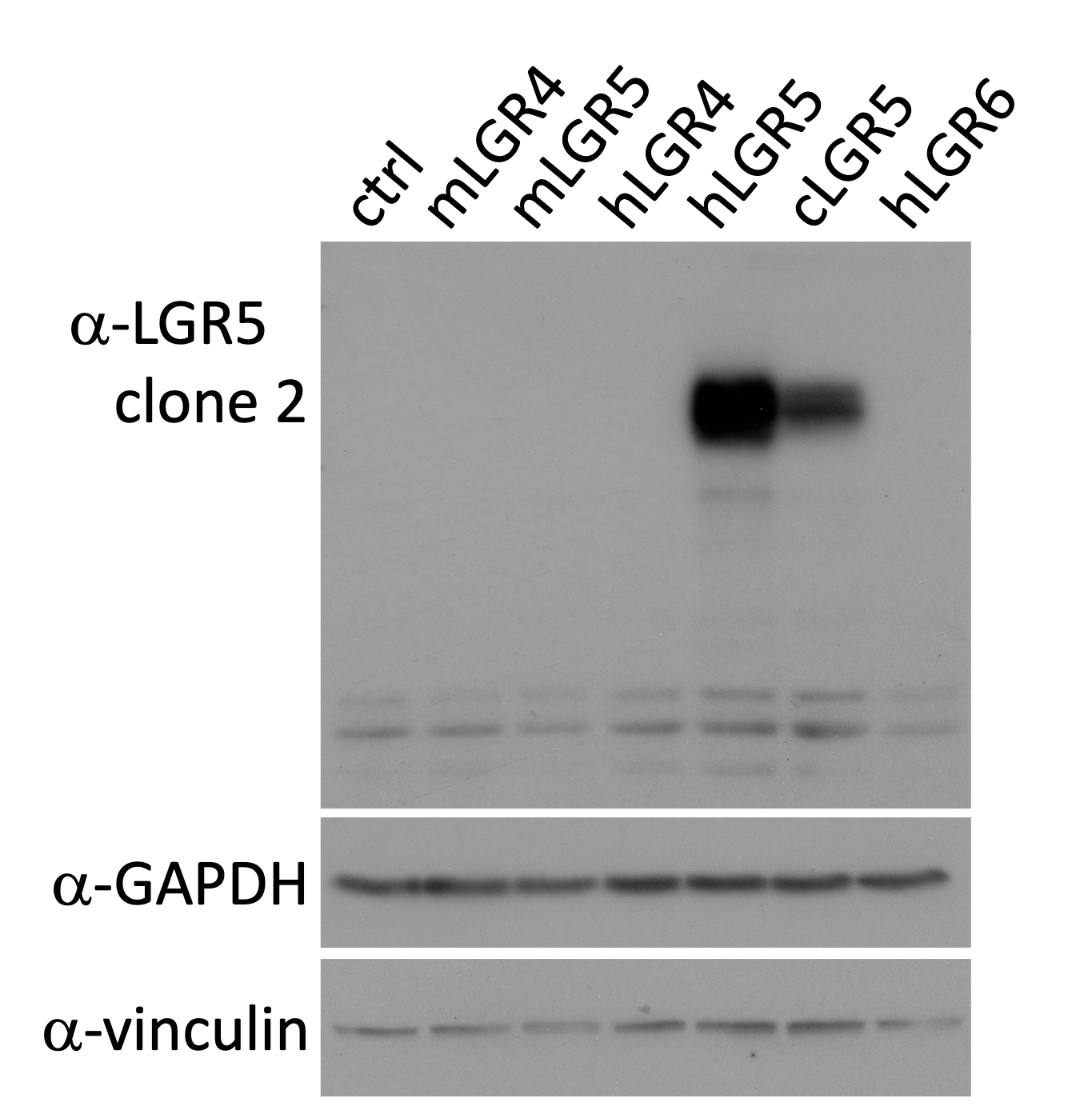

Supplement: Supplementary file 3 — Source data Fig. 1 [file 44321_2024_121_MOESM3_ESM.zip › Figure 1/Figure 1A/Fig1A_Western_Blot.png]

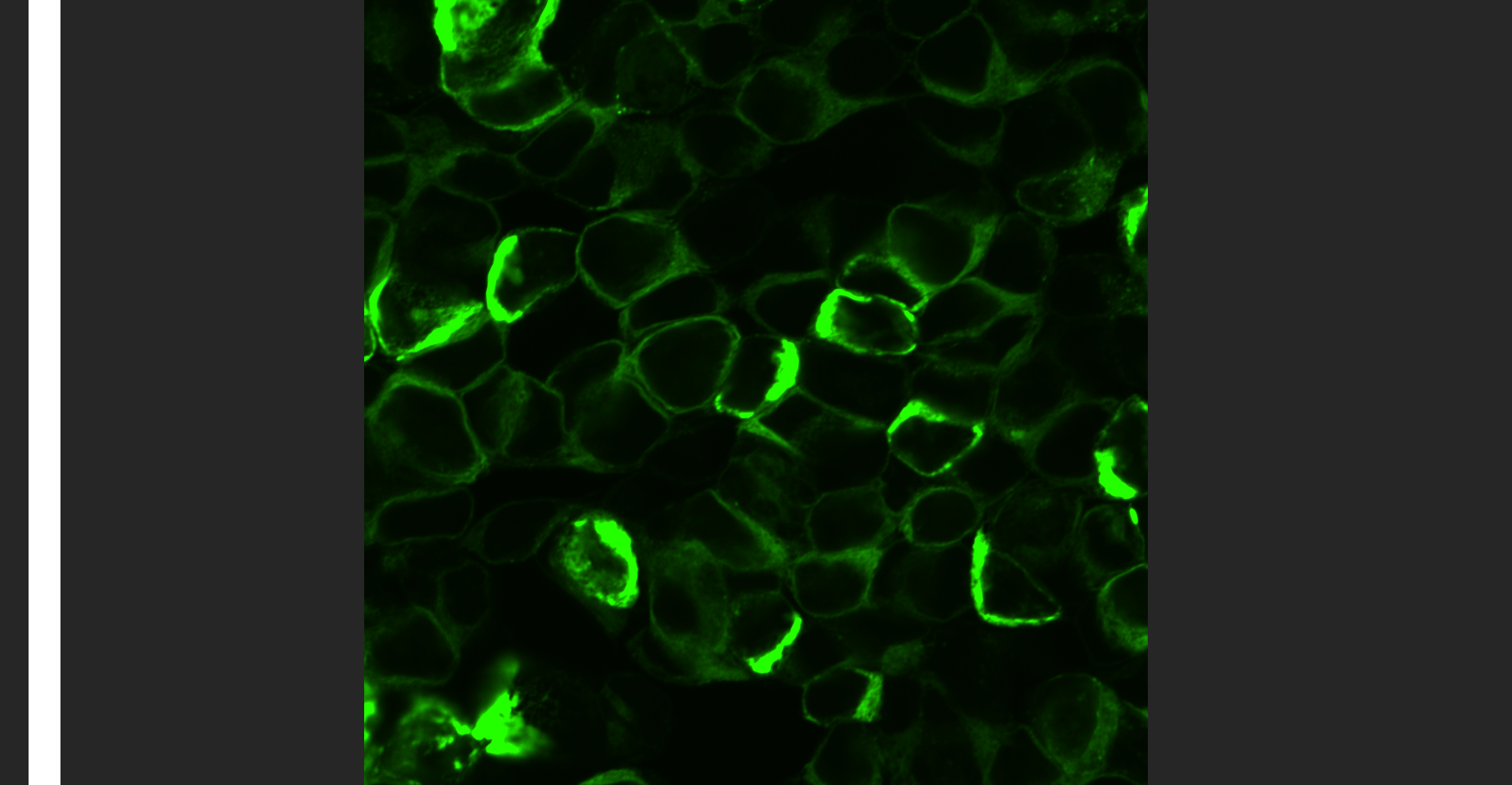

Supplement: Supplementary file 3 — Source data Fig. 1 [file 44321_2024_121_MOESM3_ESM.zip › Figure 1/Figure 1C/hLGR6/20211117 LGR overexpression_hLGR6_mC2_green.tif]

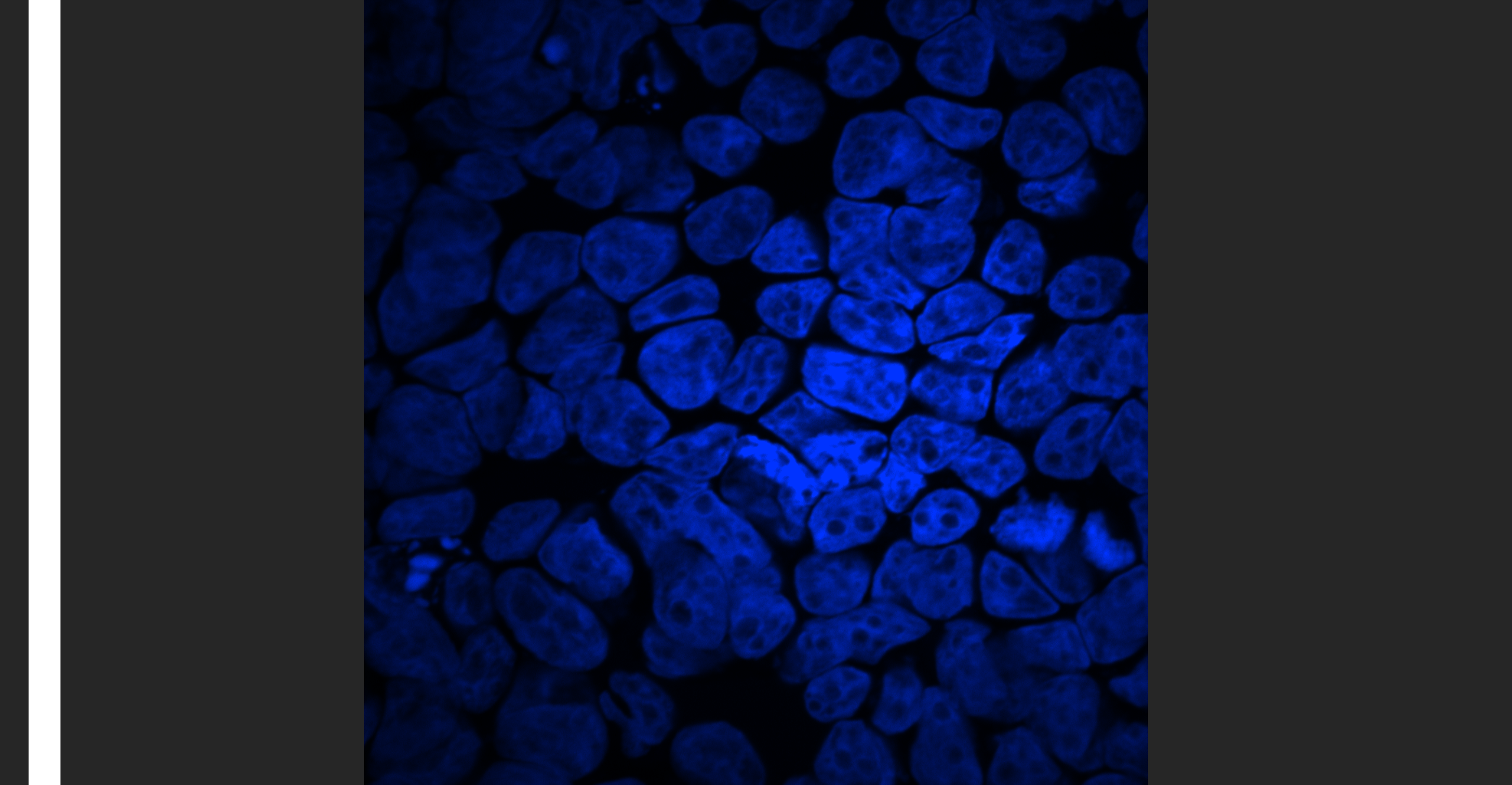

Supplement: Supplementary file 3 — Source data Fig. 1 [file 44321_2024_121_MOESM3_ESM.zip › Figure 1/Figure 1C/hLGR6/20211117 LGR overexpression_hLGR6_mC2_DAPI.tif]

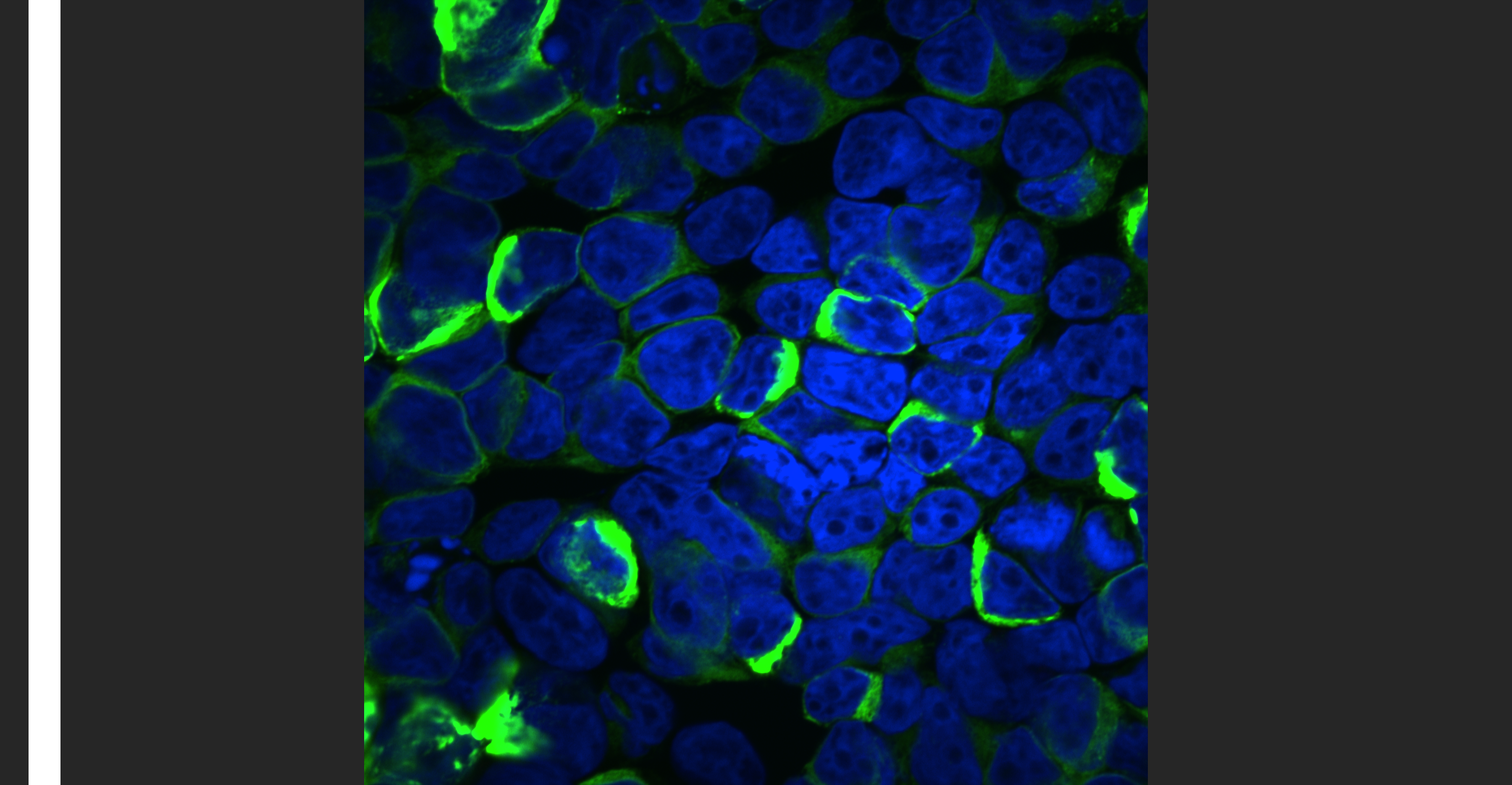

Supplement: Supplementary file 3 — Source data Fig. 1 [file 44321_2024_121_MOESM3_ESM.zip › Figure 1/Figure 1C/hLGR6/20211117 LGR overexpression_hLGR6_mC2_merge.tif]

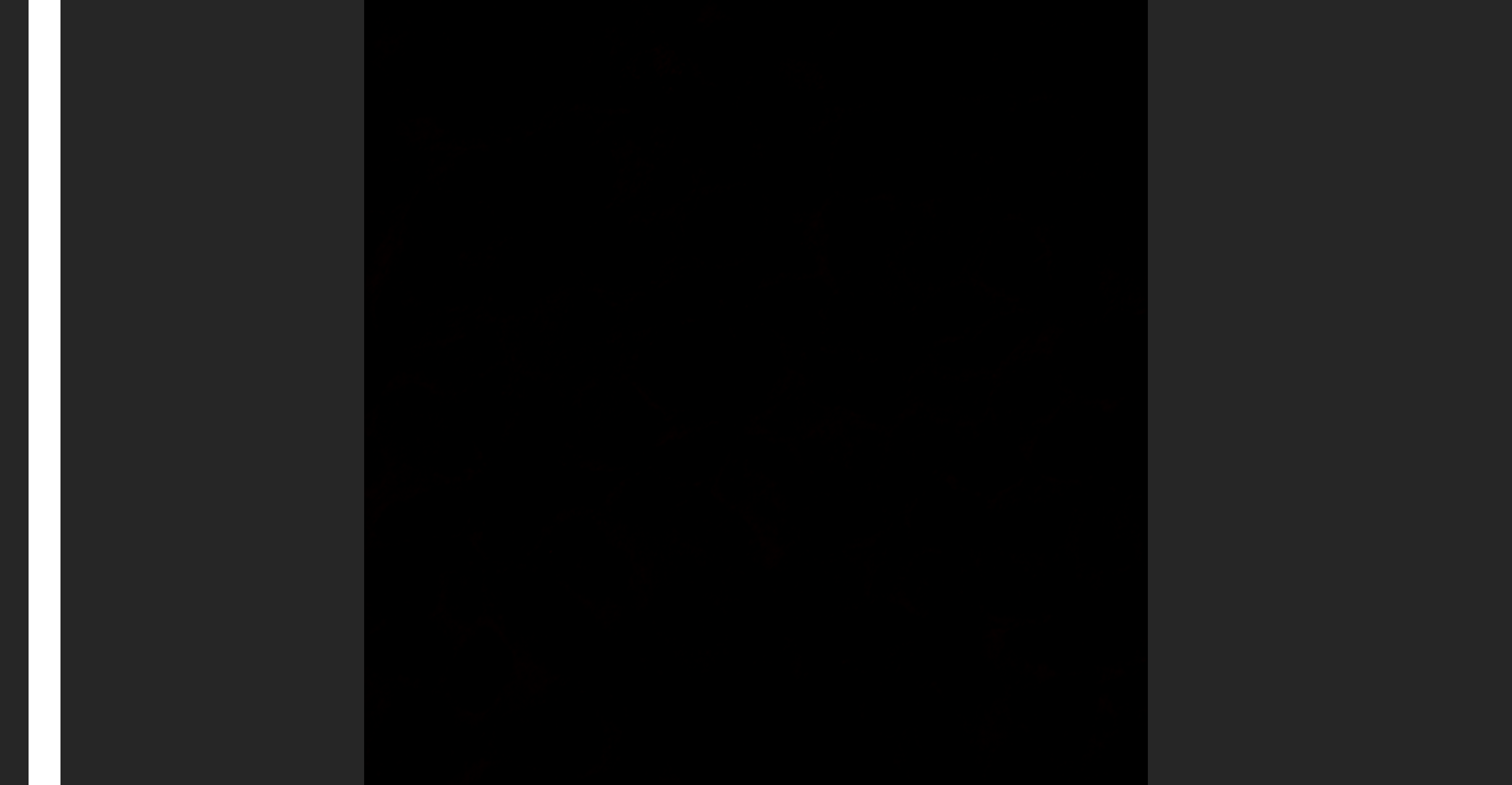

Supplement: Supplementary file 3 — Source data Fig. 1 [file 44321_2024_121_MOESM3_ESM.zip › Figure 1/Figure 1C/hLGR6/20211117 LGR overexpression_hLGR6_mC2_Cy5.tif]

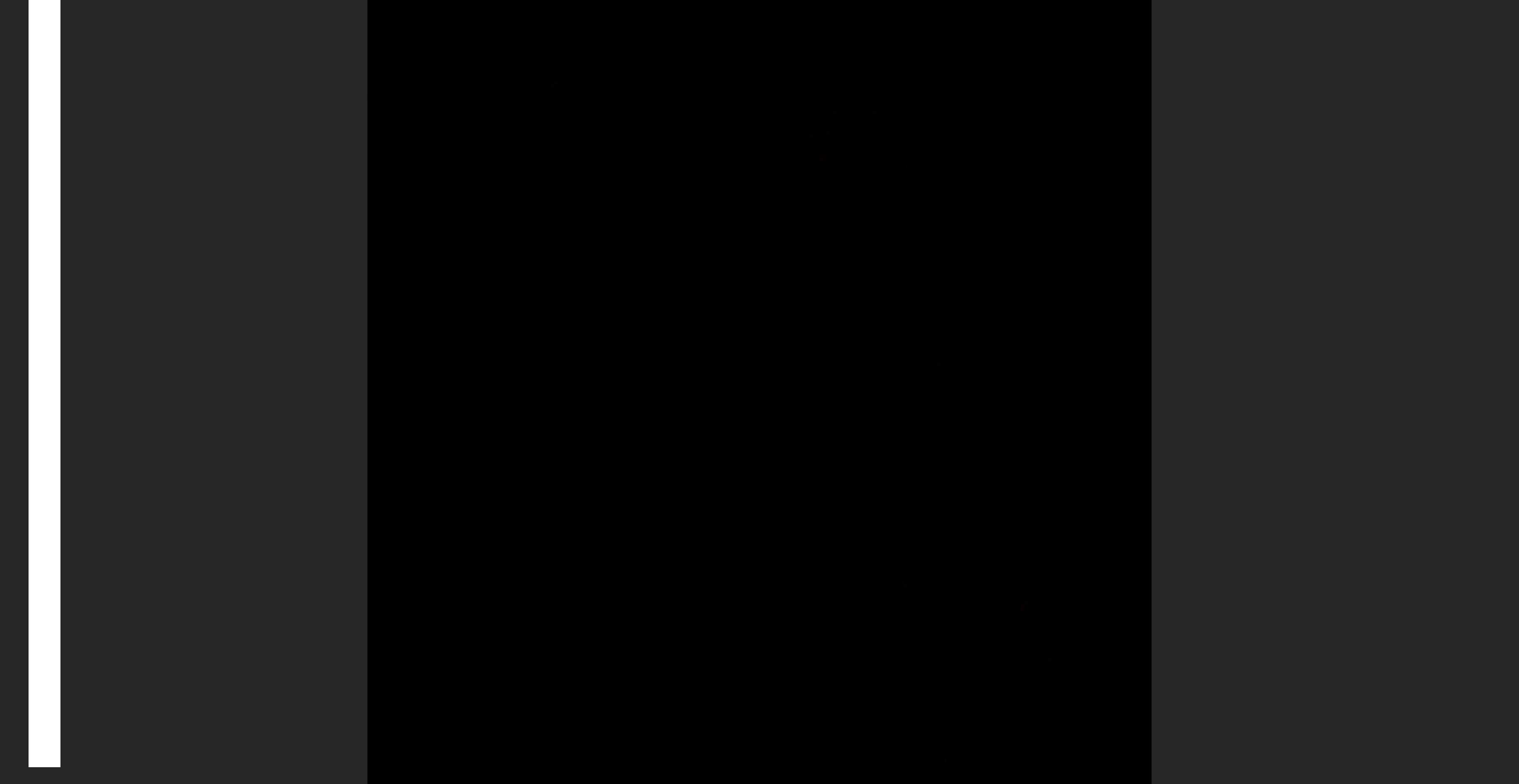

Supplement: Supplementary file 3 — Source data Fig. 1 [file 44321_2024_121_MOESM3_ESM.zip › Figure 1/Figure 1C/hLGR5_Frag1A/20211117 LGR overexpression_hLGR5_mC2_pep_Cy5.tif]

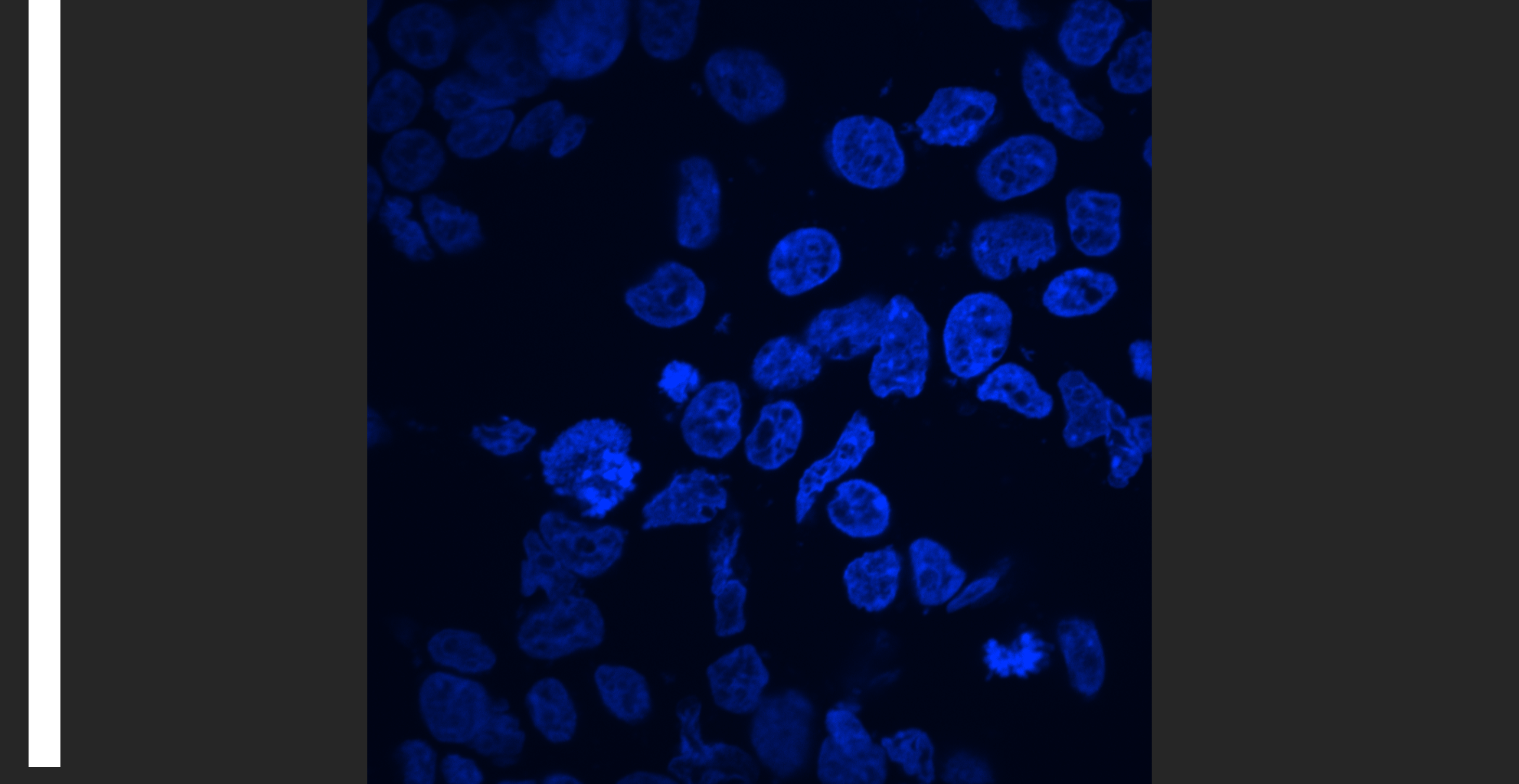

Supplement: Supplementary file 3 — Source data Fig. 1 [file 44321_2024_121_MOESM3_ESM.zip › Figure 1/Figure 1C/hLGR5_Frag1A/20211117 LGR overexpression_hLGR5_mC2_pep_DAPI.tif]

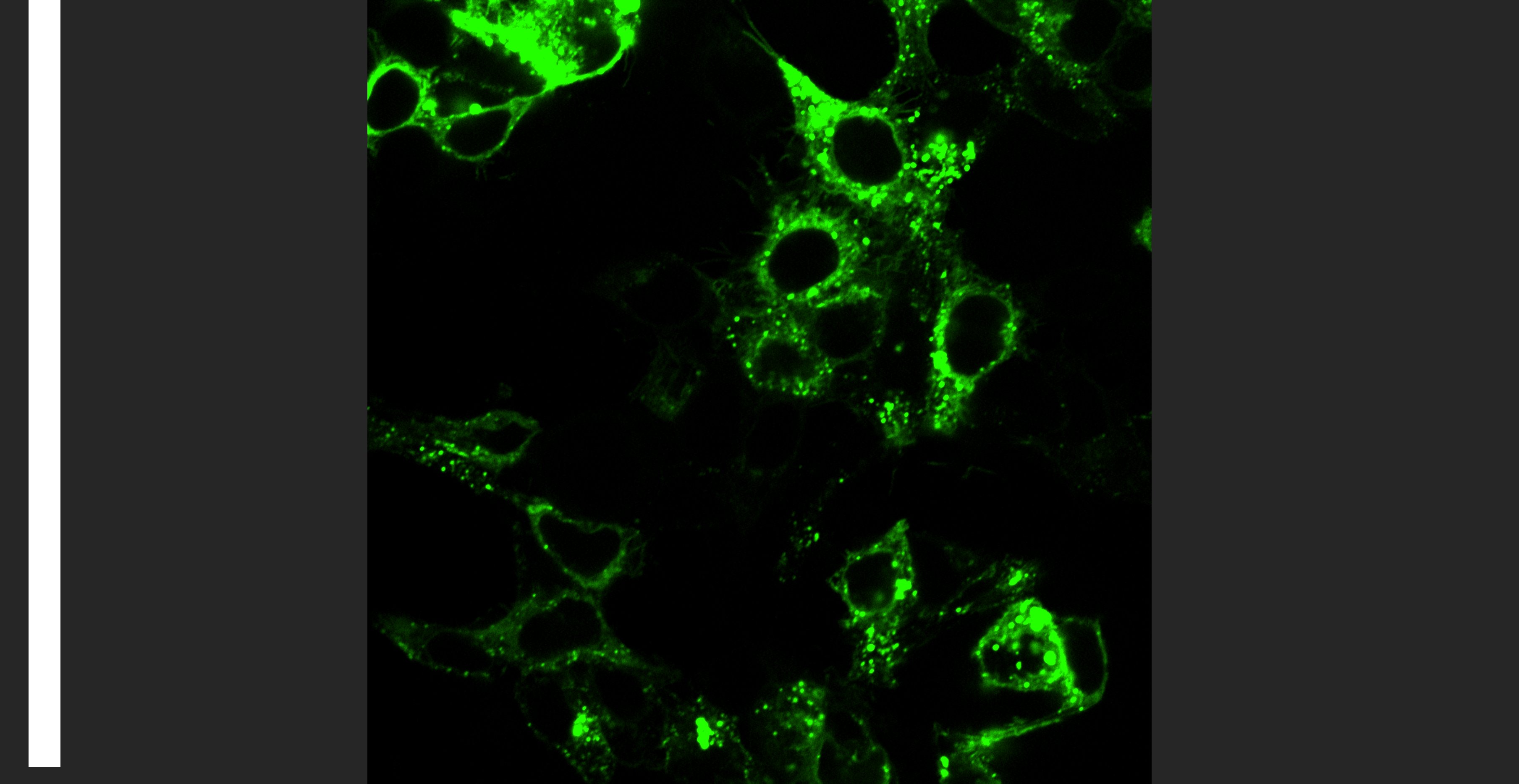

Supplement: Supplementary file 3 — Source data Fig. 1 [file 44321_2024_121_MOESM3_ESM.zip › Figure 1/Figure 1C/hLGR5_Frag1A/20211117 LGR overexpression_hLGR5_mC2_pep_green.tif]

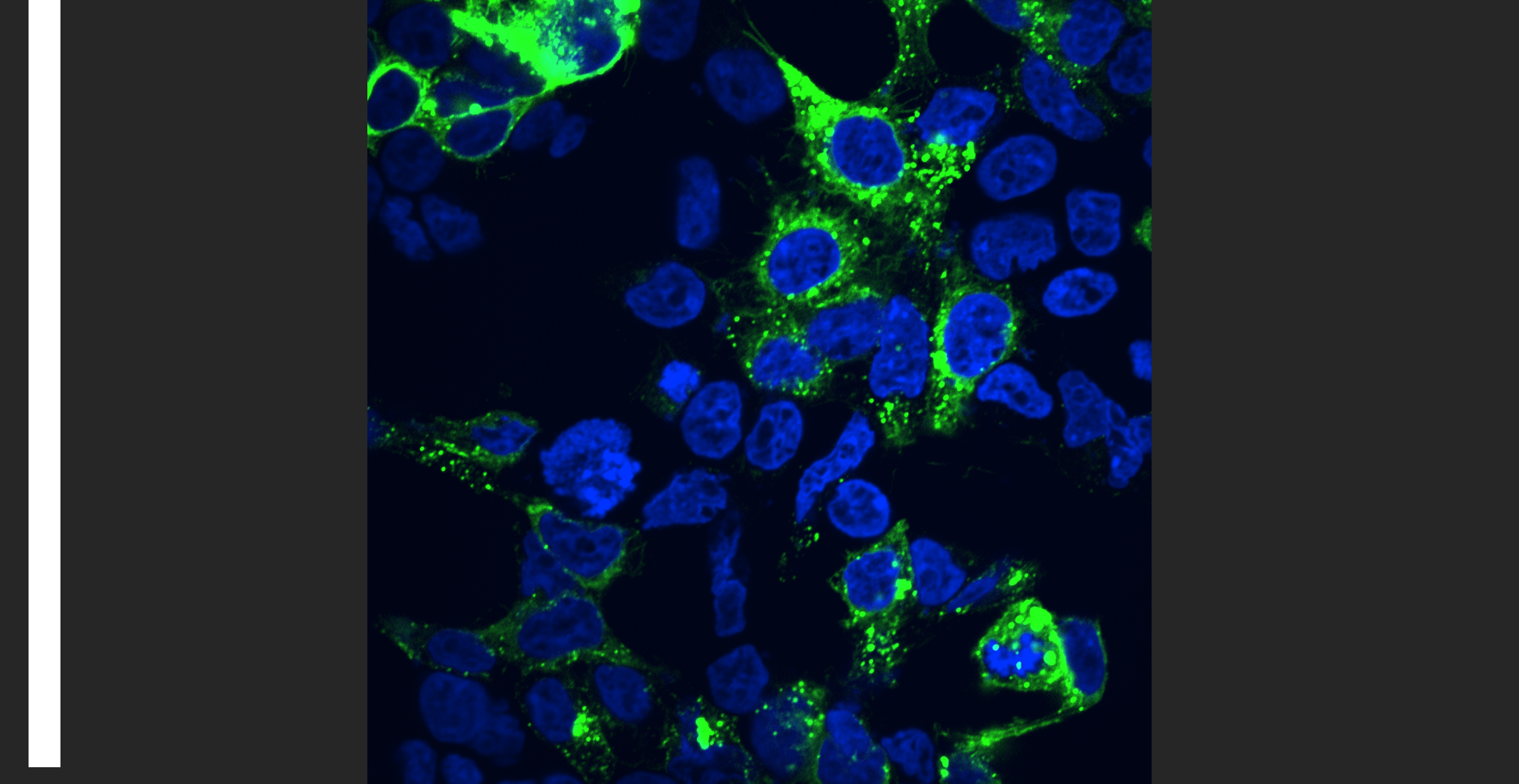

Supplement: Supplementary file 3 — Source data Fig. 1 [file 44321_2024_121_MOESM3_ESM.zip › Figure 1/Figure 1C/hLGR5_Frag1A/20211117 LGR overexpression_hLGR5_mC2_pep_merge.tif]

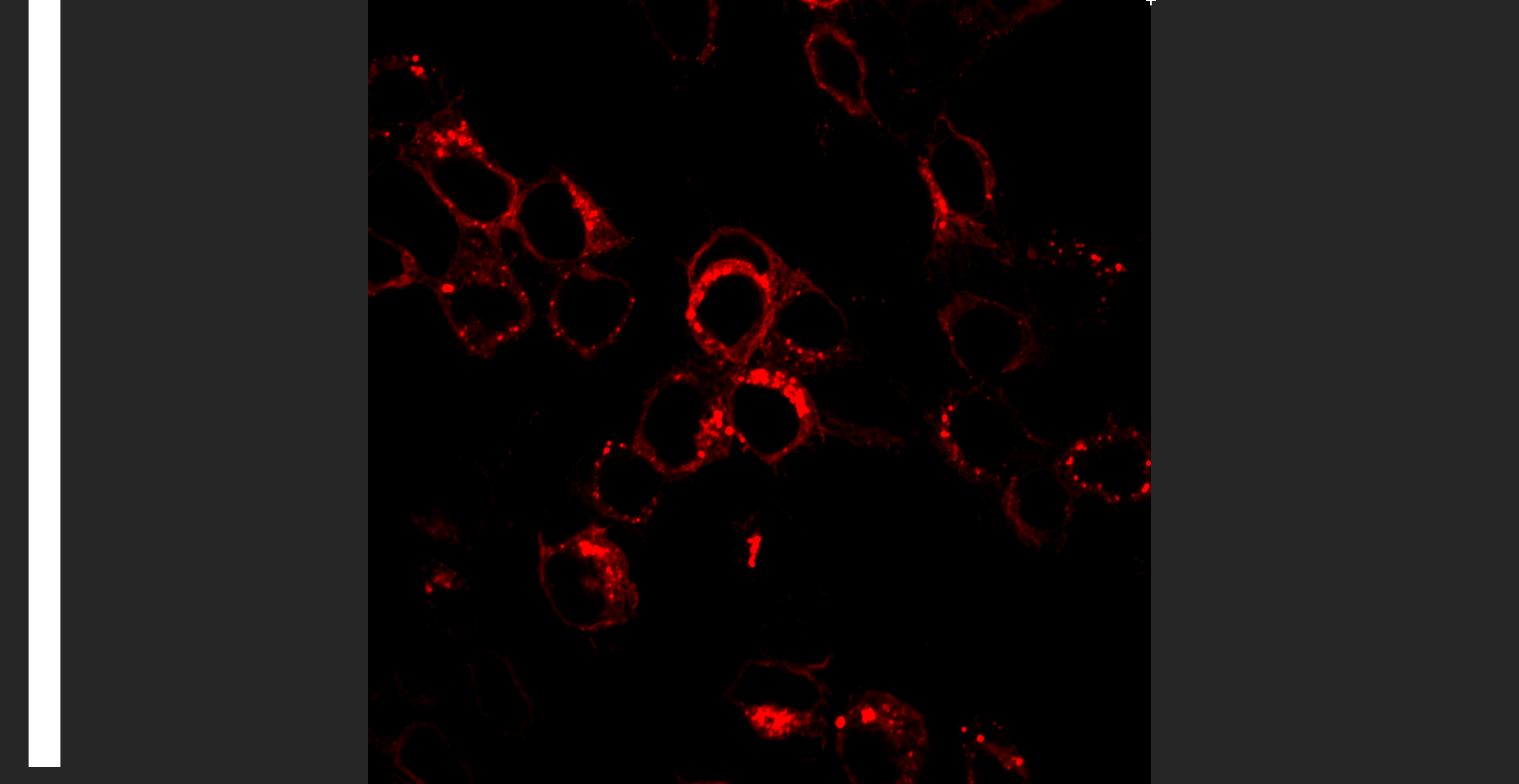

Supplement: Supplementary file 3 — Source data Fig. 1 [file 44321_2024_121_MOESM3_ESM.zip › Figure 1/Figure 1C/hLGR5/20211117 LGR overexpression_hLGR5_mC2_Cy5.tif]

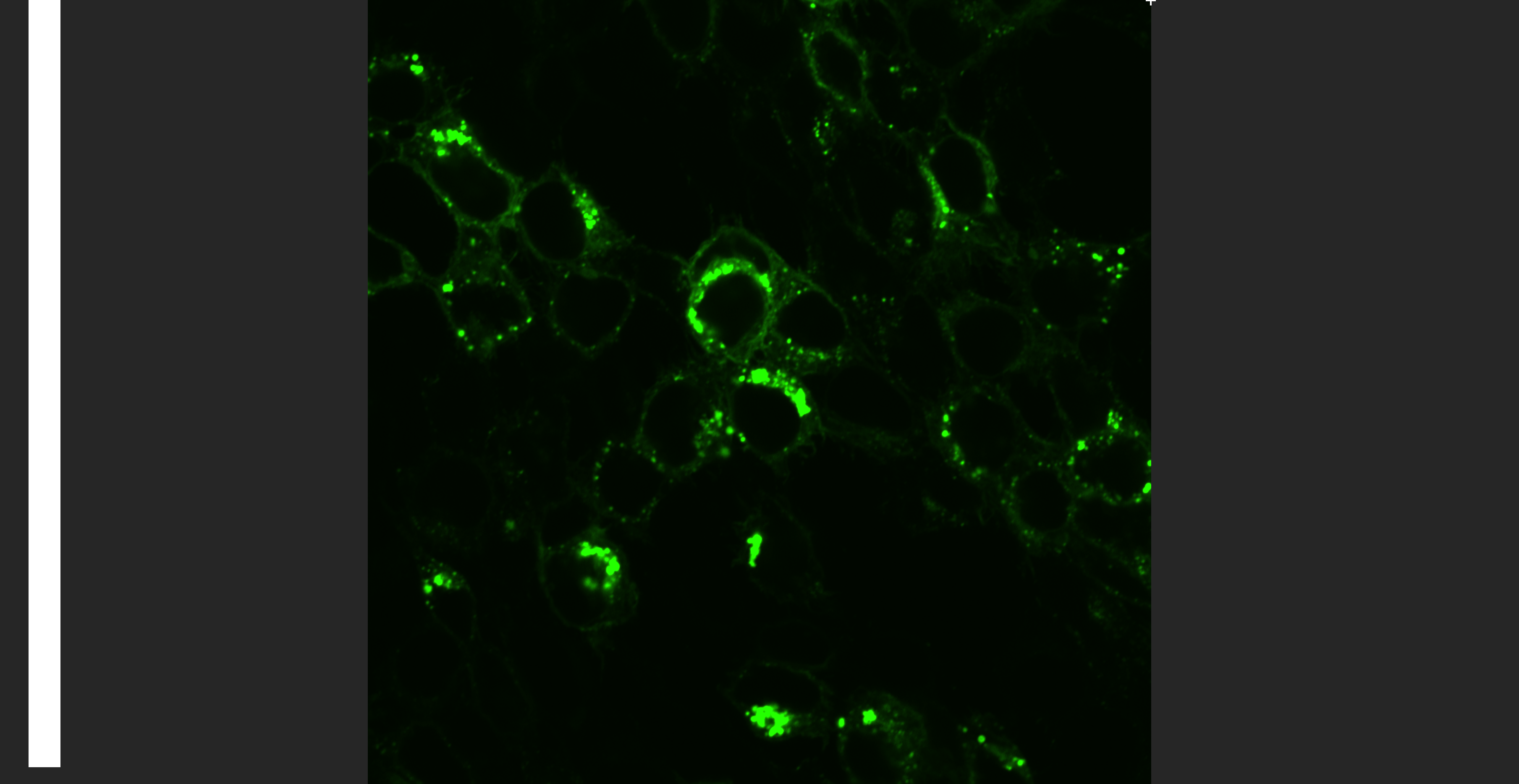

Supplement: Supplementary file 3 — Source data Fig. 1 [file 44321_2024_121_MOESM3_ESM.zip › Figure 1/Figure 1C/hLGR5/20211117 LGR overexpression_hLGR5_mC2_green.tif]

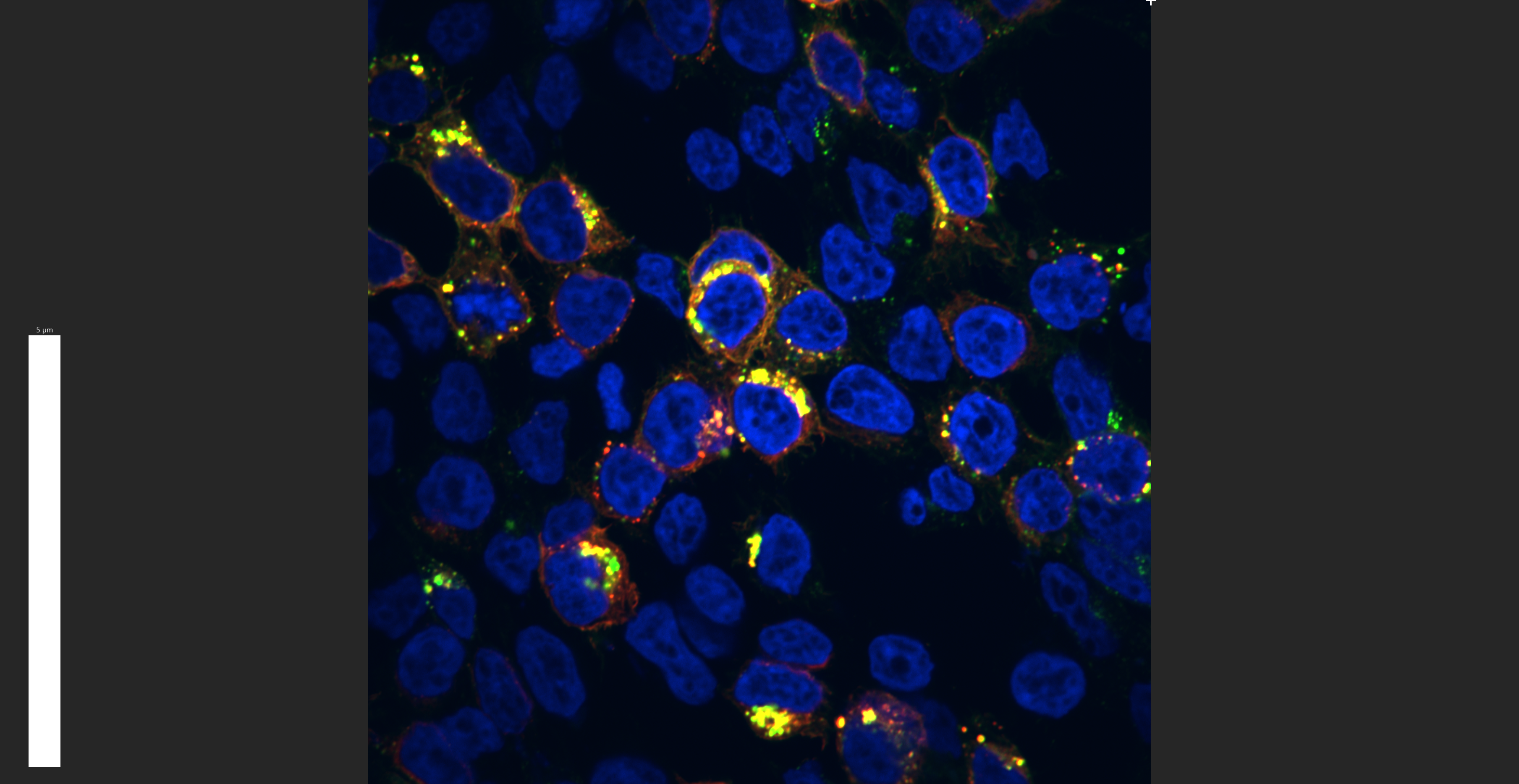

Supplement: Supplementary file 3 — Source data Fig. 1 [file 44321_2024_121_MOESM3_ESM.zip › Figure 1/Figure 1C/hLGR5/20211117 LGR overexpression_hLGR5_mC2_merge.tif]

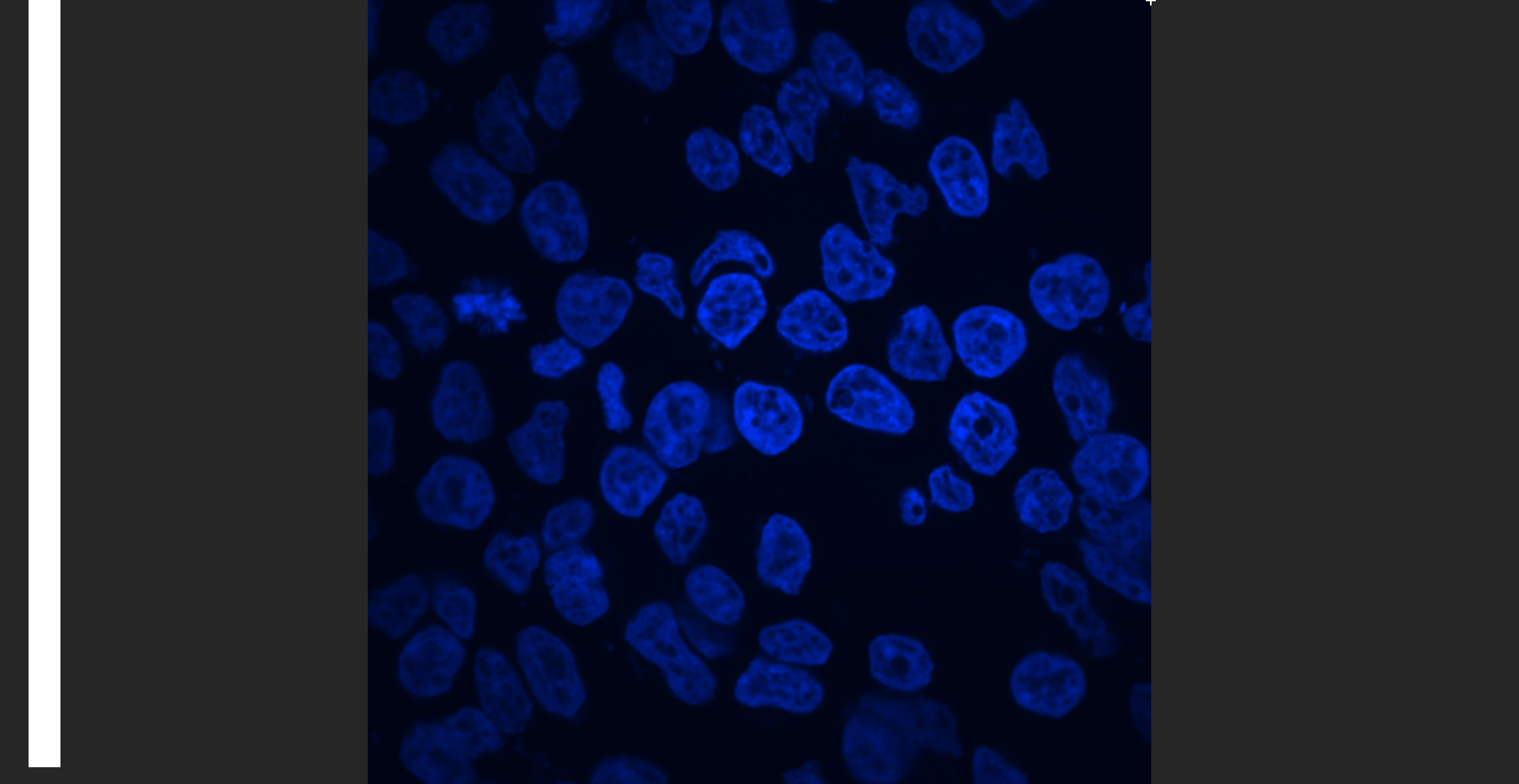

Supplement: Supplementary file 3 — Source data Fig. 1 [file 44321_2024_121_MOESM3_ESM.zip › Figure 1/Figure 1C/hLGR5/20211117 LGR overexpression_hLGR5_mC2_DAPI.tif]

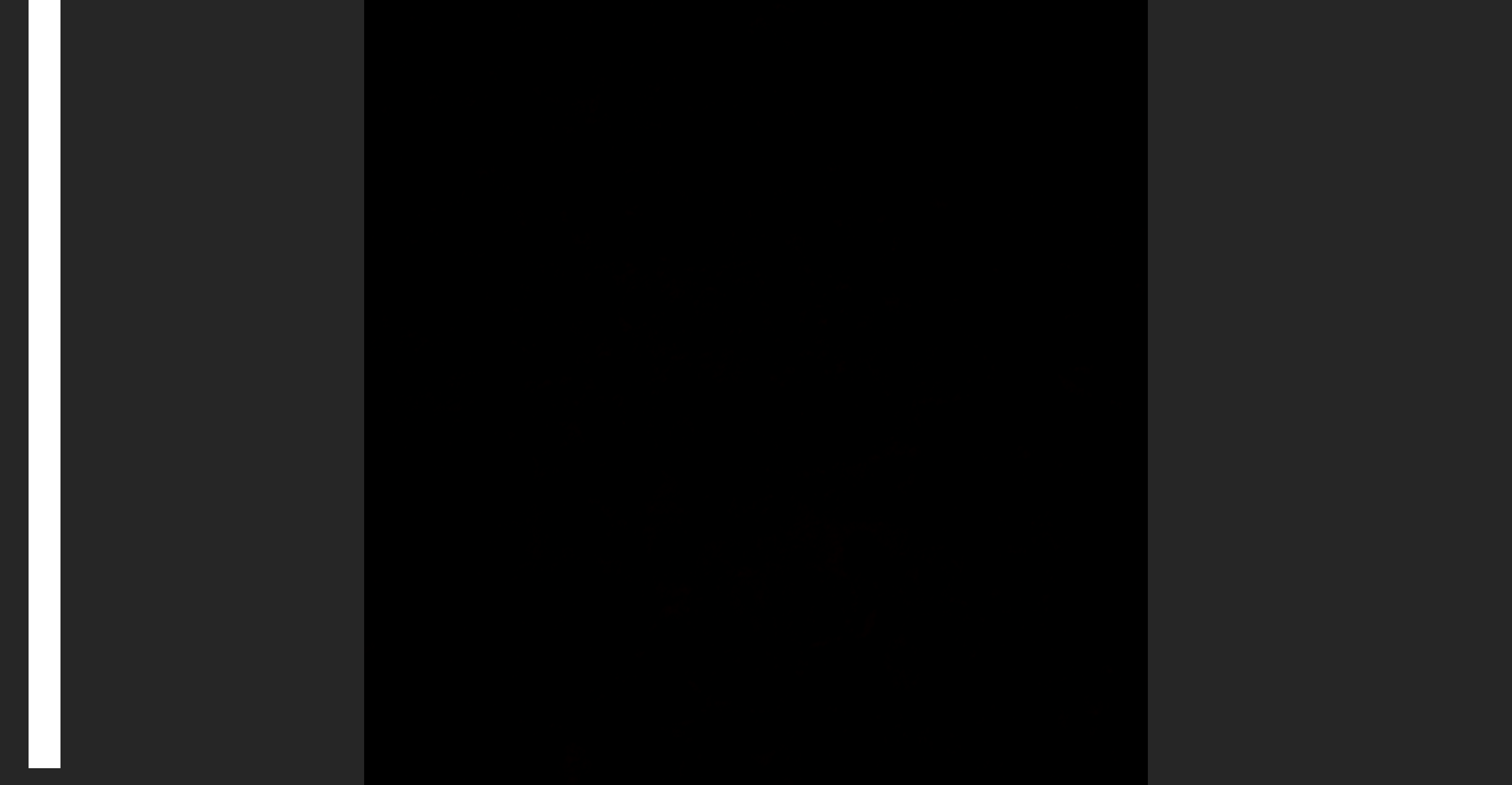

Supplement: Supplementary file 3 — Source data Fig. 1 [file 44321_2024_121_MOESM3_ESM.zip › Figure 1/Figure 1C/hLGR4/20211117 LGR overexpression_hLGR4_mC2_Cy5.tif]

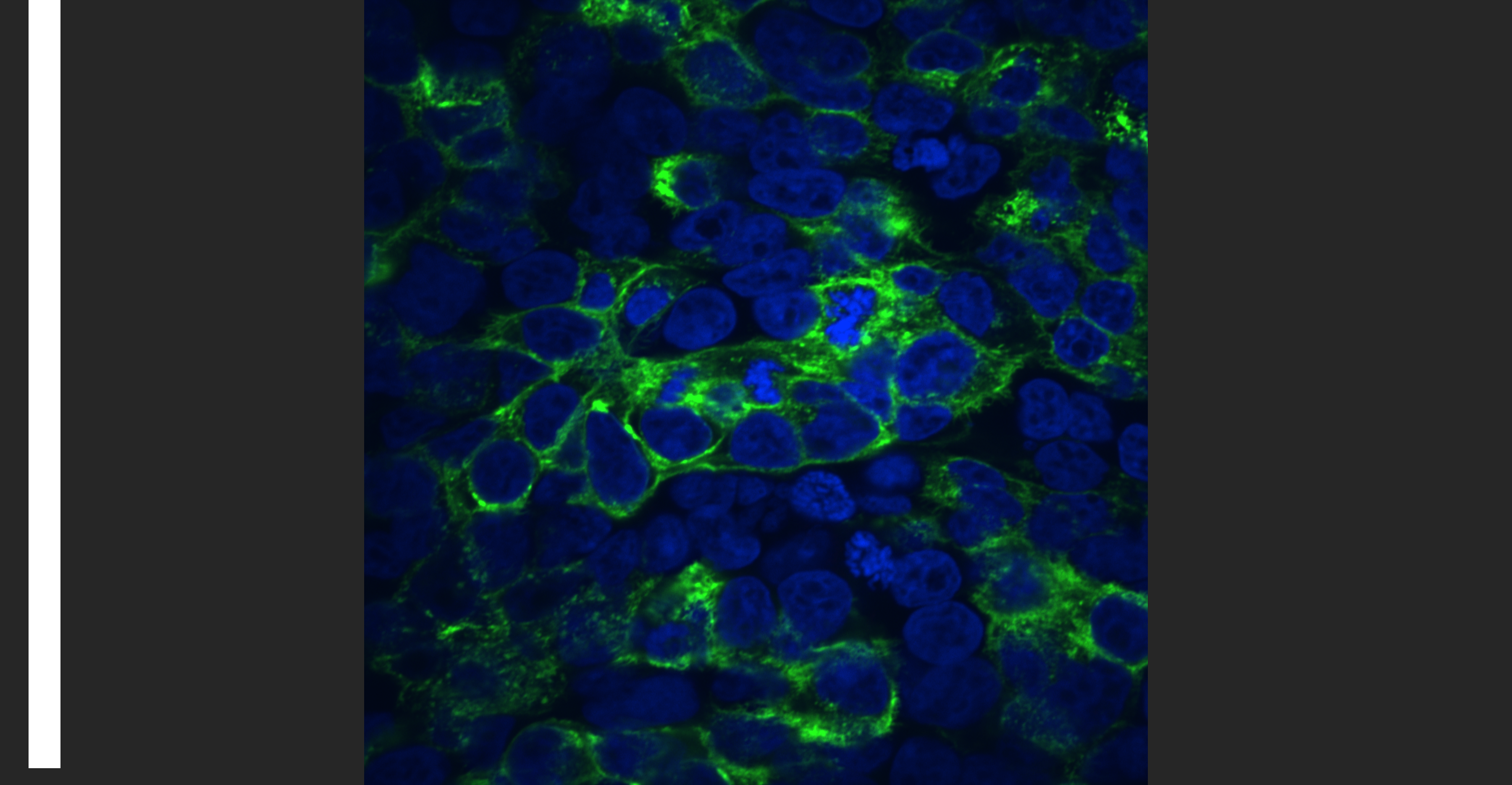

Supplement: Supplementary file 3 — Source data Fig. 1 [file 44321_2024_121_MOESM3_ESM.zip › Figure 1/Figure 1C/hLGR4/20211117 LGR overexpression_hLGR4_mC2_merge.tif]

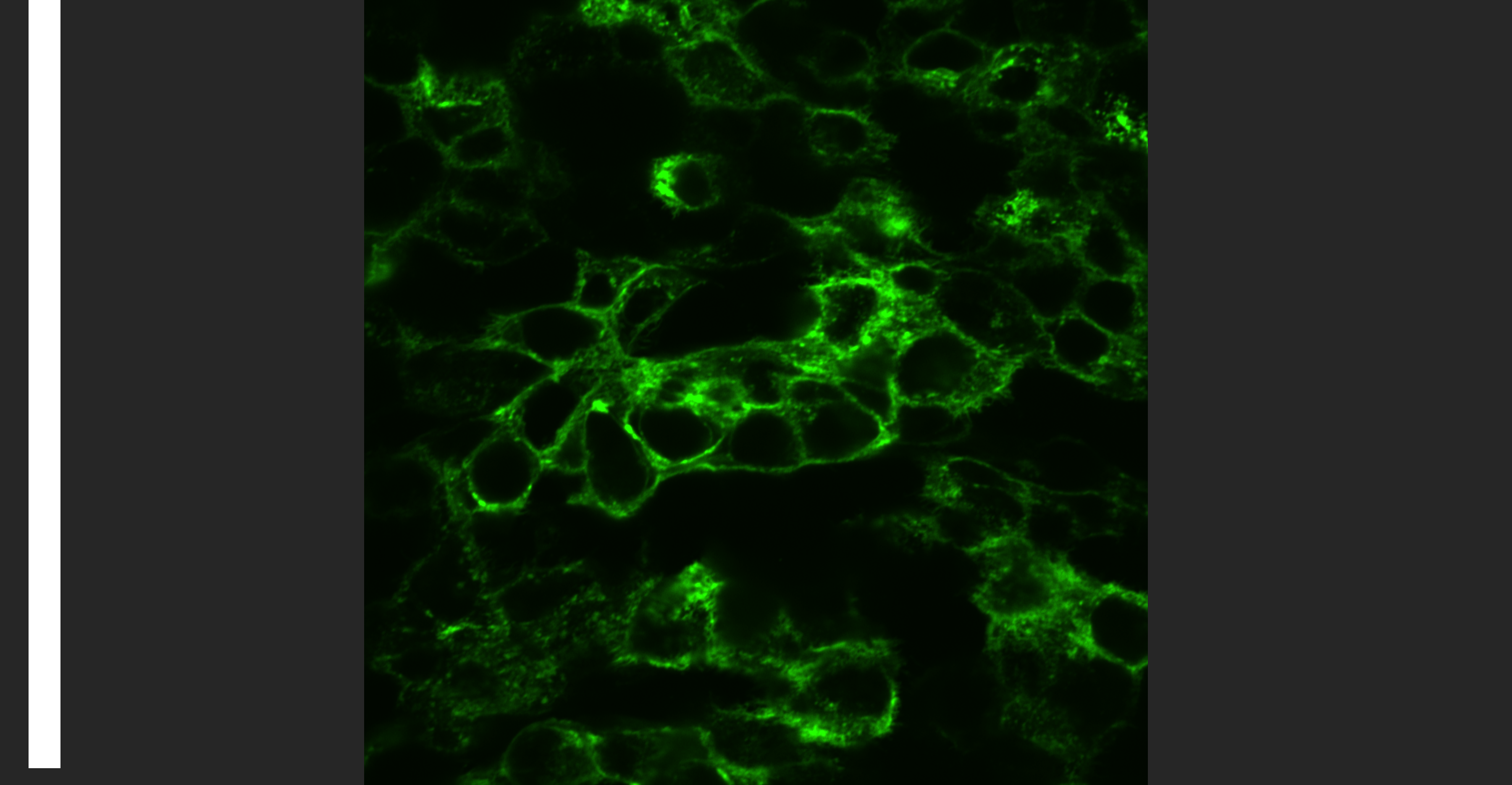

Supplement: Supplementary file 3 — Source data Fig. 1 [file 44321_2024_121_MOESM3_ESM.zip › Figure 1/Figure 1C/hLGR4/20211117 LGR overexpression_hLGR4_mC2_green.tif]

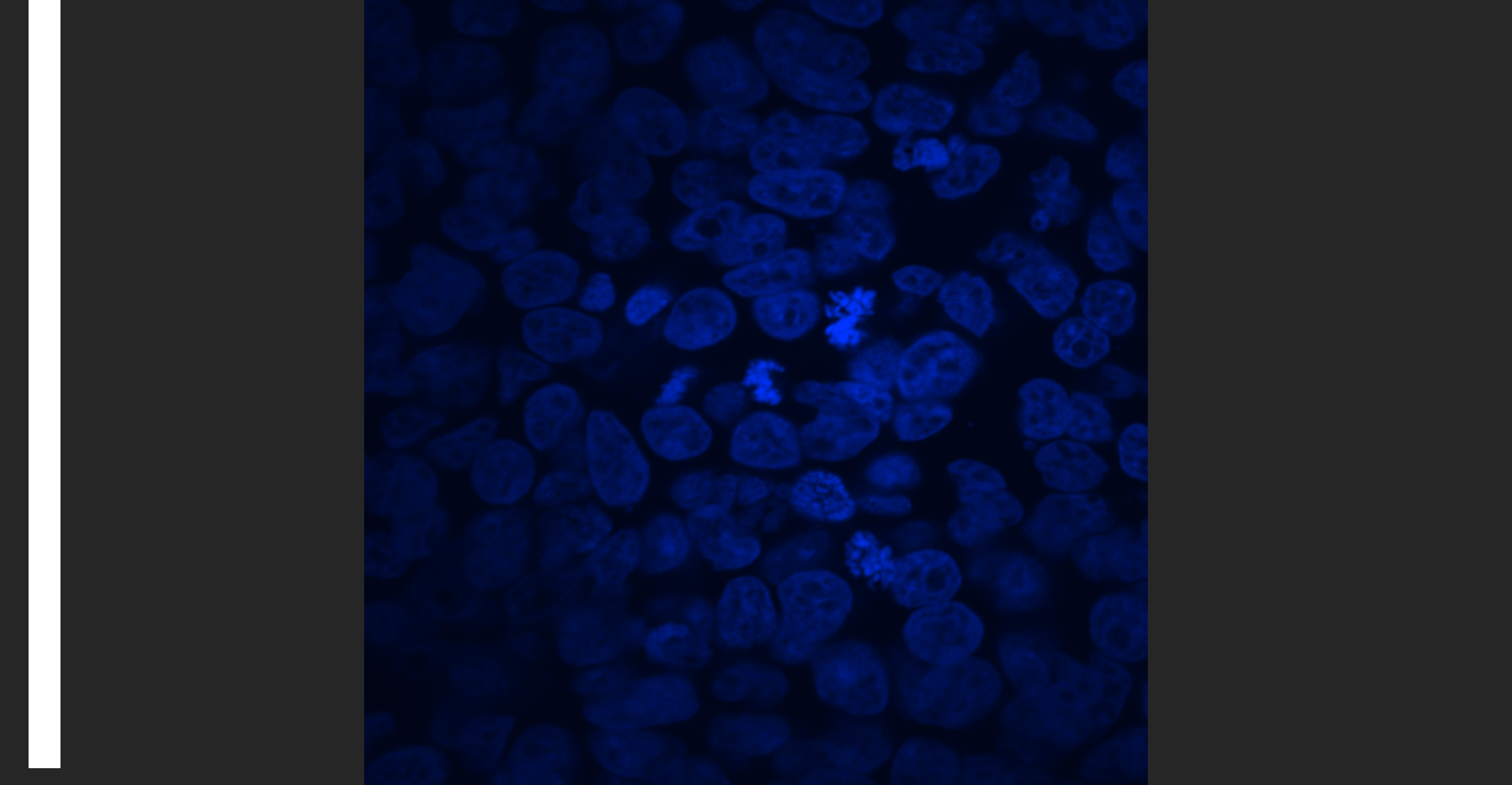

Supplement: Supplementary file 3 — Source data Fig. 1 [file 44321_2024_121_MOESM3_ESM.zip › Figure 1/Figure 1C/hLGR4/20211117 LGR overexpression_hLGR4_mC2_DAPI.tif]

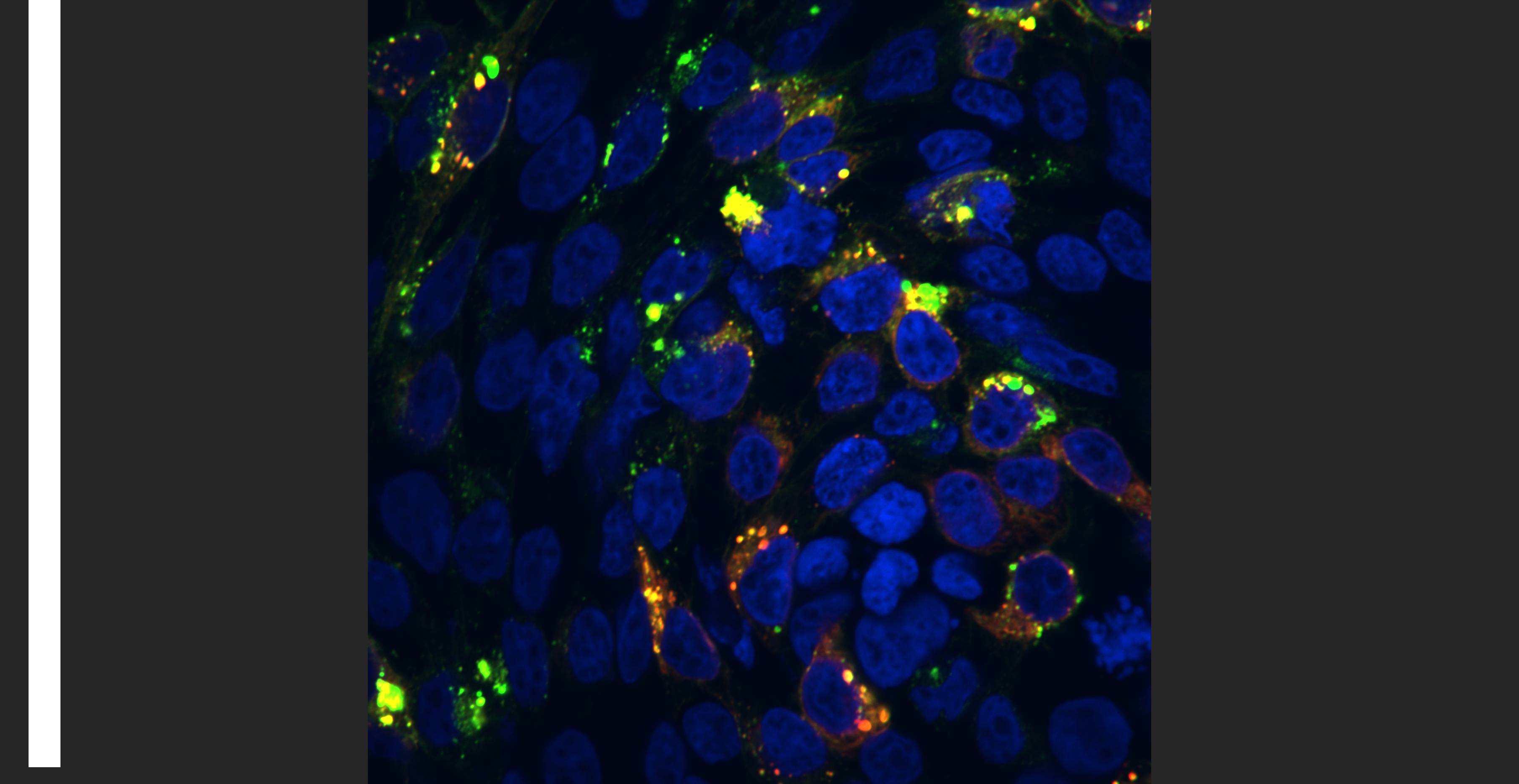

Supplement: Supplementary file 3 — Source data Fig. 1 [file 44321_2024_121_MOESM3_ESM.zip › Figure 1/Figure 1C/cynoLGR5/20211117 LGR overexpression_cynoLGR5_mC2_rep2_merge.tif]

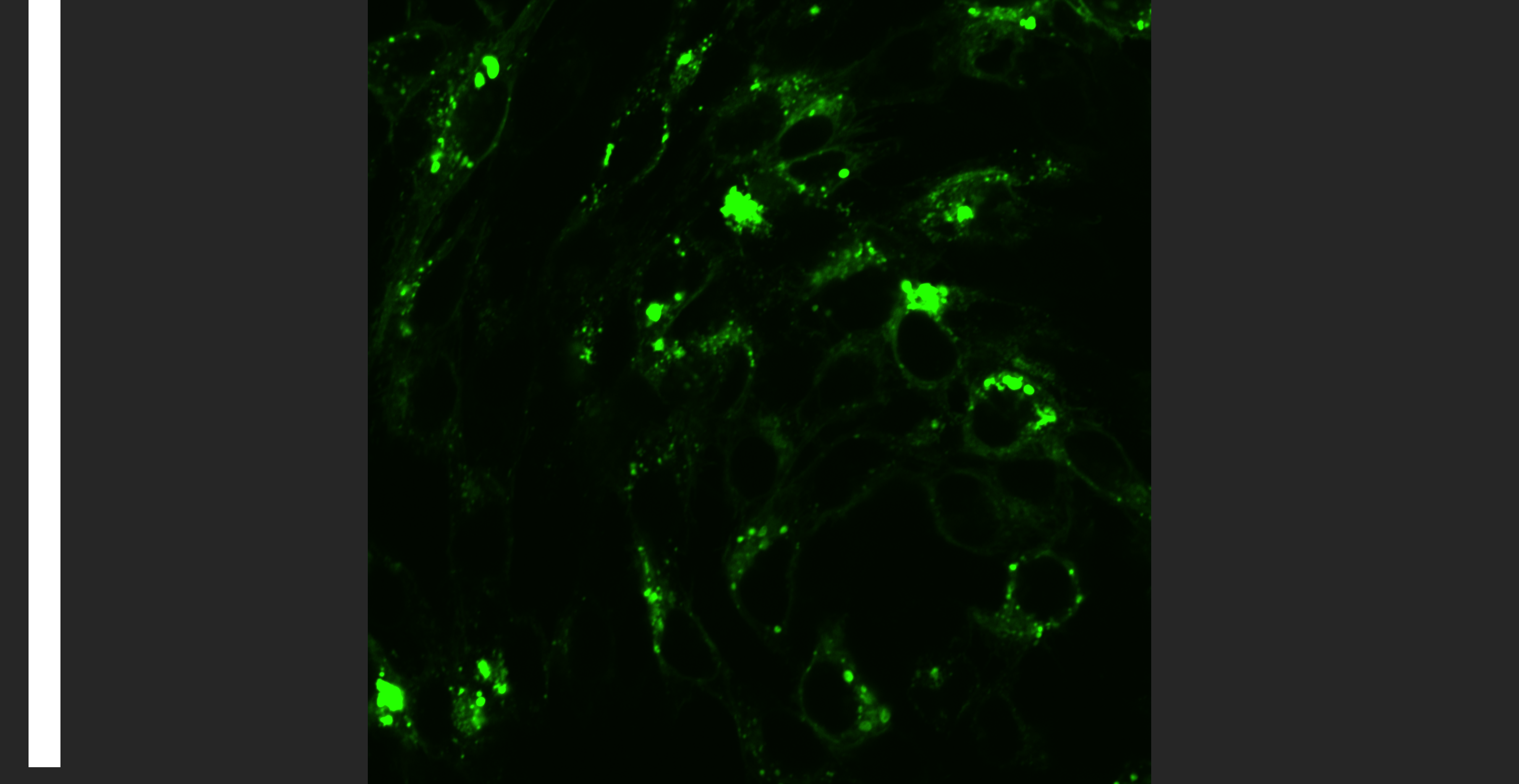

Supplement: Supplementary file 3 — Source data Fig. 1 [file 44321_2024_121_MOESM3_ESM.zip › Figure 1/Figure 1C/cynoLGR5/20211117 LGR overexpression_cynoLGR5_mC2_rep2_green.tif]

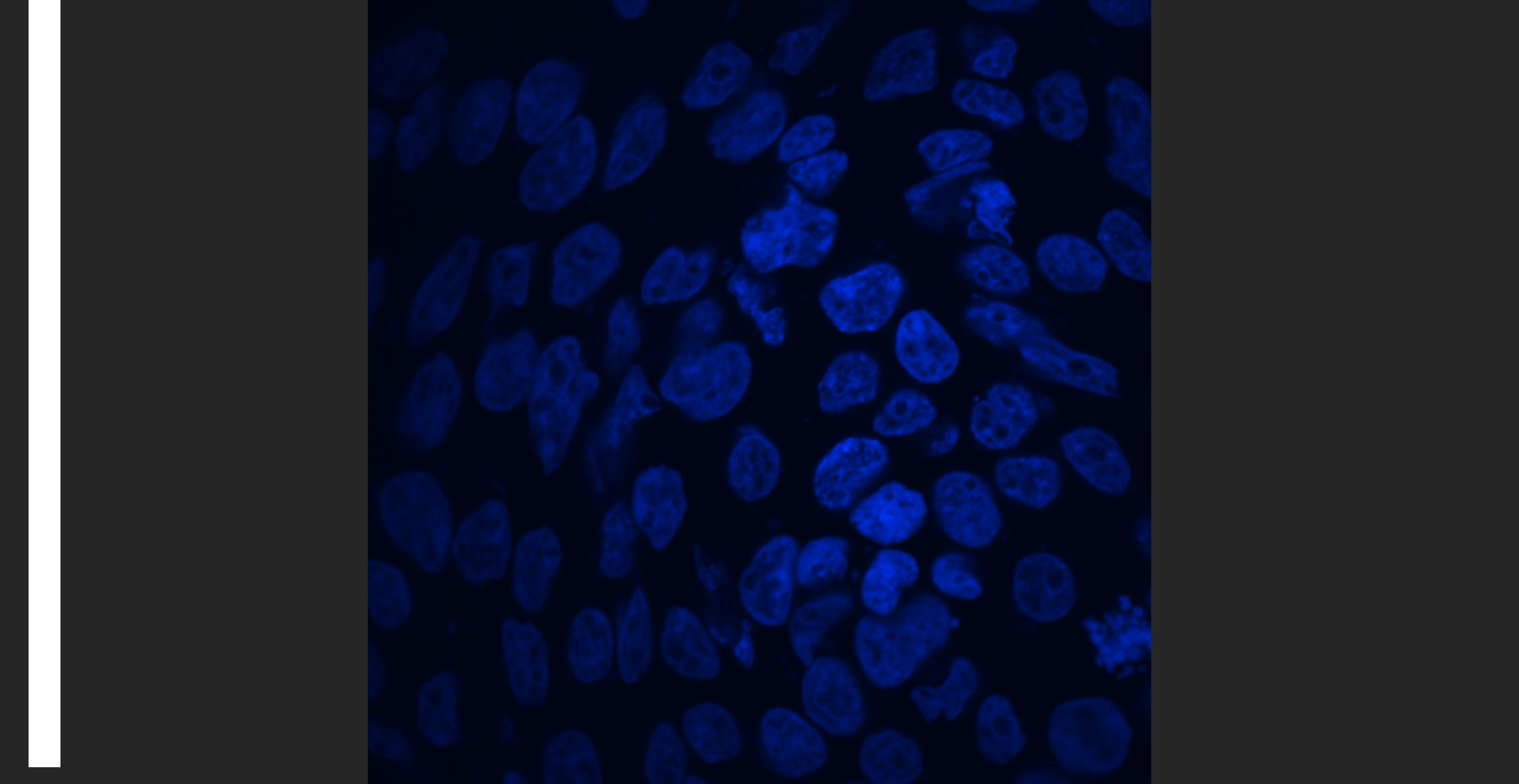

Supplement: Supplementary file 3 — Source data Fig. 1 [file 44321_2024_121_MOESM3_ESM.zip › Figure 1/Figure 1C/cynoLGR5/20211117 LGR overexpression_cynoLGR5_mC2_rep2_DAPI.tif]

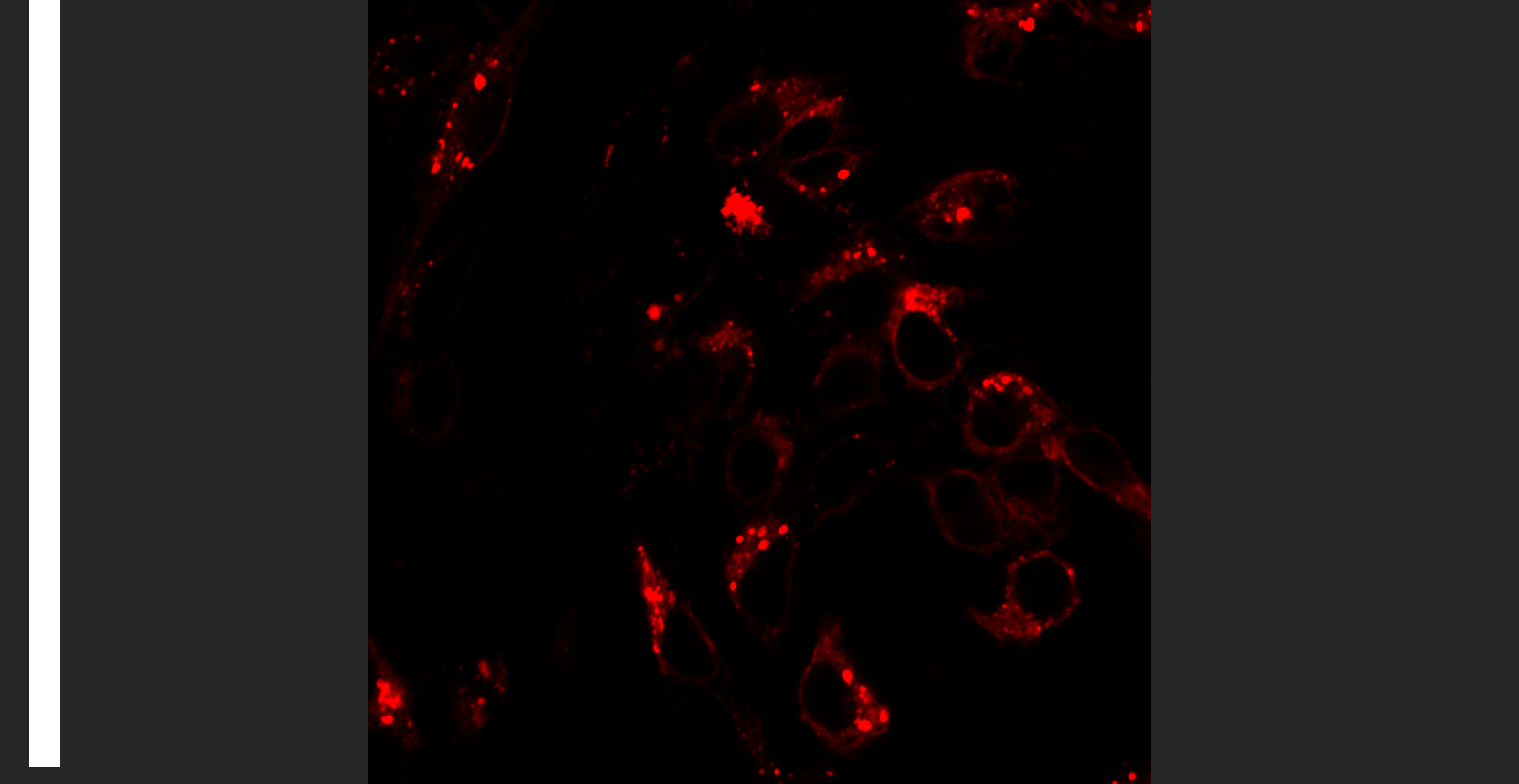

Supplement: Supplementary file 3 — Source data Fig. 1 [file 44321_2024_121_MOESM3_ESM.zip › Figure 1/Figure 1C/cynoLGR5/20211117 LGR overexpression_cynoLGR5_mC2_rep2_Cy5.tif]

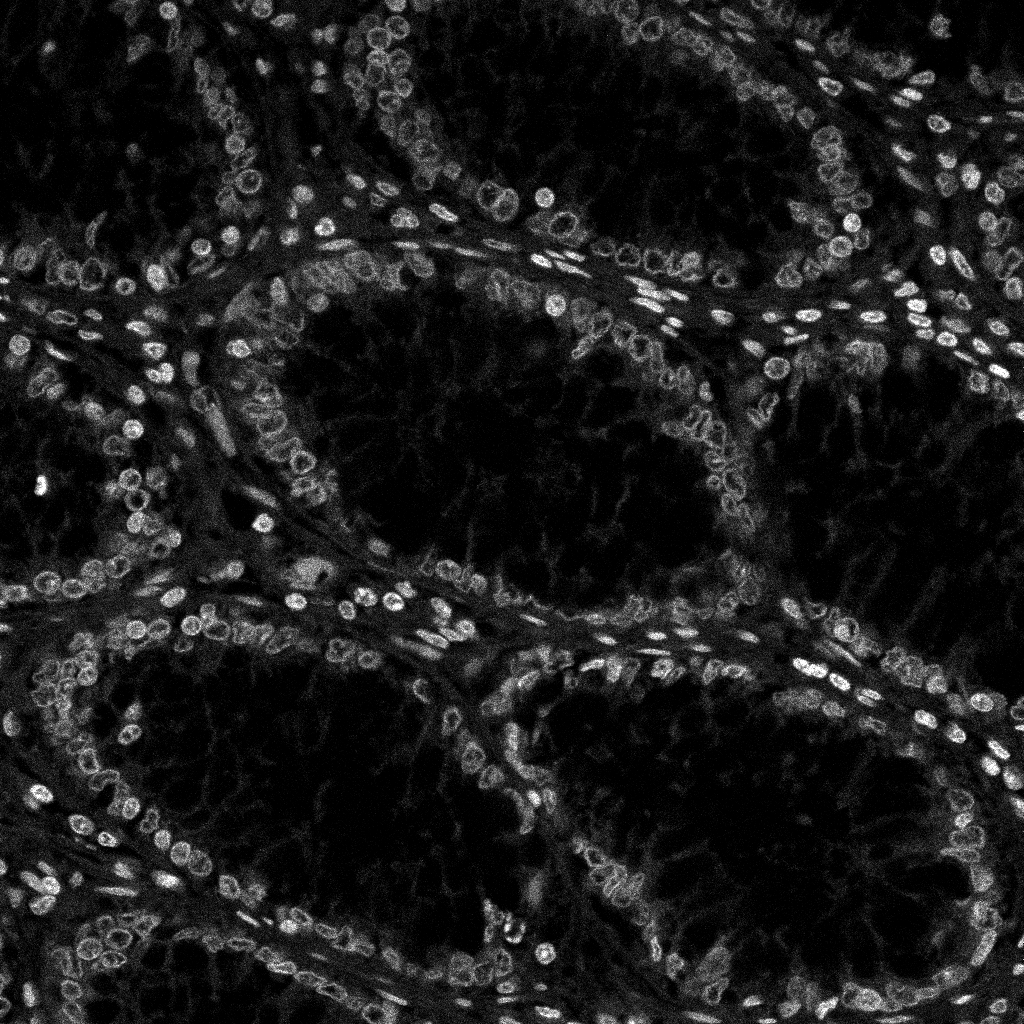

Supplement: Supplementary file 4 — Source data Fig. 2 [file 44321_2024_121_MOESM4_ESM.zip › Figure 2/Figure 2A/Sample 1 dysplastic.tif]

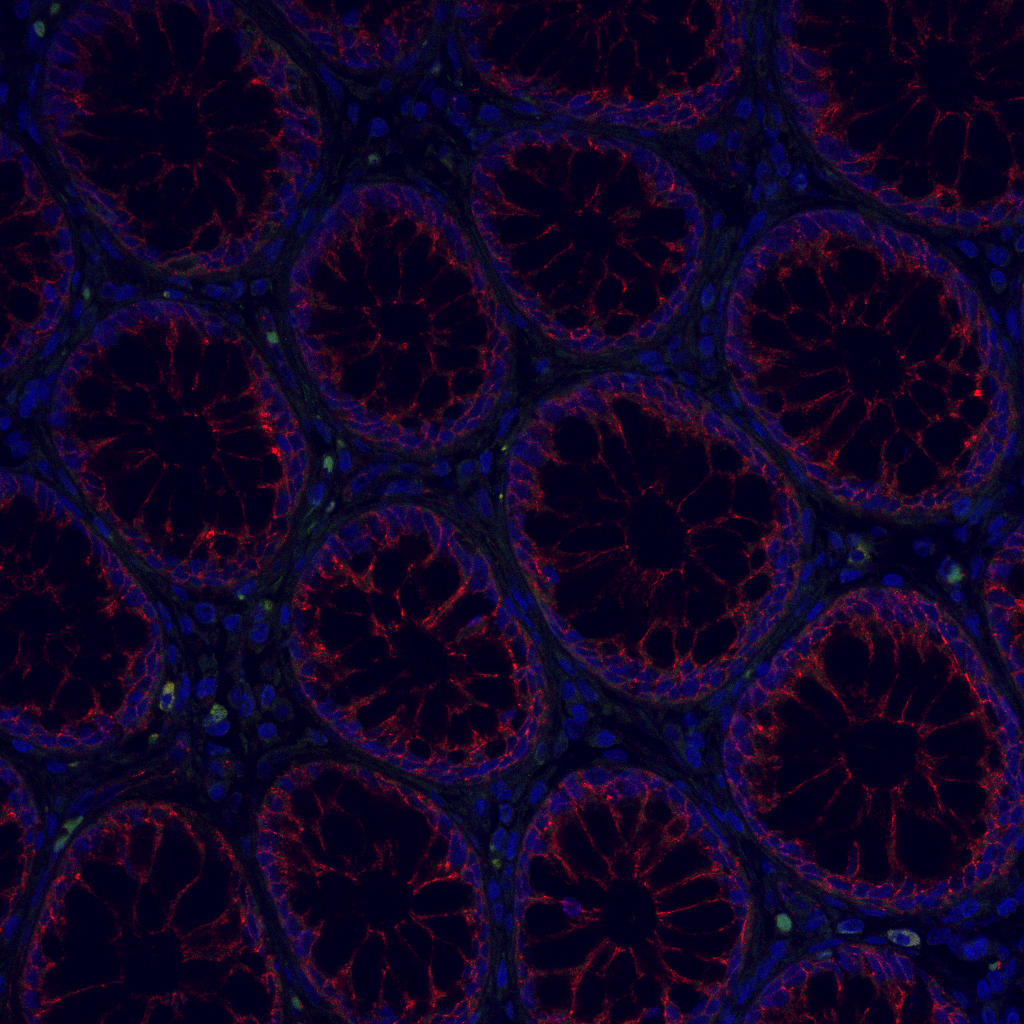

Supplement: Supplementary file 4 — Source data Fig. 2 [file 44321_2024_121_MOESM4_ESM.zip › Figure 2/Figure 2A/Sample 2 normal.tif]

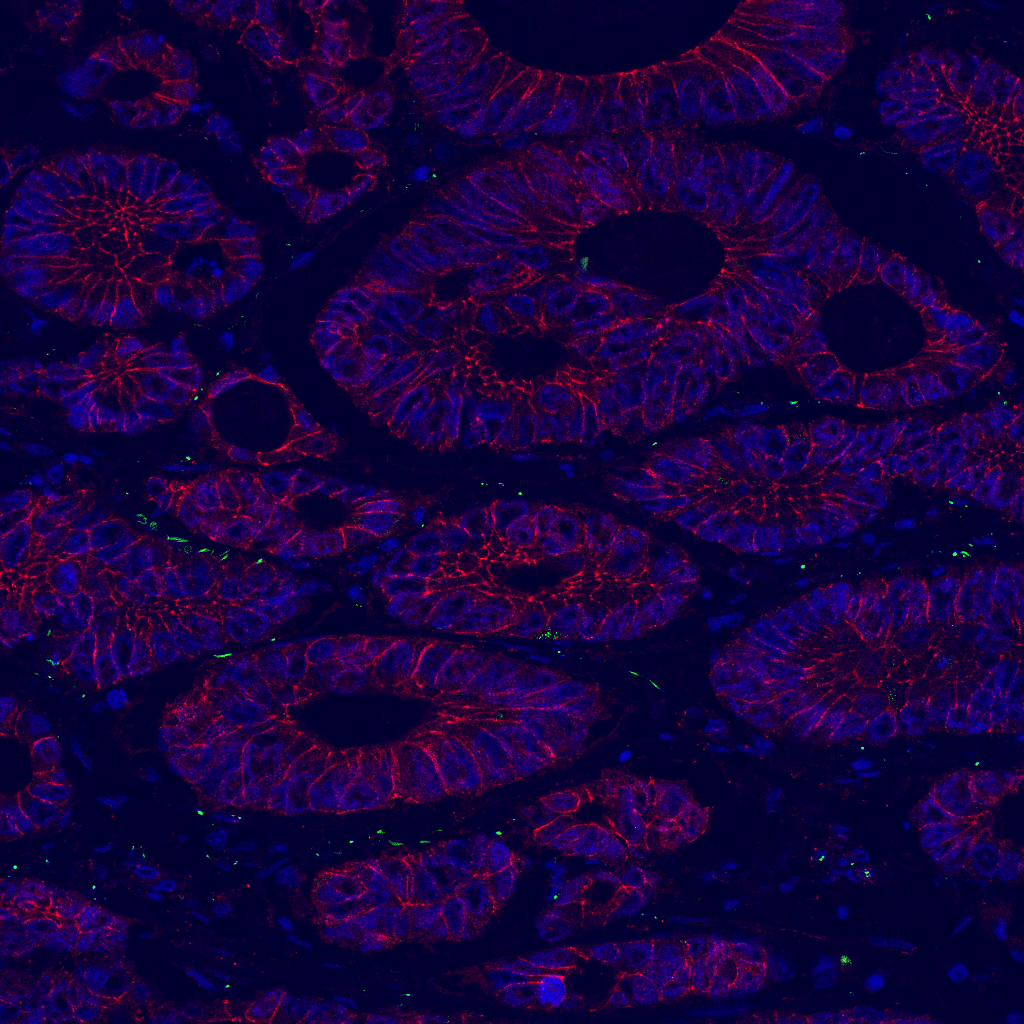

Supplement: Supplementary file 4 — Source data Fig. 2 [file 44321_2024_121_MOESM4_ESM.zip › Figure 2/Figure 2A/Sample 2 dysplastic.tif]

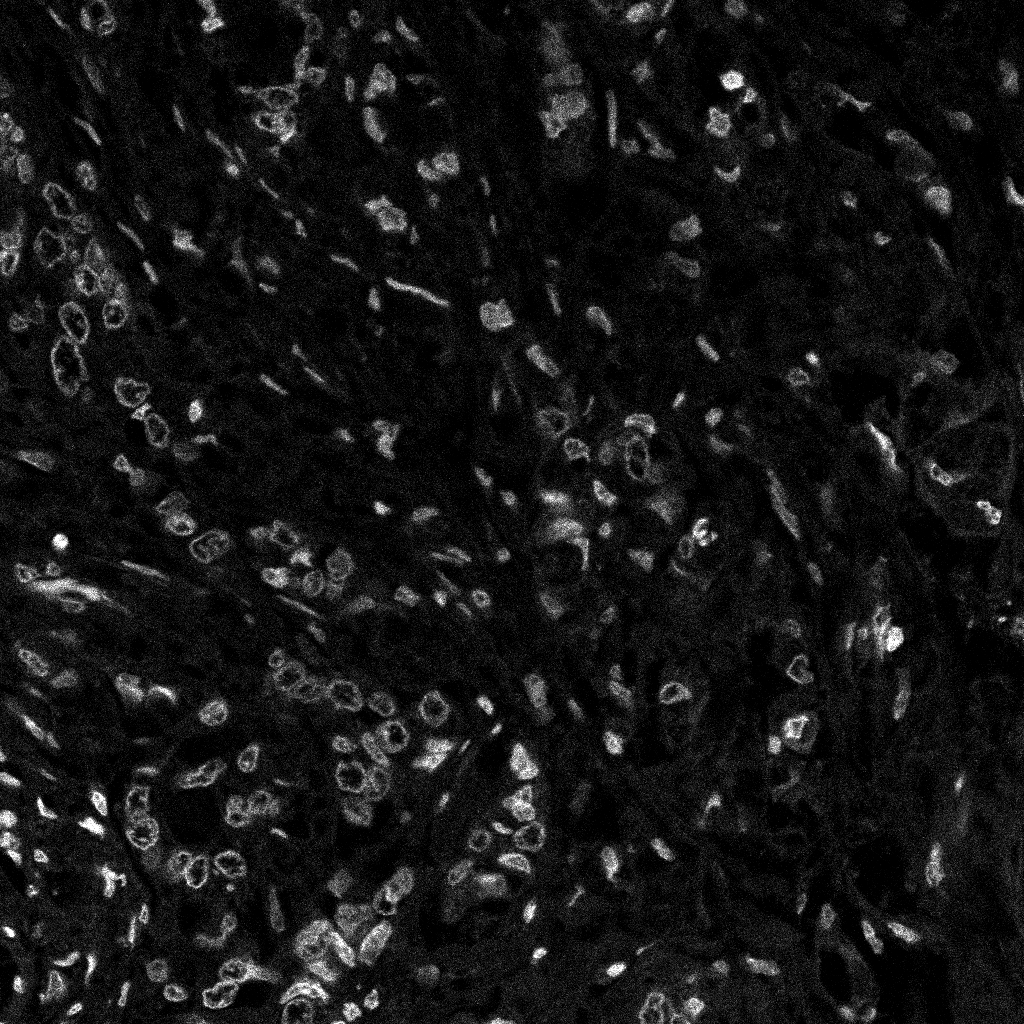

Supplement: Supplementary file 4 — Source data Fig. 2 [file 44321_2024_121_MOESM4_ESM.zip › Figure 2/Figure 2A/Sample 3 dysplastic.tif]

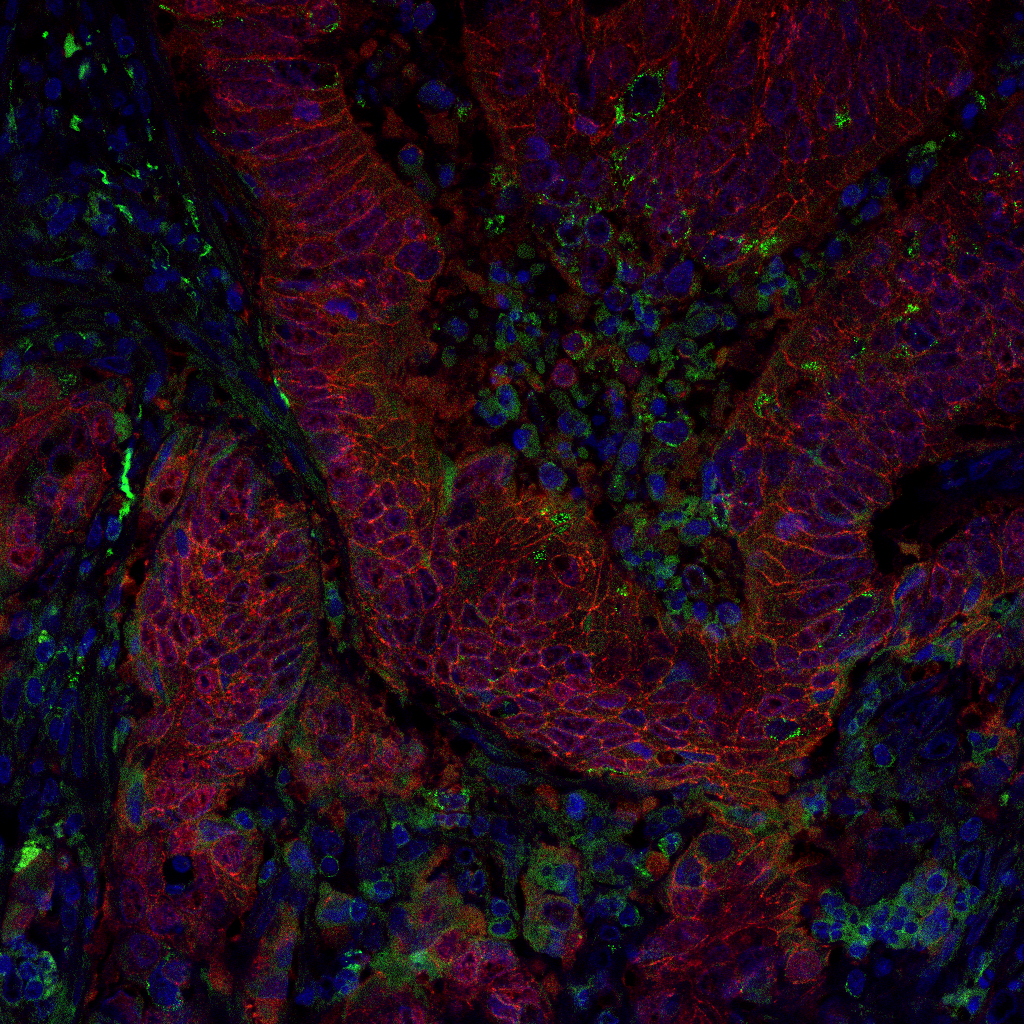

Supplement: Supplementary file 4 — Source data Fig. 2 [file 44321_2024_121_MOESM4_ESM.zip › Figure 2/Figure 2A/Sample 2 cancer.tif]

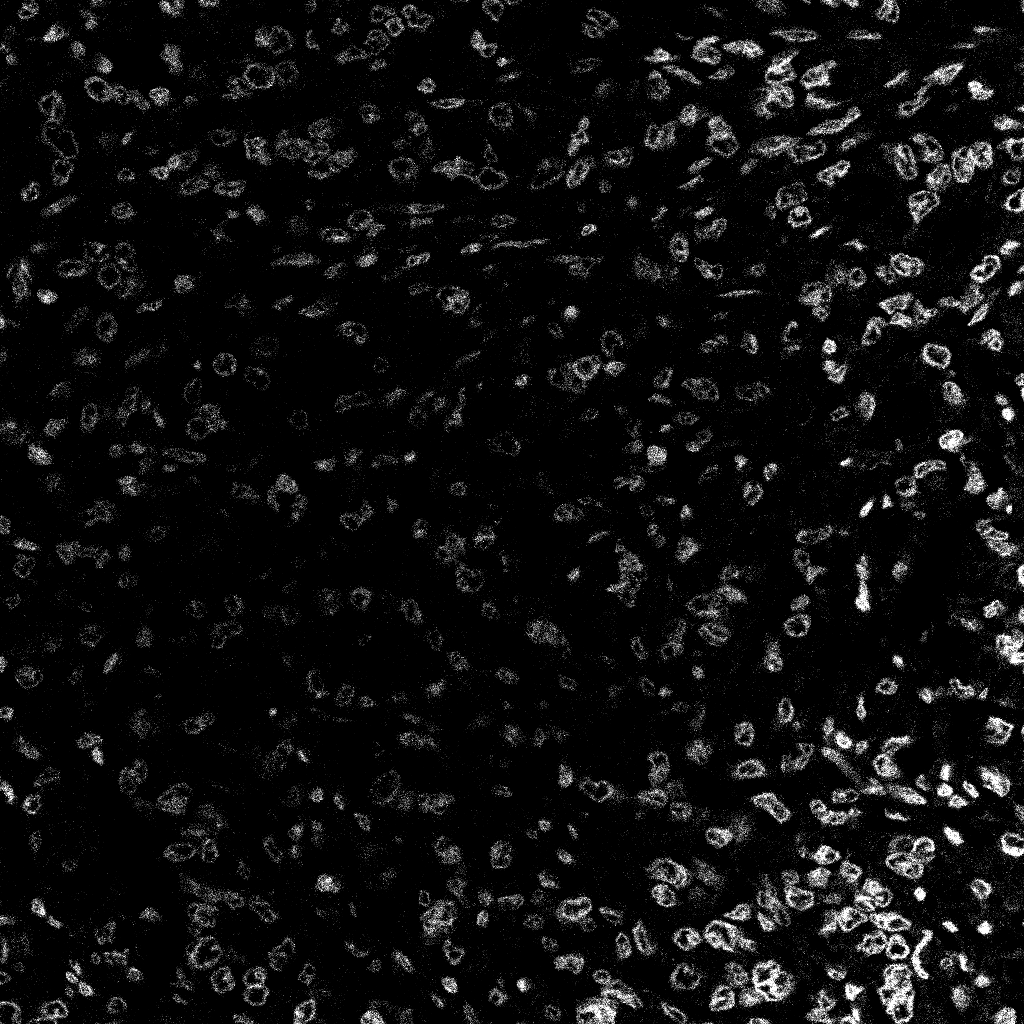

Supplement: Supplementary file 4 — Source data Fig. 2 [file 44321_2024_121_MOESM4_ESM.zip › Figure 2/Figure 2A/Sample 1 cancer.tif]

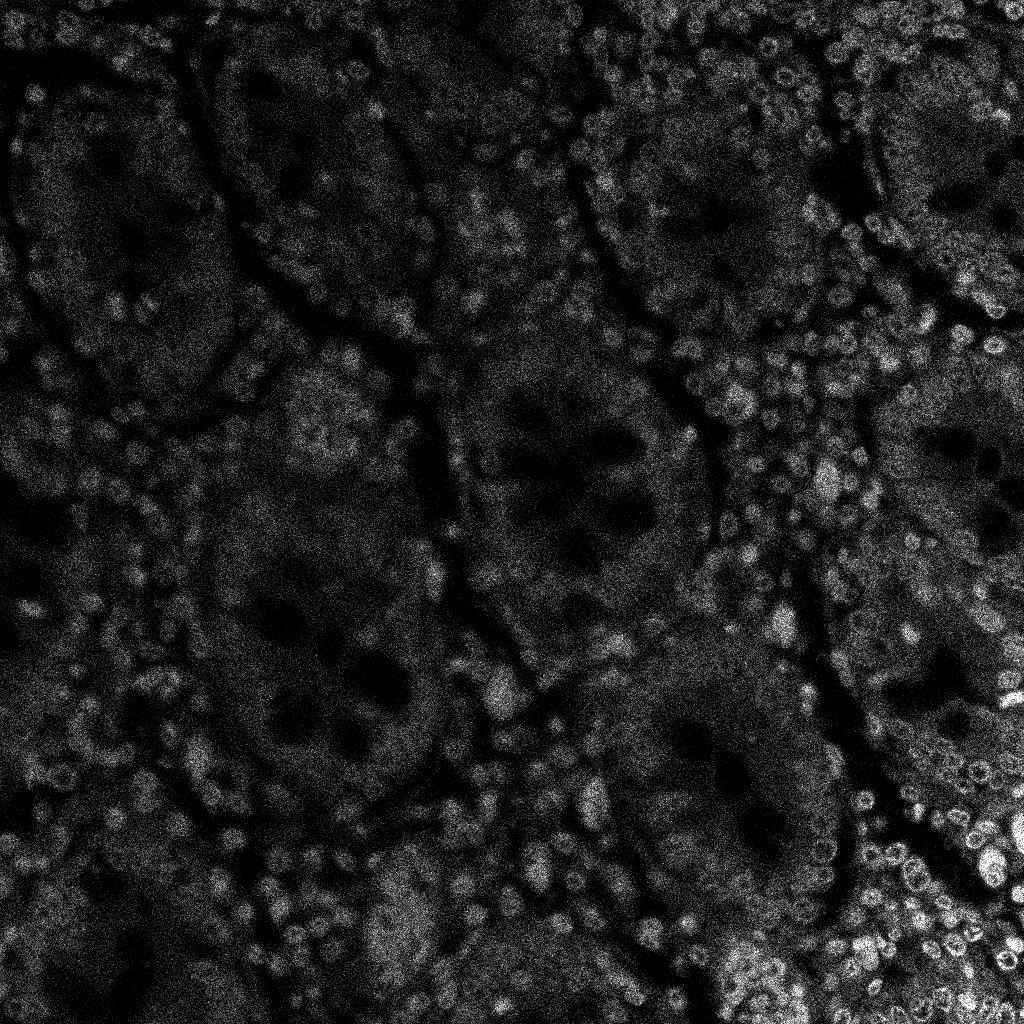

Supplement: Supplementary file 4 — Source data Fig. 2 [file 44321_2024_121_MOESM4_ESM.zip › Figure 2/Figure 2A/Sample 1 normal.tif]

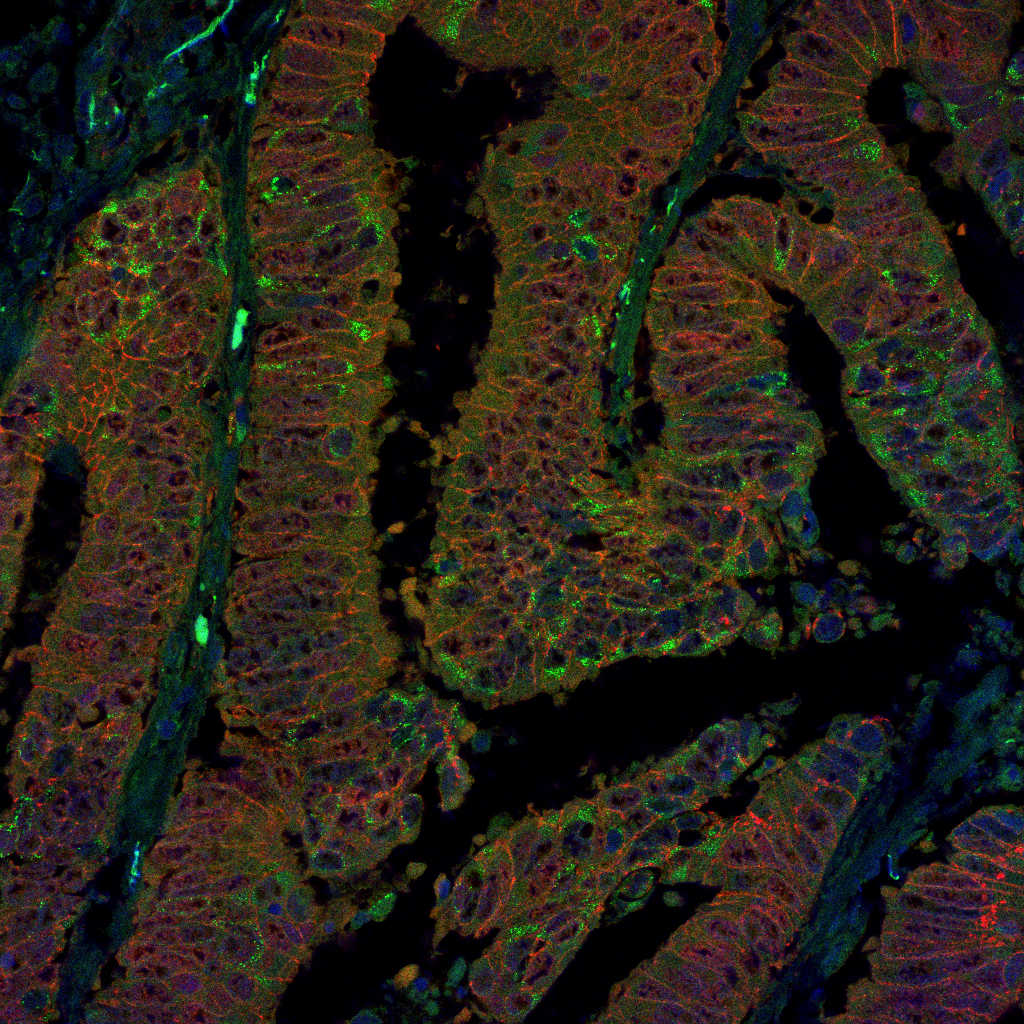

Supplement: Supplementary file 4 — Source data Fig. 2 [file 44321_2024_121_MOESM4_ESM.zip › Figure 2/Figure 2A/Sample 3 cancer.tif]

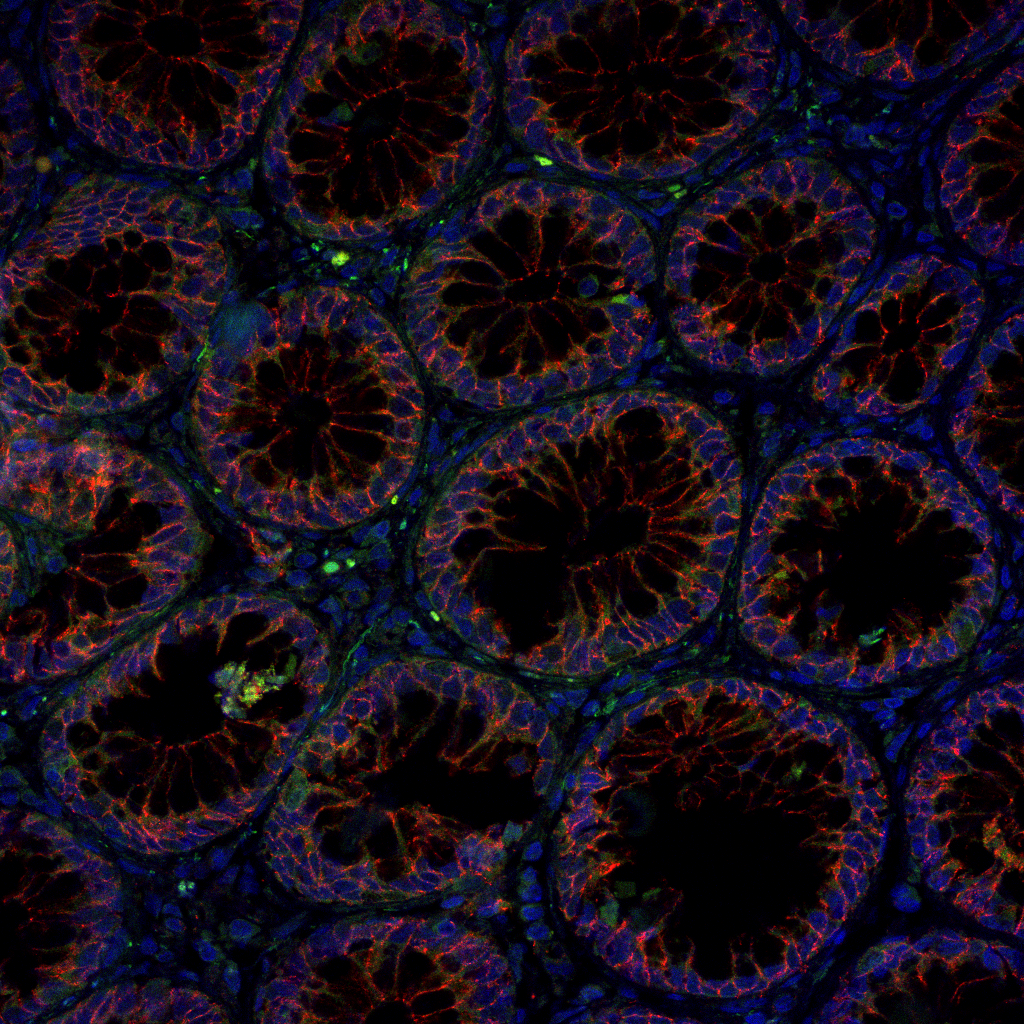

Supplement: Supplementary file 4 — Source data Fig. 2 [file 44321_2024_121_MOESM4_ESM.zip › Figure 2/Figure 2A/Sample 3 normal.tif]

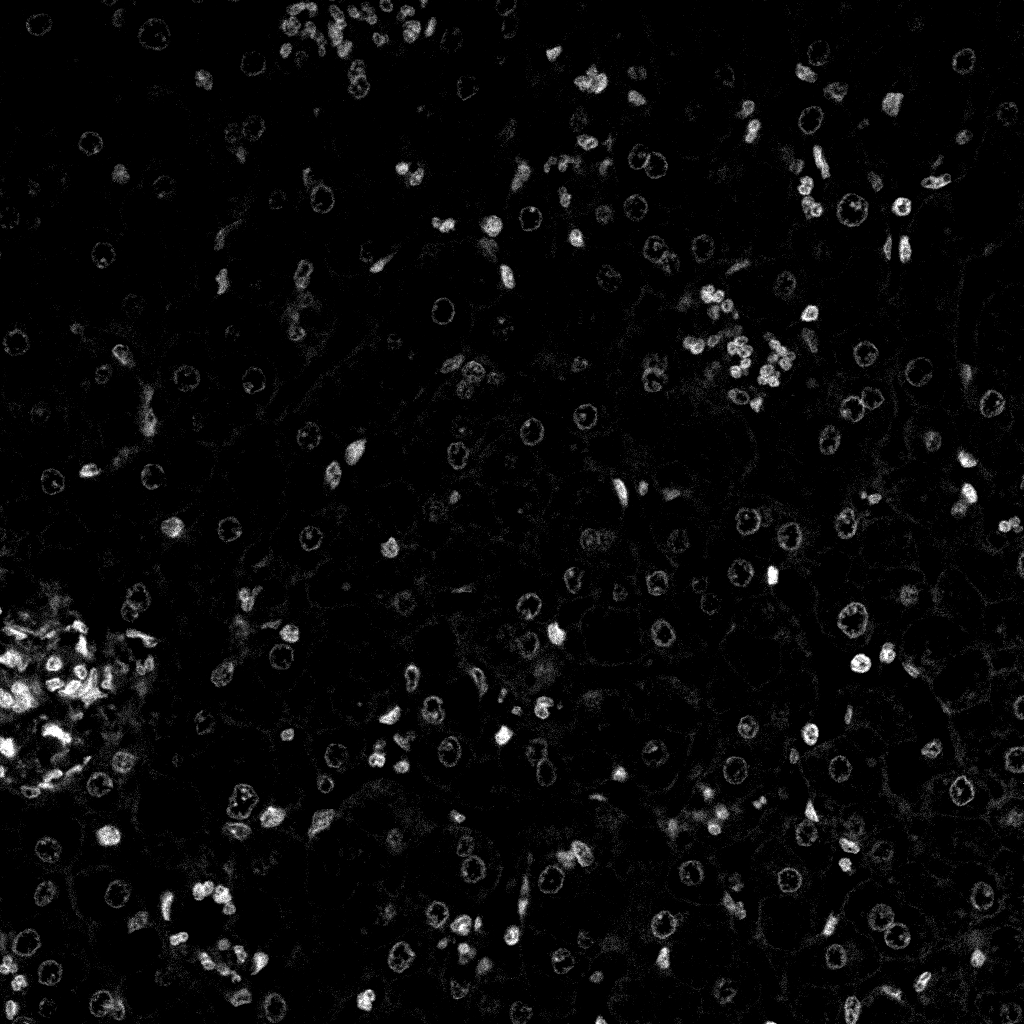

Supplement: Supplementary file 4 — Source data Fig. 2 [file 44321_2024_121_MOESM4_ESM.zip › Figure 2/Figure 2C/healthy liver.tif]

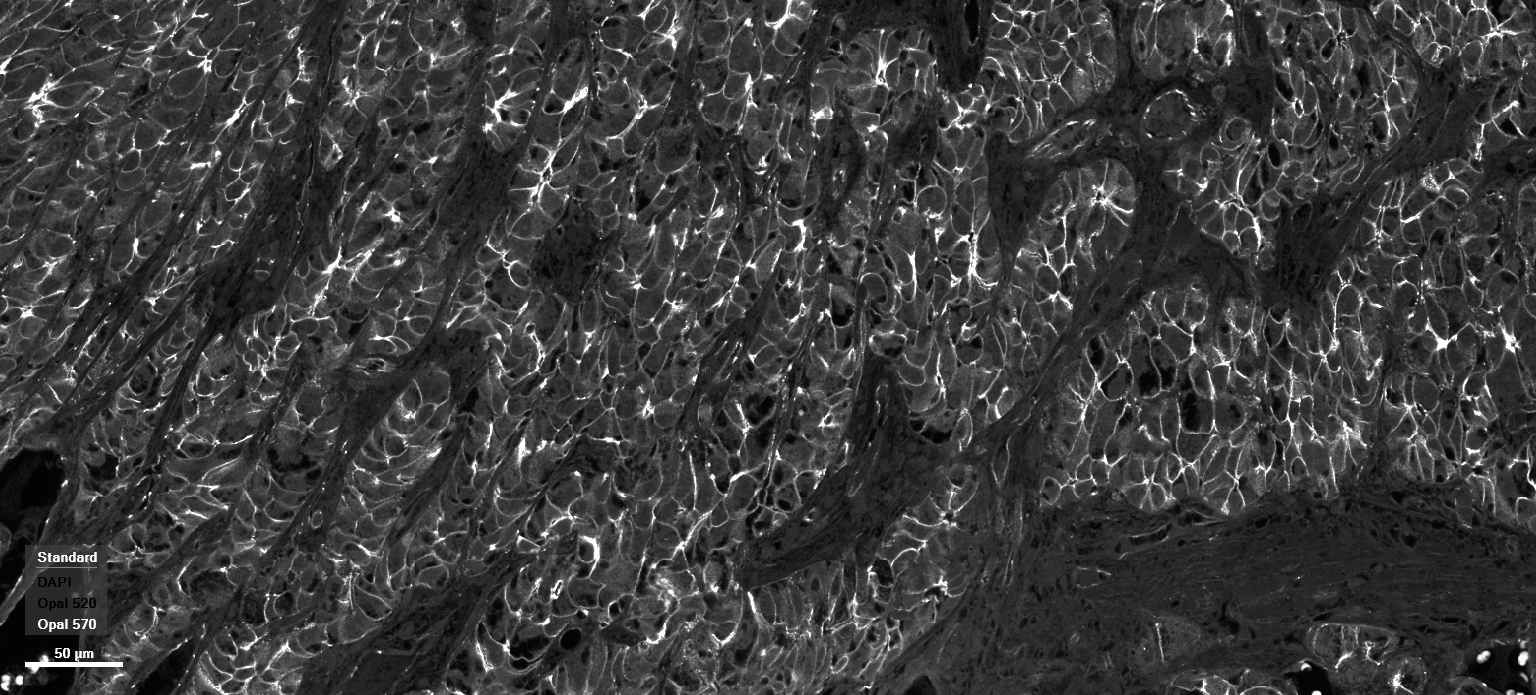

Supplement: Supplementary file 4 — Source data Fig. 2 [file 44321_2024_121_MOESM4_ESM.zip › Figure 2/Figure 2C/HCC.tif]

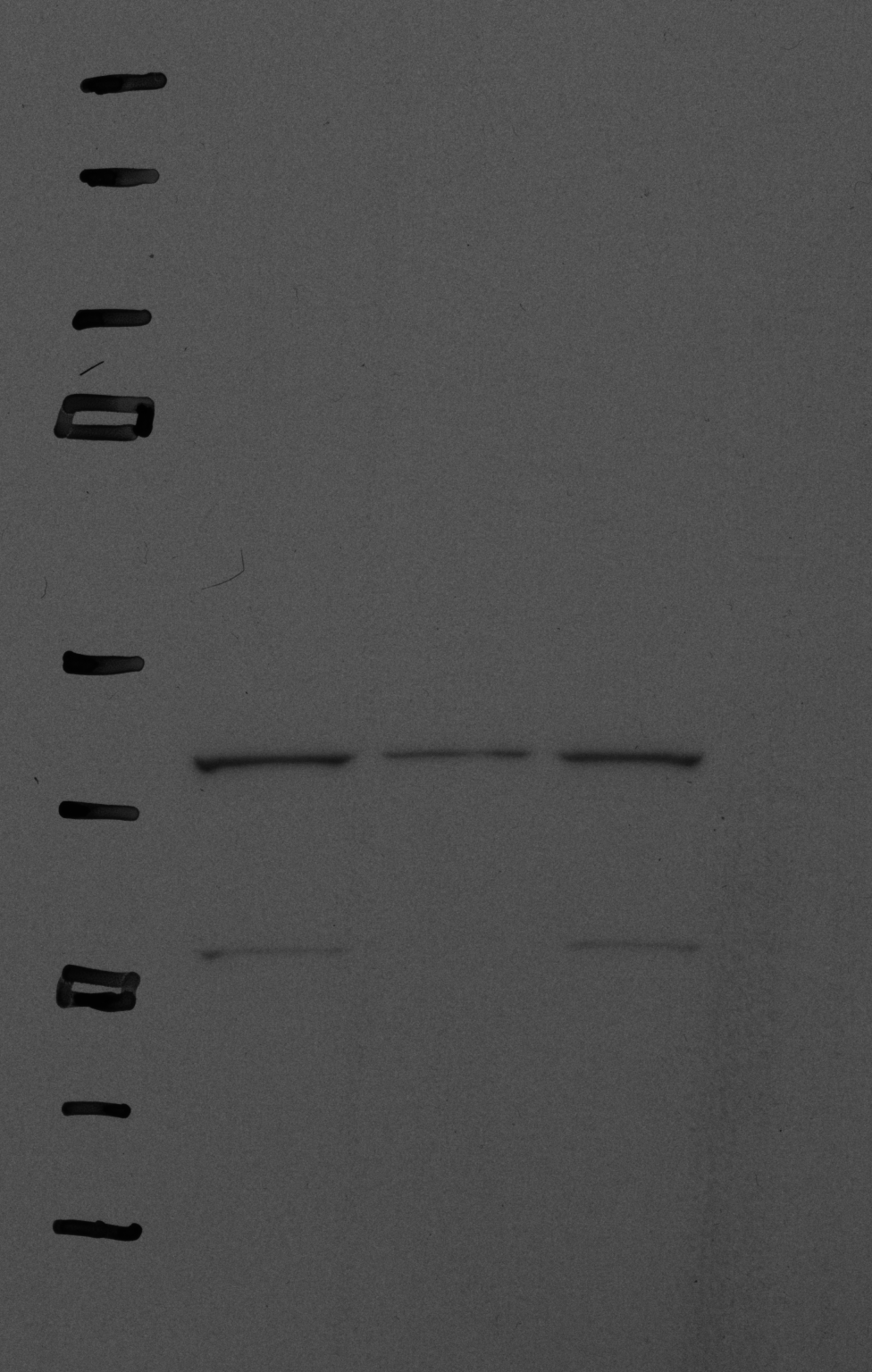

Supplement: Supplementary file 5 — Source data Fig. 3 [file 44321_2024_121_MOESM5_ESM.zip › Figure 3/Figure 3K/Figure_3K_140423_HepG2_Hep3B_PLC_PRF5_actin_30sec.tif]

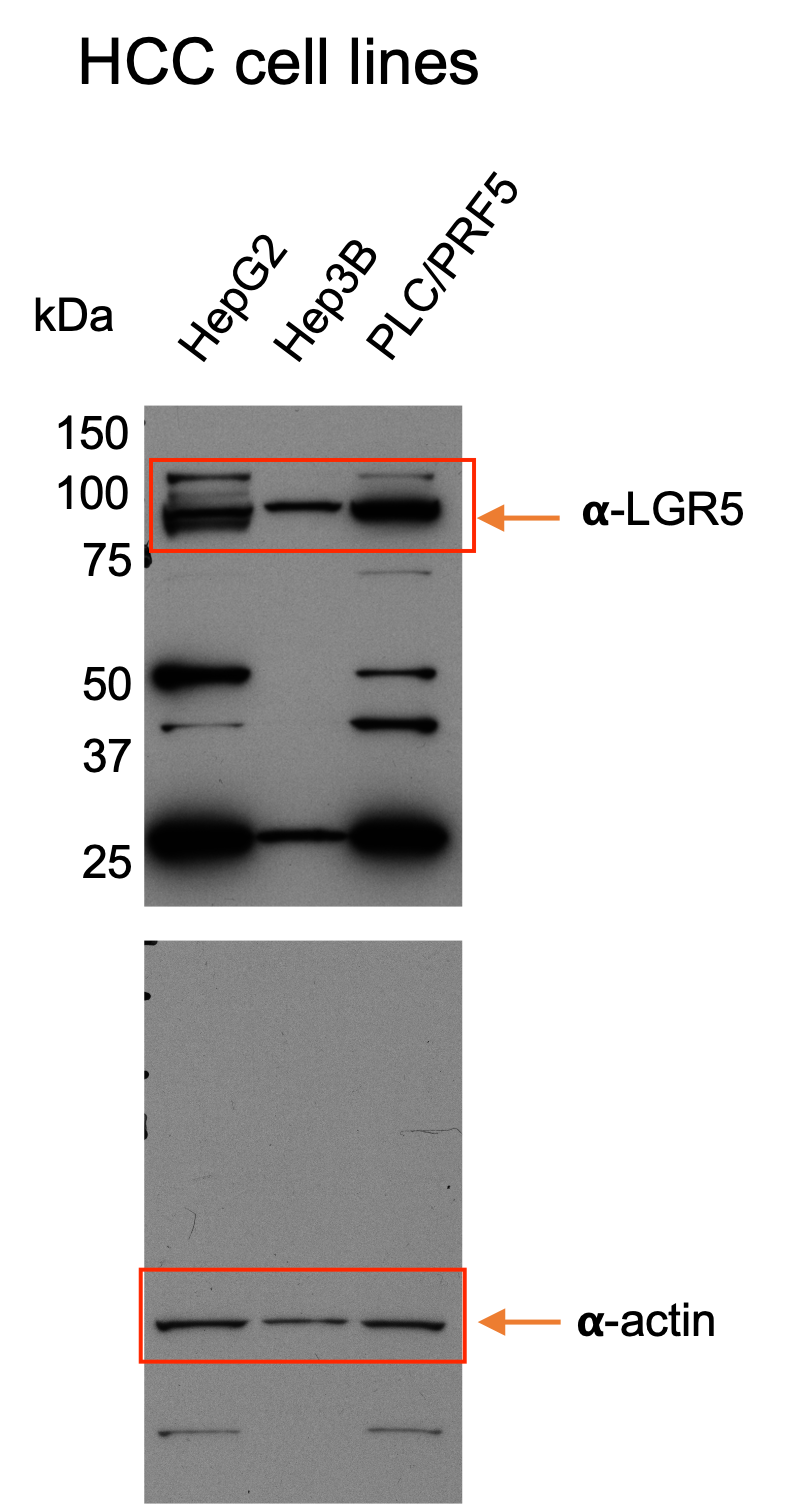

Supplement: Supplementary file 5 — Source data Fig. 3 [file 44321_2024_121_MOESM5_ESM.zip › Figure 3/Figure 3K/Figure_3K_western_blot_HCC_LGR5_profiling.png]

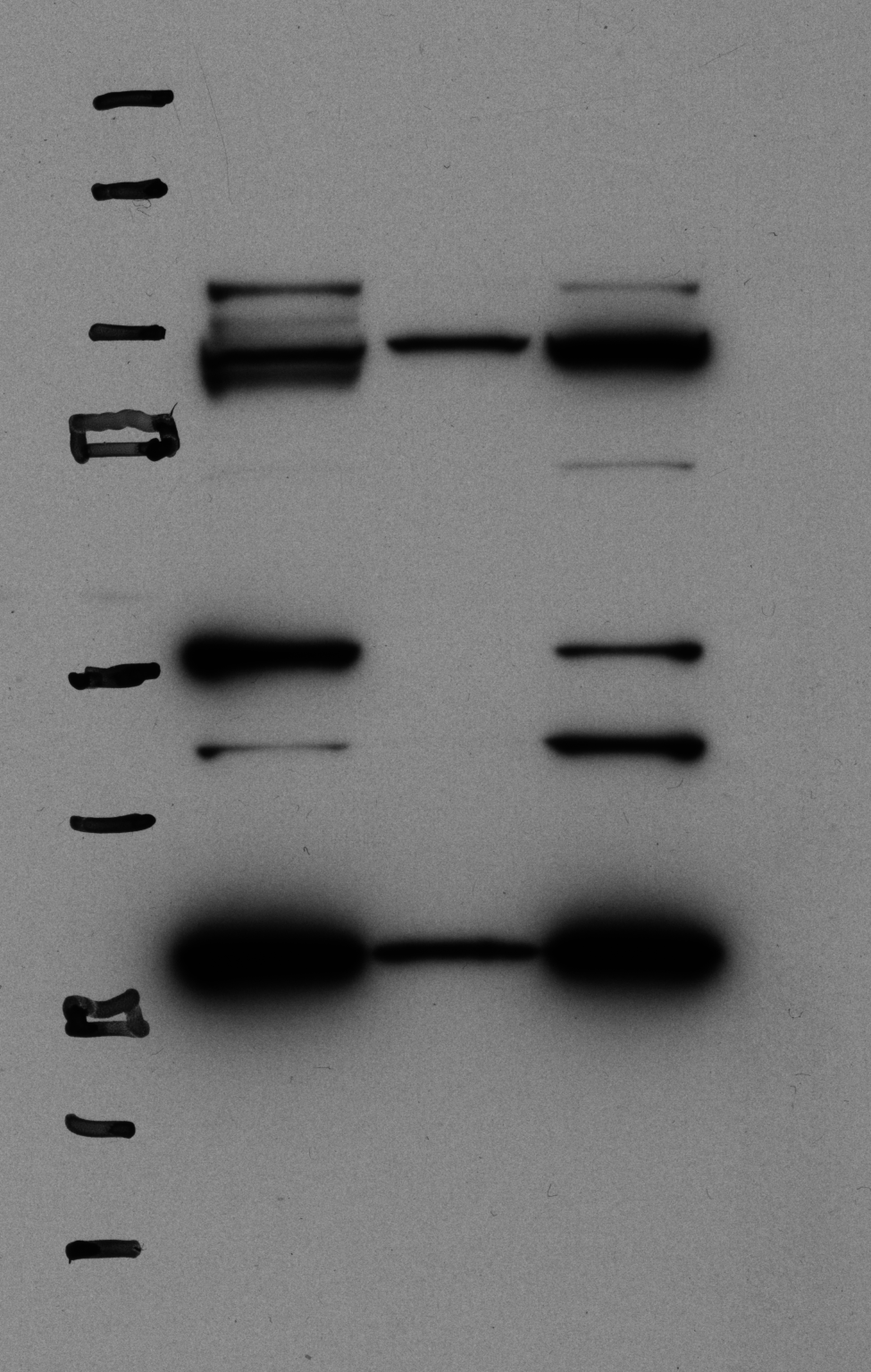

Supplement: Supplementary file 5 — Source data Fig. 3 [file 44321_2024_121_MOESM5_ESM.zip › Figure 3/Figure 3K/Figure_3K_140423_HepG2_Hep3B_PLC_PRF5_LGR5_5min.tif]

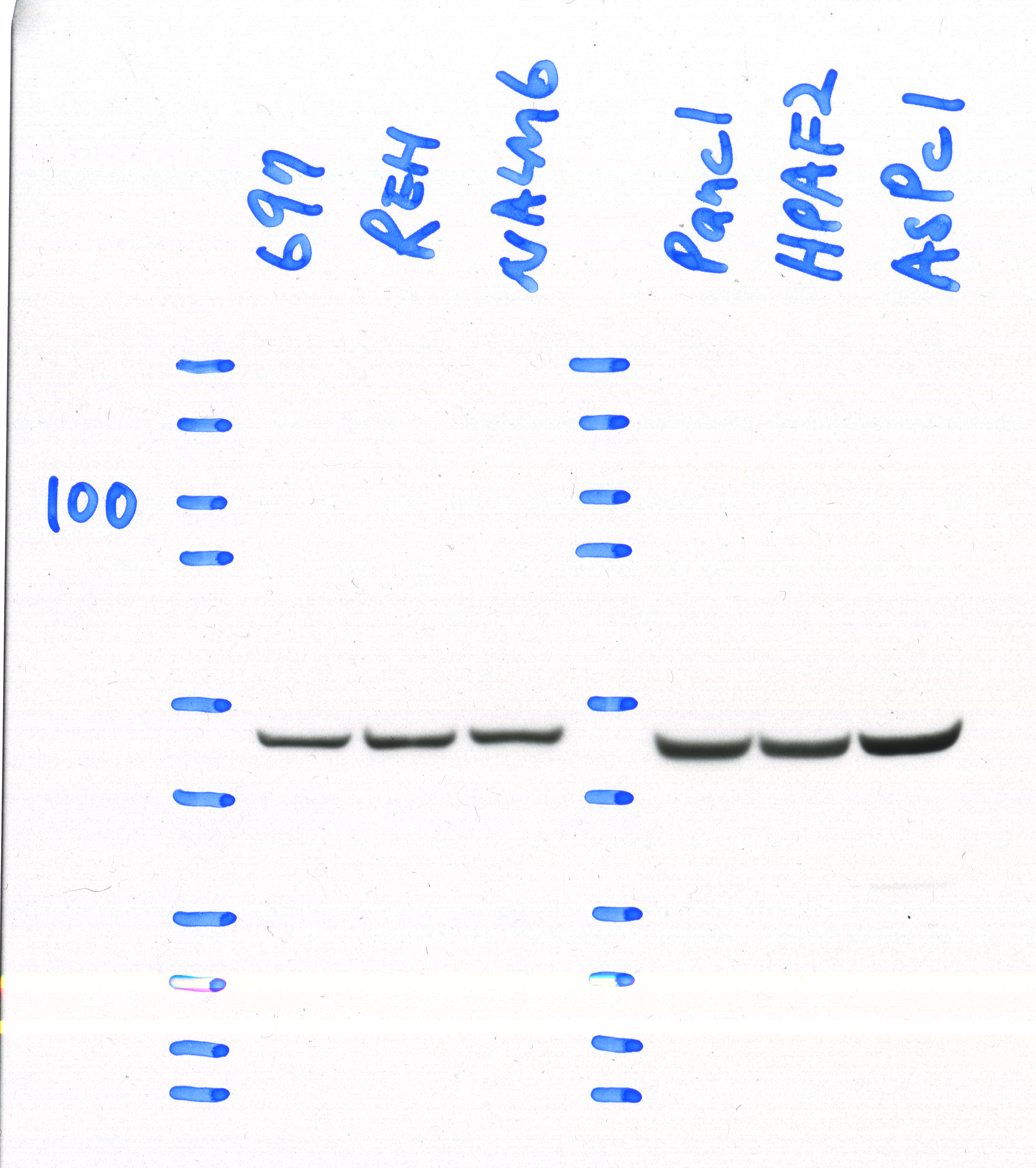

Supplement: Supplementary file 5 — Source data Fig. 3 [file 44321_2024_121_MOESM5_ESM.zip › Figure 3/Figure 3B/Figure3B_LGR5_expression_cell_lines_tubulin.tif]

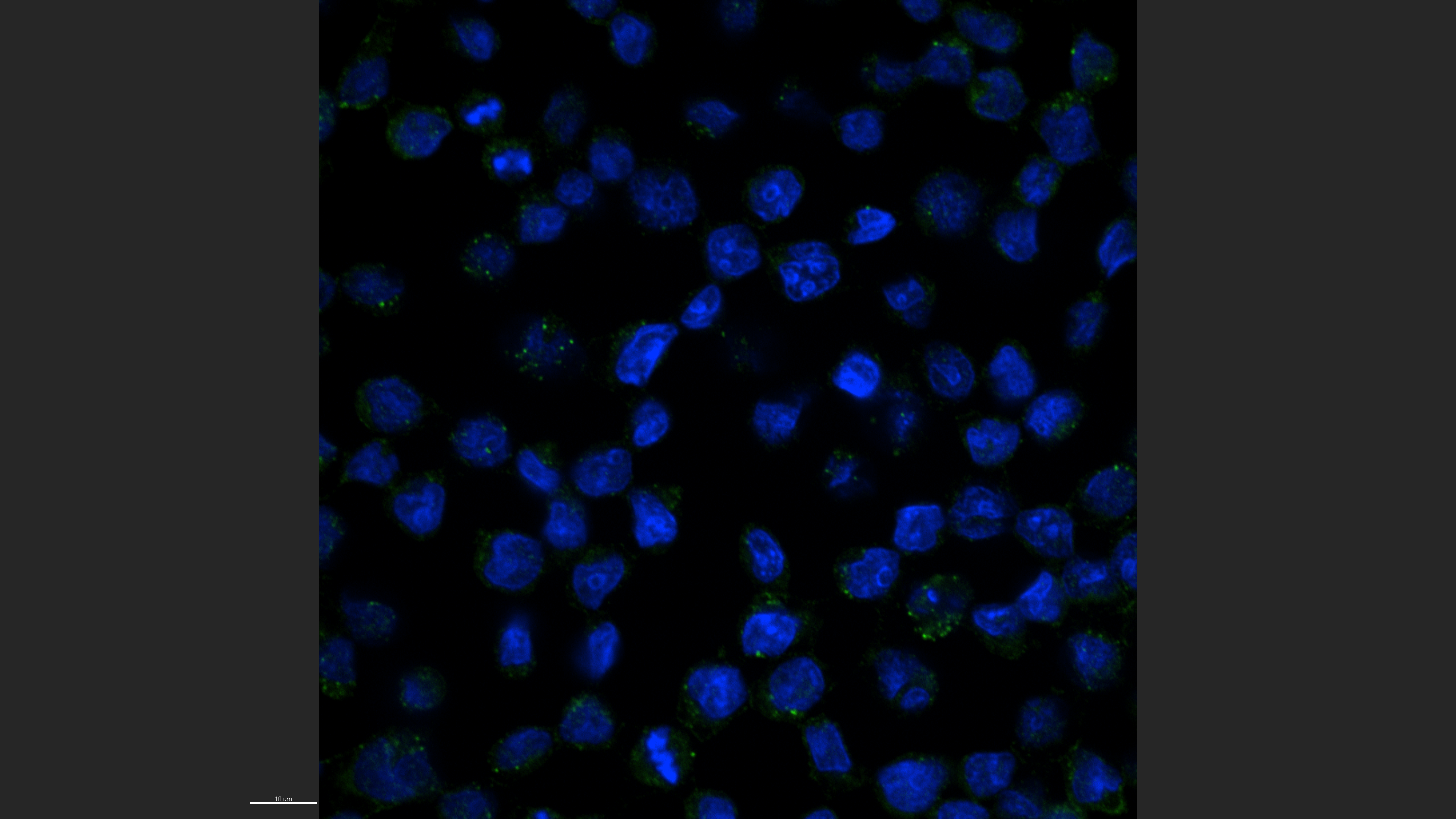

Supplement: Supplementary file 5 — Source data Fig. 3 [file 44321_2024_121_MOESM5_ESM.zip › Figure 3/Figure 3C/NALM6 all.tif]

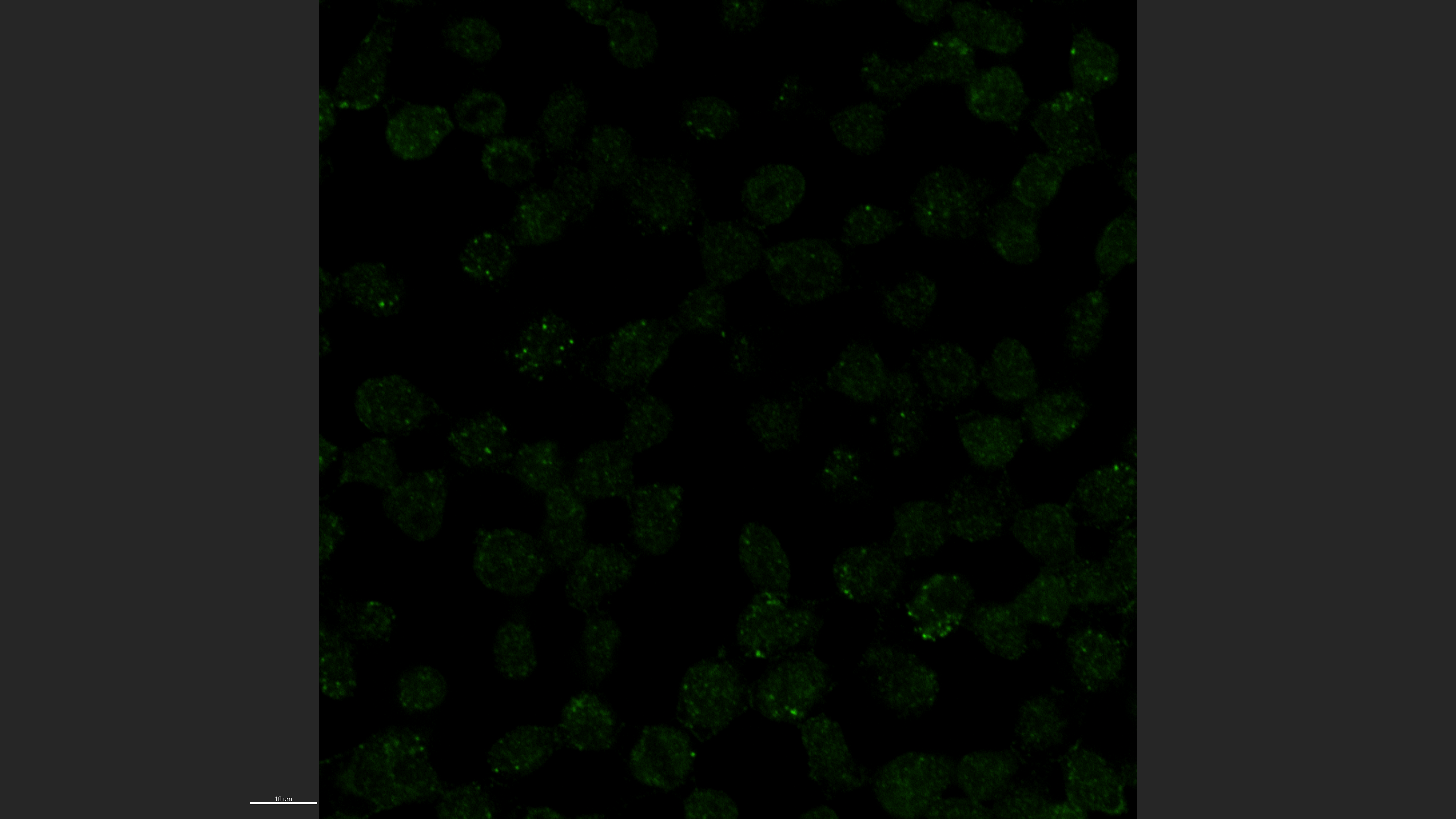

Supplement: Supplementary file 5 — Source data Fig. 3 [file 44321_2024_121_MOESM5_ESM.zip › Figure 3/Figure 3C/NALM6_green channel only.tif]

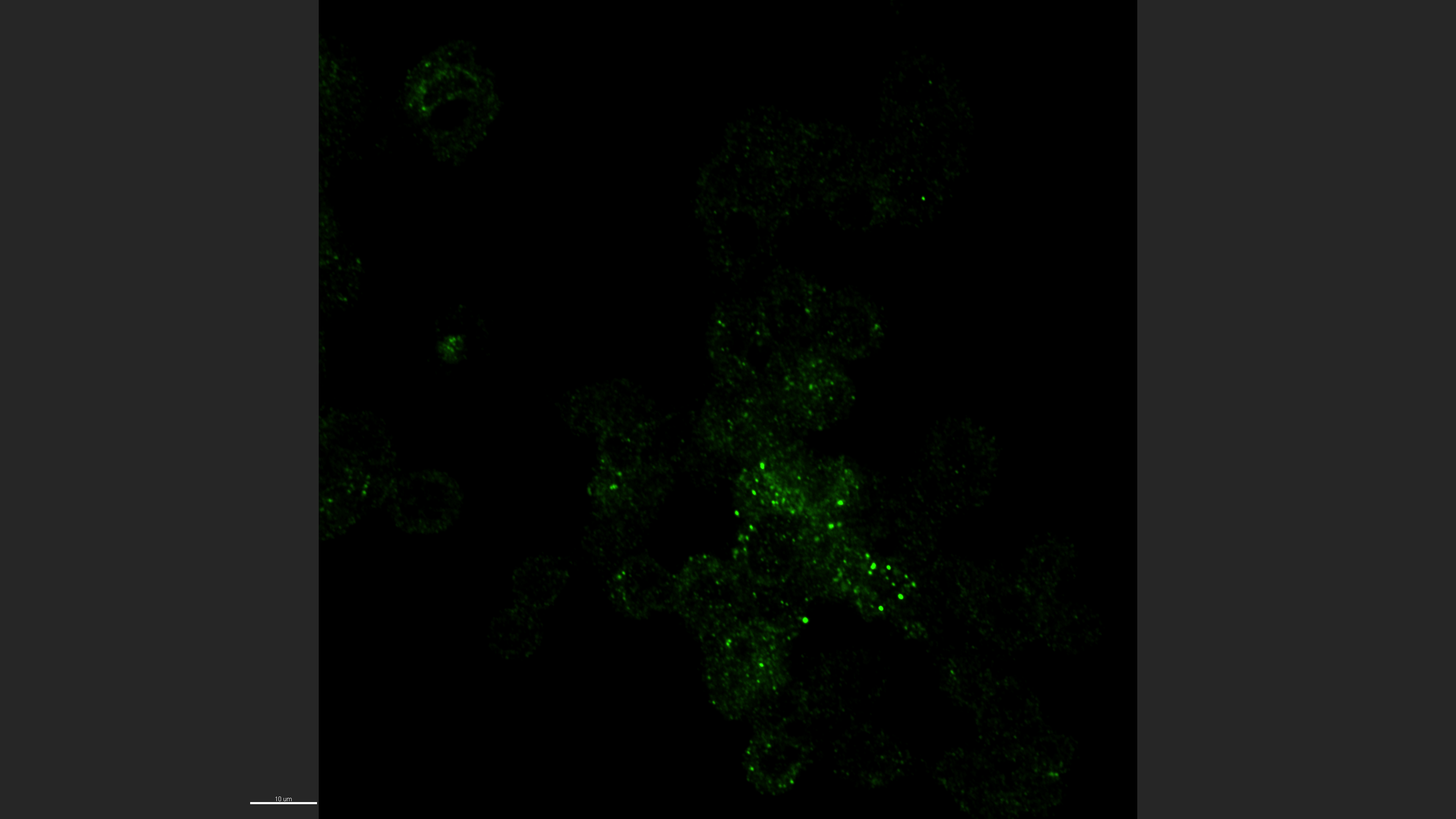

Supplement: Supplementary file 5 — Source data Fig. 3 [file 44321_2024_121_MOESM5_ESM.zip › Figure 3/Figure 3H/LoVo green channel onlytif.tif]

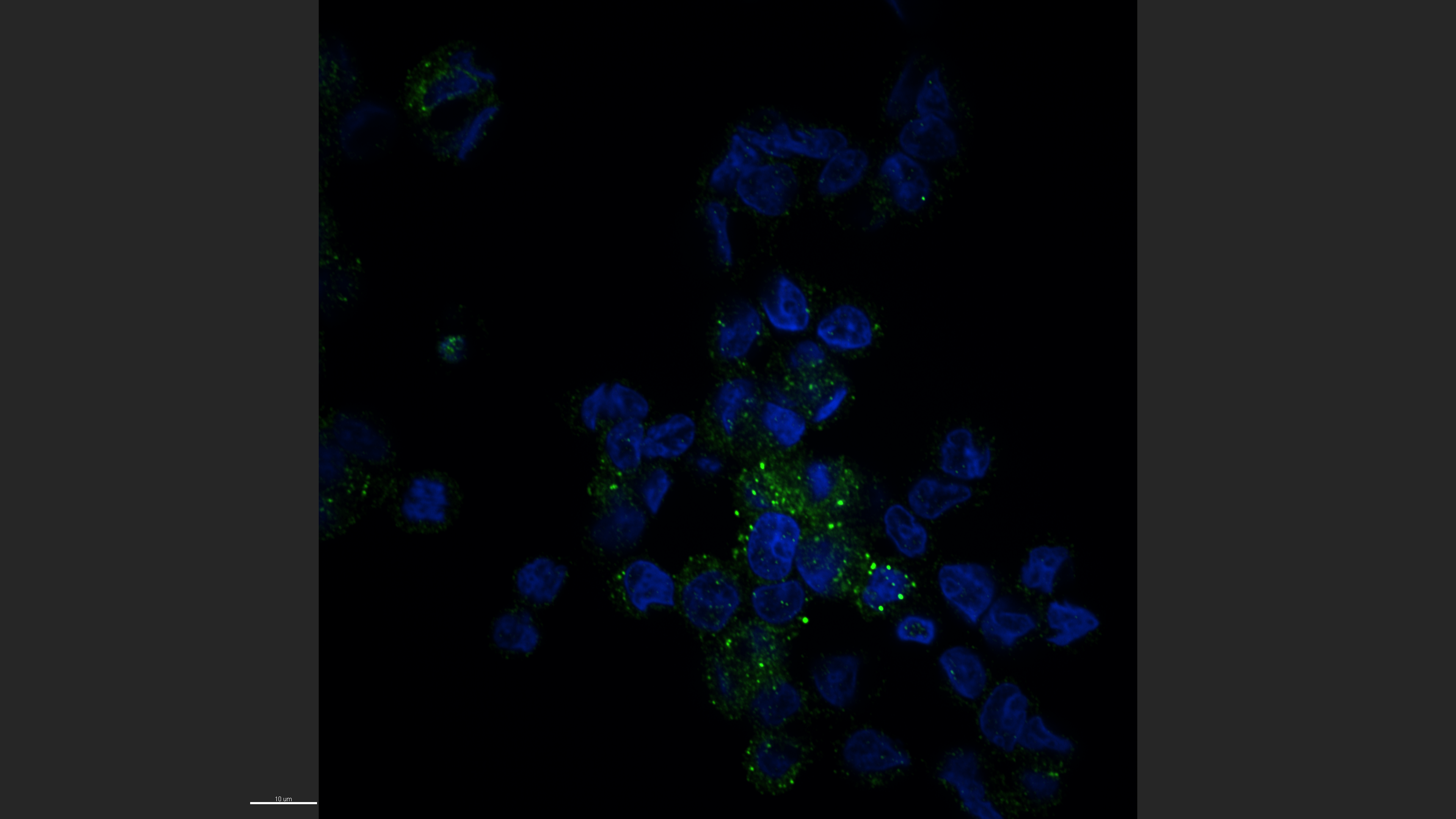

Supplement: Supplementary file 5 — Source data Fig. 3 [file 44321_2024_121_MOESM5_ESM.zip › Figure 3/Figure 3H/LoVo all.tif]

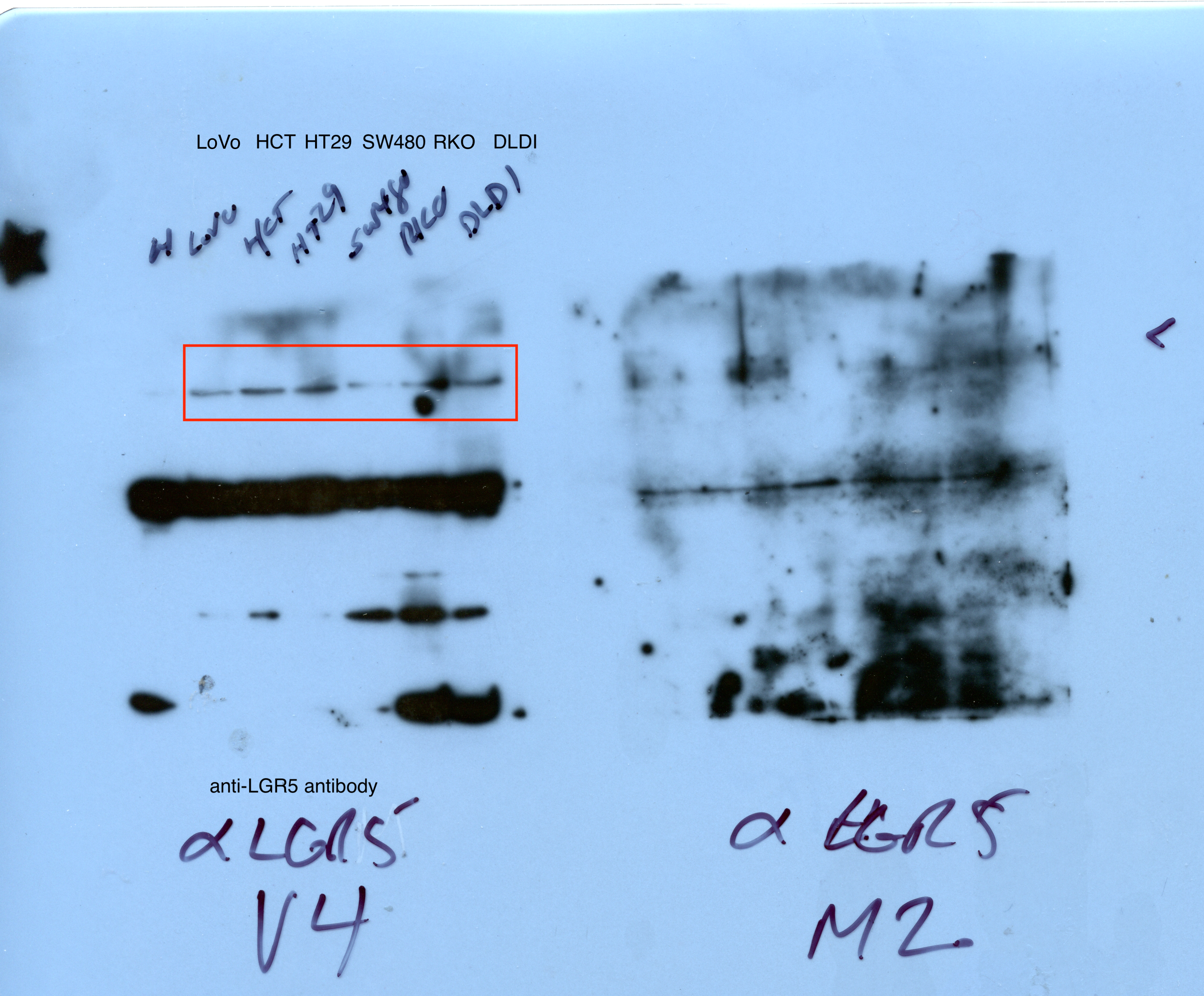

Supplement: Supplementary file 5 — Source data Fig. 3 [file 44321_2024_121_MOESM5_ESM.zip › Figure 3/Figure 3G/Figure_3G_Western_Blot_anti_LGR5_CRC_cell_lines.tif]

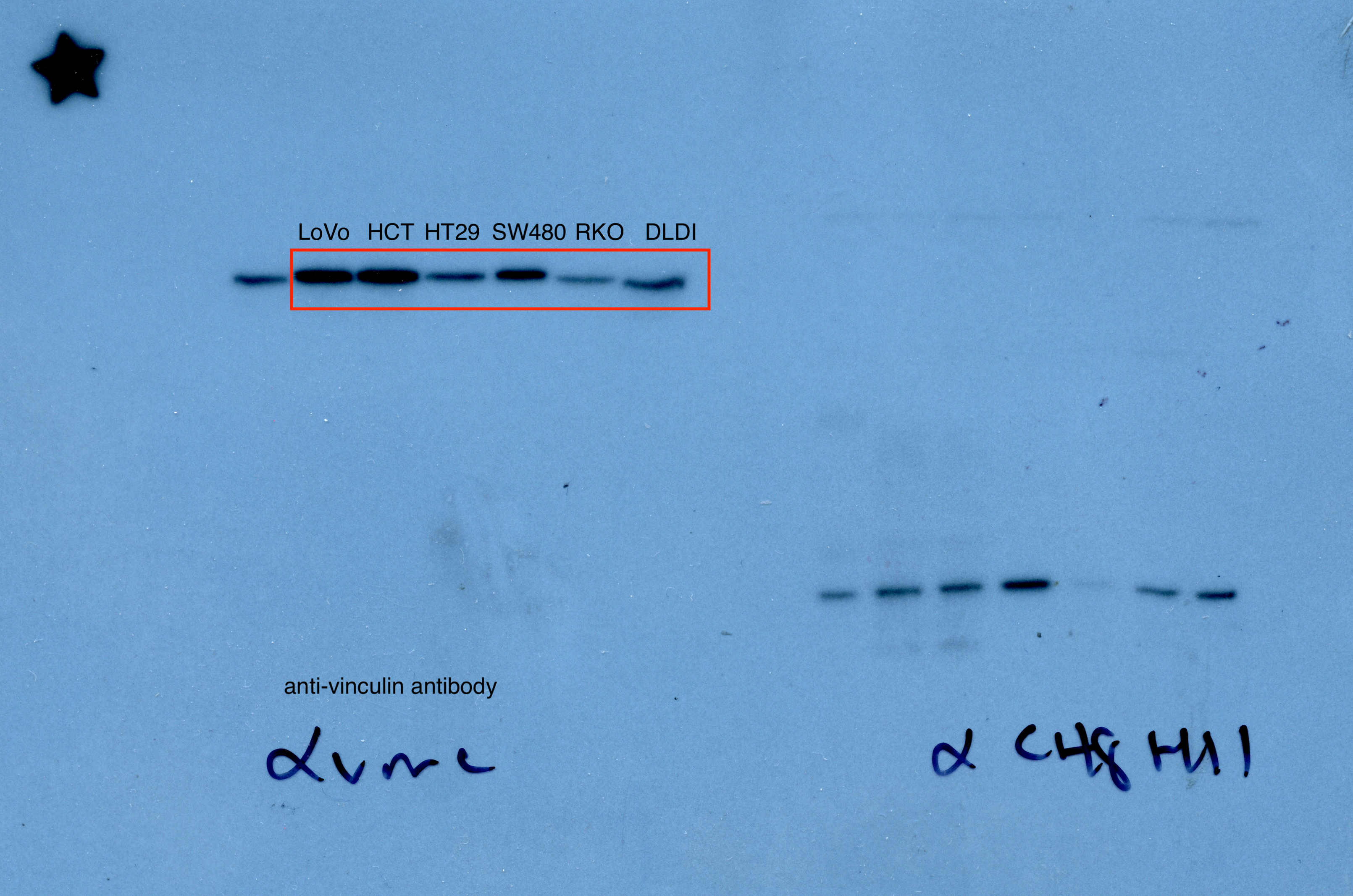

Supplement: Supplementary file 5 — Source data Fig. 3 [file 44321_2024_121_MOESM5_ESM.zip › Figure 3/Figure 3G/Figure_3G_Western_Blot_anti_vinculin_CRC_cell_lines.tif]

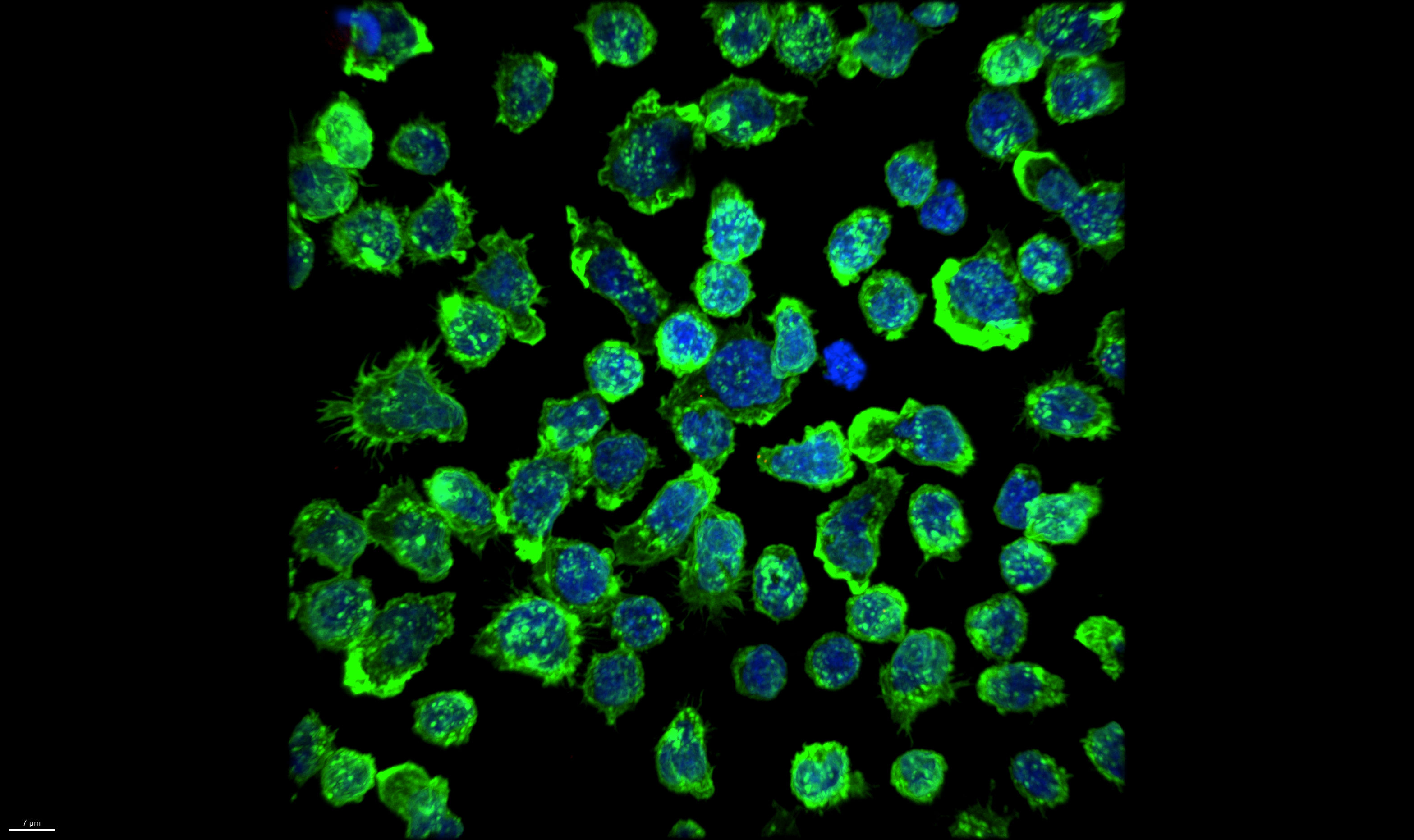

Supplement: Supplementary file 6 — Source data Fig. 4 [file 44321_2024_121_MOESM6_ESM.zip › Figure 4/Figure 4B/20210908 LGR5 internalisation_NALM6_60 mins_mIgG2b_3_merge.tif]

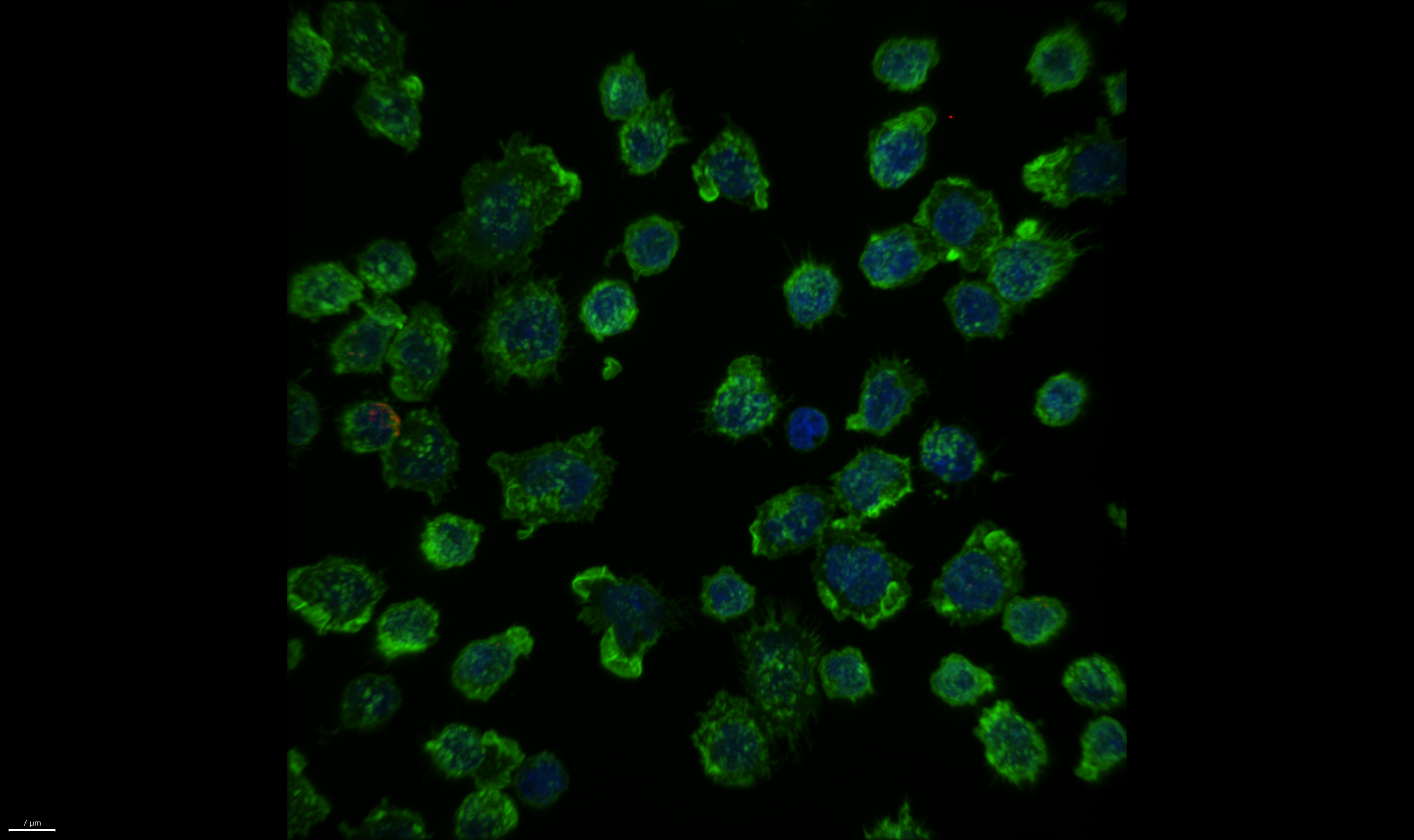

Supplement: Supplementary file 6 — Source data Fig. 4 [file 44321_2024_121_MOESM6_ESM.zip › Figure 4/Figure 4B/20210908 LGR5 internalisation_NALM6_5 mins_mc2_4_merge.tif]

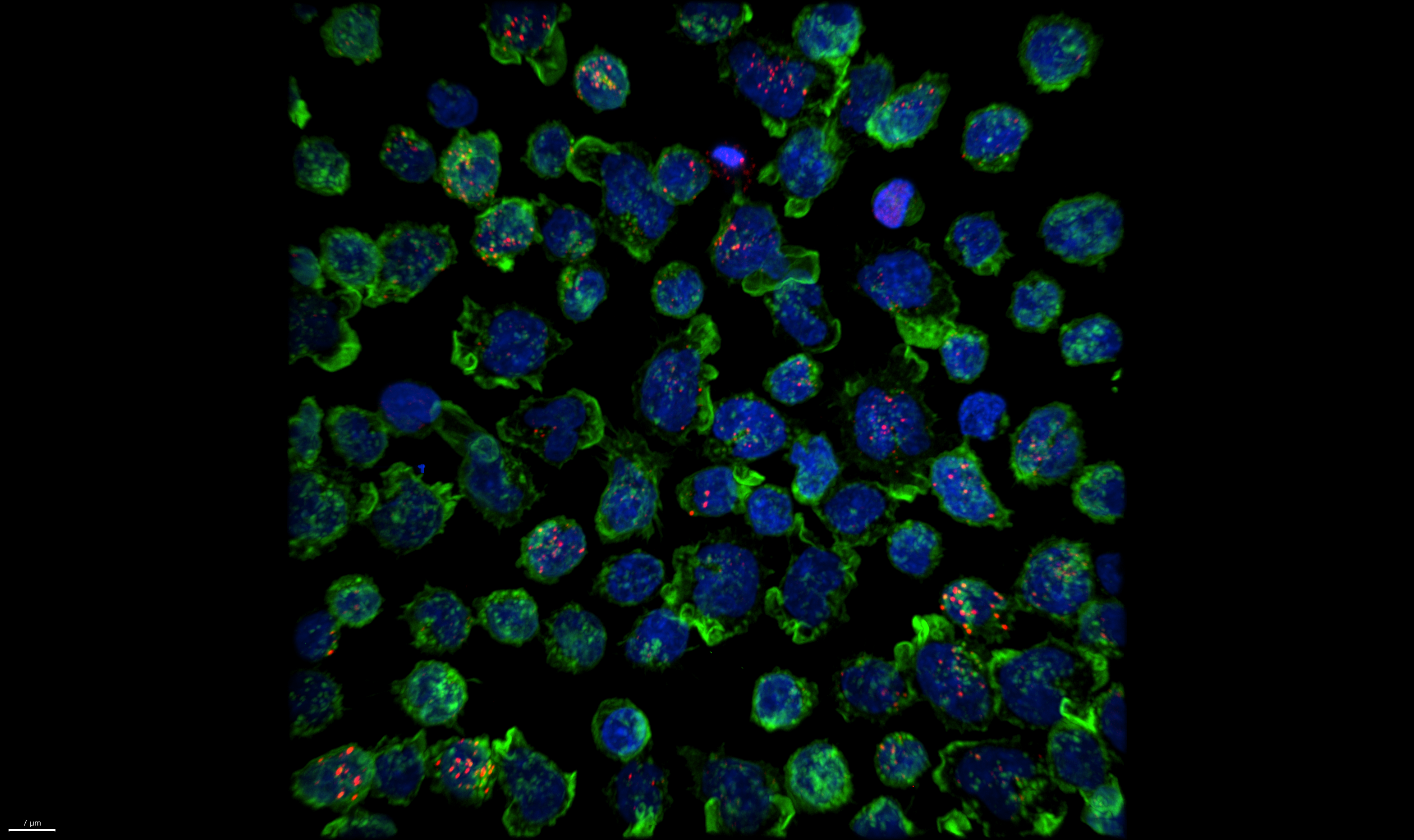

Supplement: Supplementary file 6 — Source data Fig. 4 [file 44321_2024_121_MOESM6_ESM.zip › Figure 4/Figure 4B/20210908 LGR5 internalisation_NALM6_60 mins_mc2_3_merge.tif]

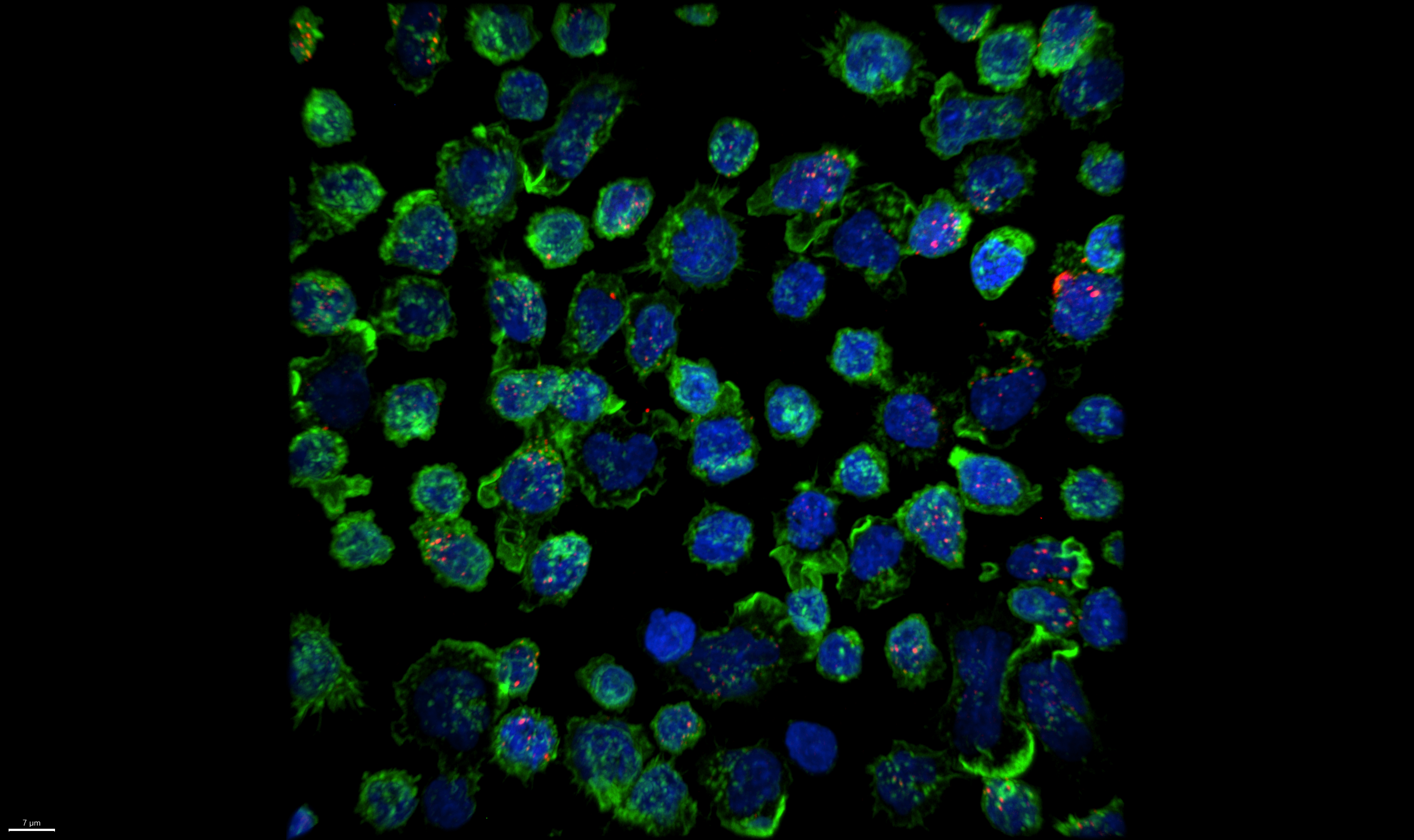

Supplement: Supplementary file 6 — Source data Fig. 4 [file 44321_2024_121_MOESM6_ESM.zip › Figure 4/Figure 4B/20210908 LGR5 internalisation_NALM6_30 mins_mc2_2_merge.tif]

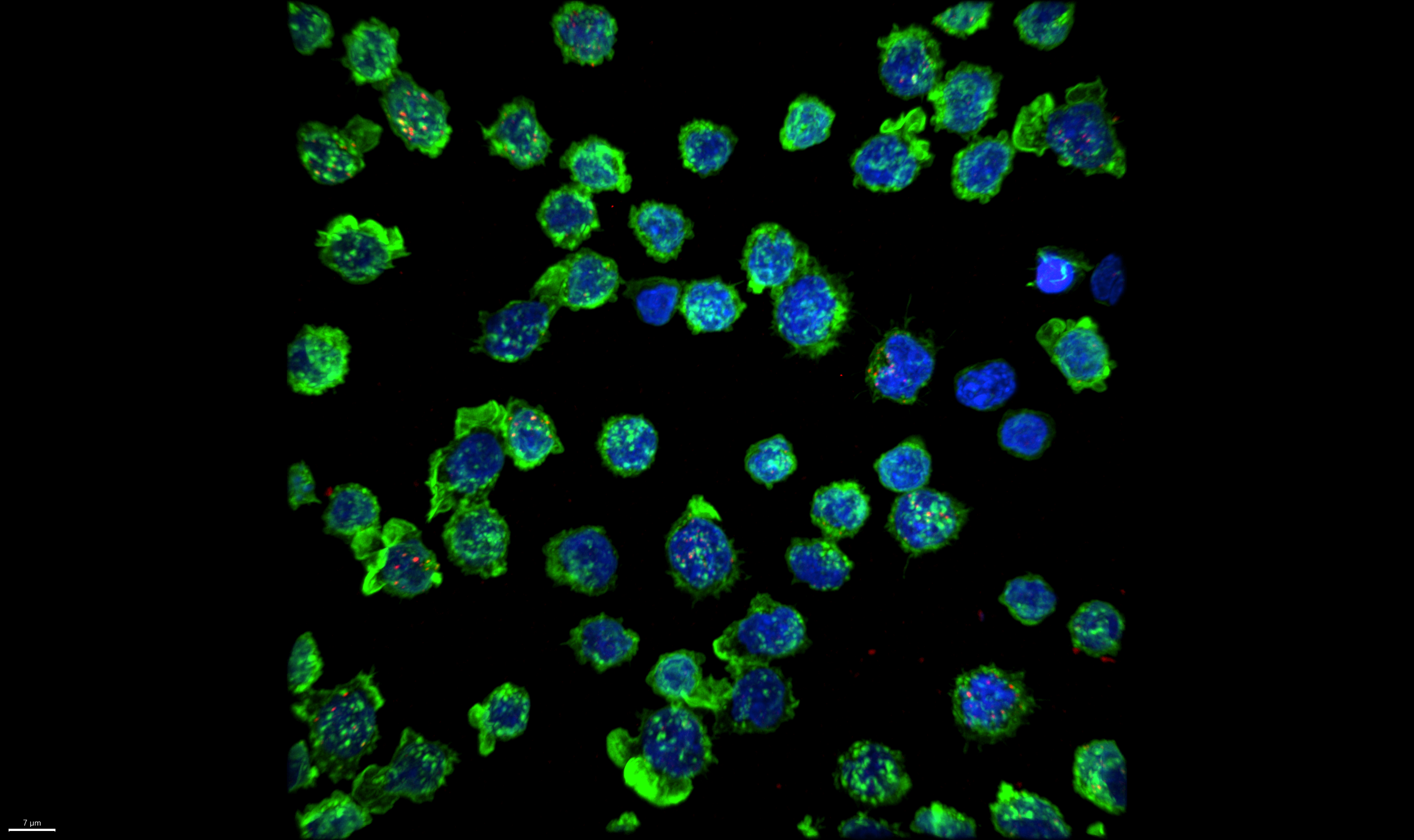

Supplement: Supplementary file 6 — Source data Fig. 4 [file 44321_2024_121_MOESM6_ESM.zip › Figure 4/Figure 4B/20210908 LGR5 internalisation_NALM6_15 mins_mc2_4_merge.tif]

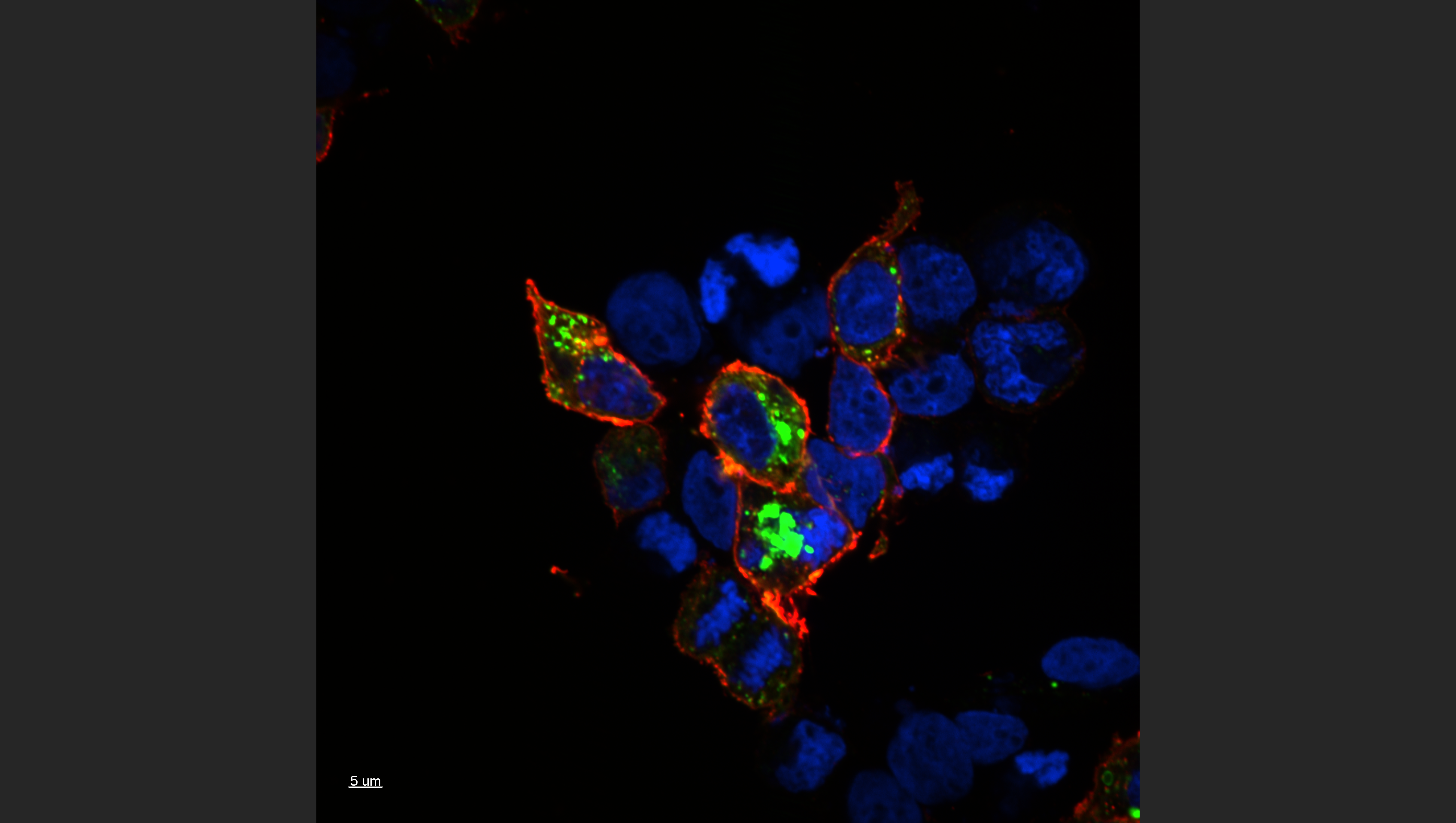

Supplement: Supplementary file 6 — Source data Fig. 4 [file 44321_2024_121_MOESM6_ESM.zip › Figure 4/Figure 4A/2020-07-11_11JUL20 HC LGR5 clone 1 5min_all.tif]

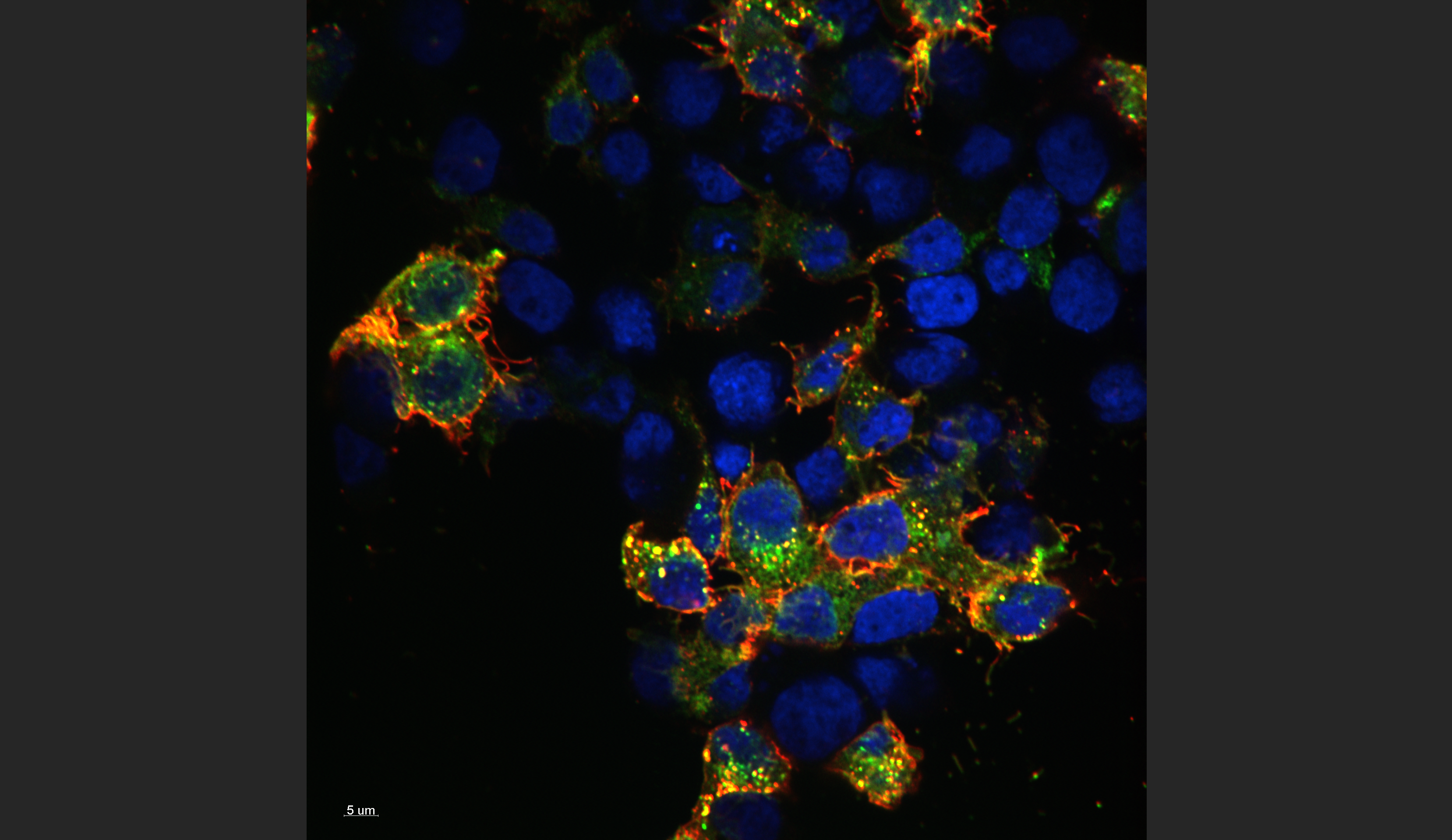

Supplement: Supplementary file 6 — Source data Fig. 4 [file 44321_2024_121_MOESM6_ESM.zip › Figure 4/Figure 4A/2020-07-11_11JUL20 HC LGR5 clone 1 45min_all.tif]

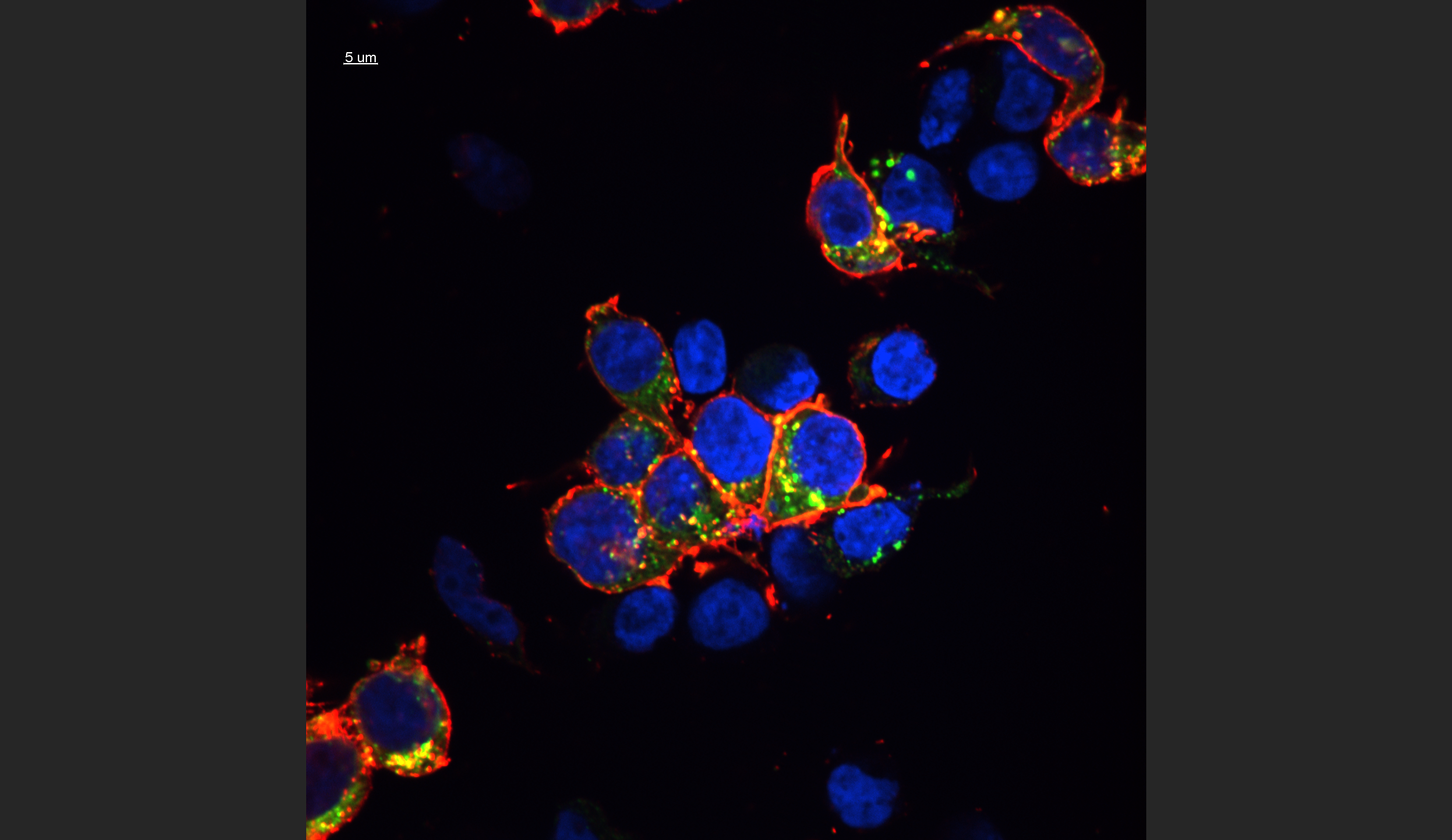

Supplement: Supplementary file 6 — Source data Fig. 4 [file 44321_2024_121_MOESM6_ESM.zip › Figure 4/Figure 4A/2020-07-11_11JUL20 HC LGR5 clone 1 15min_all.tif]

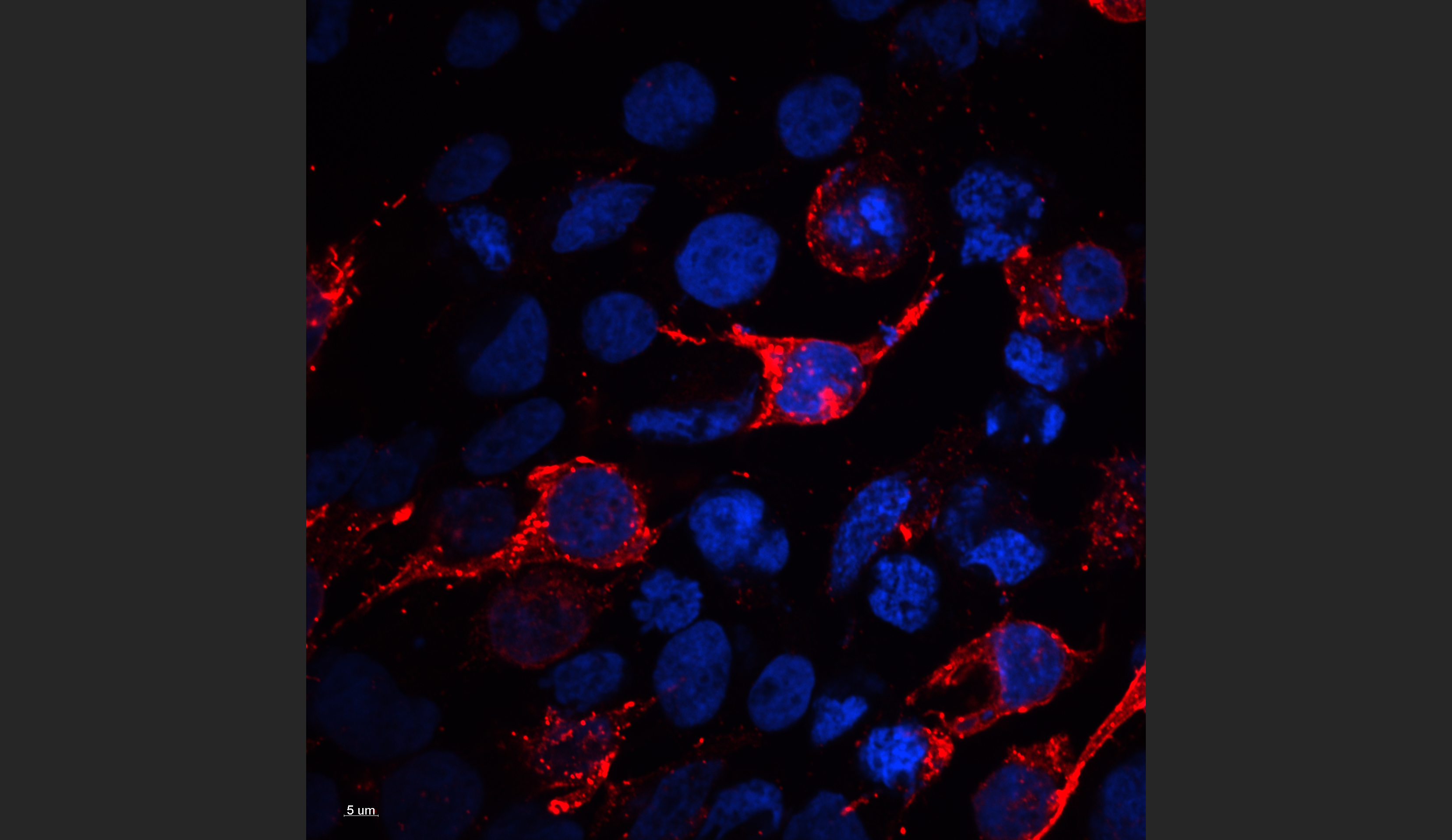

Supplement: Supplementary file 6 — Source data Fig. 4 [file 44321_2024_121_MOESM6_ESM.zip › Figure 4/Figure 4A/2020-07-11_11JUL20 HC LGR5 clone 1 30min_red.tif]

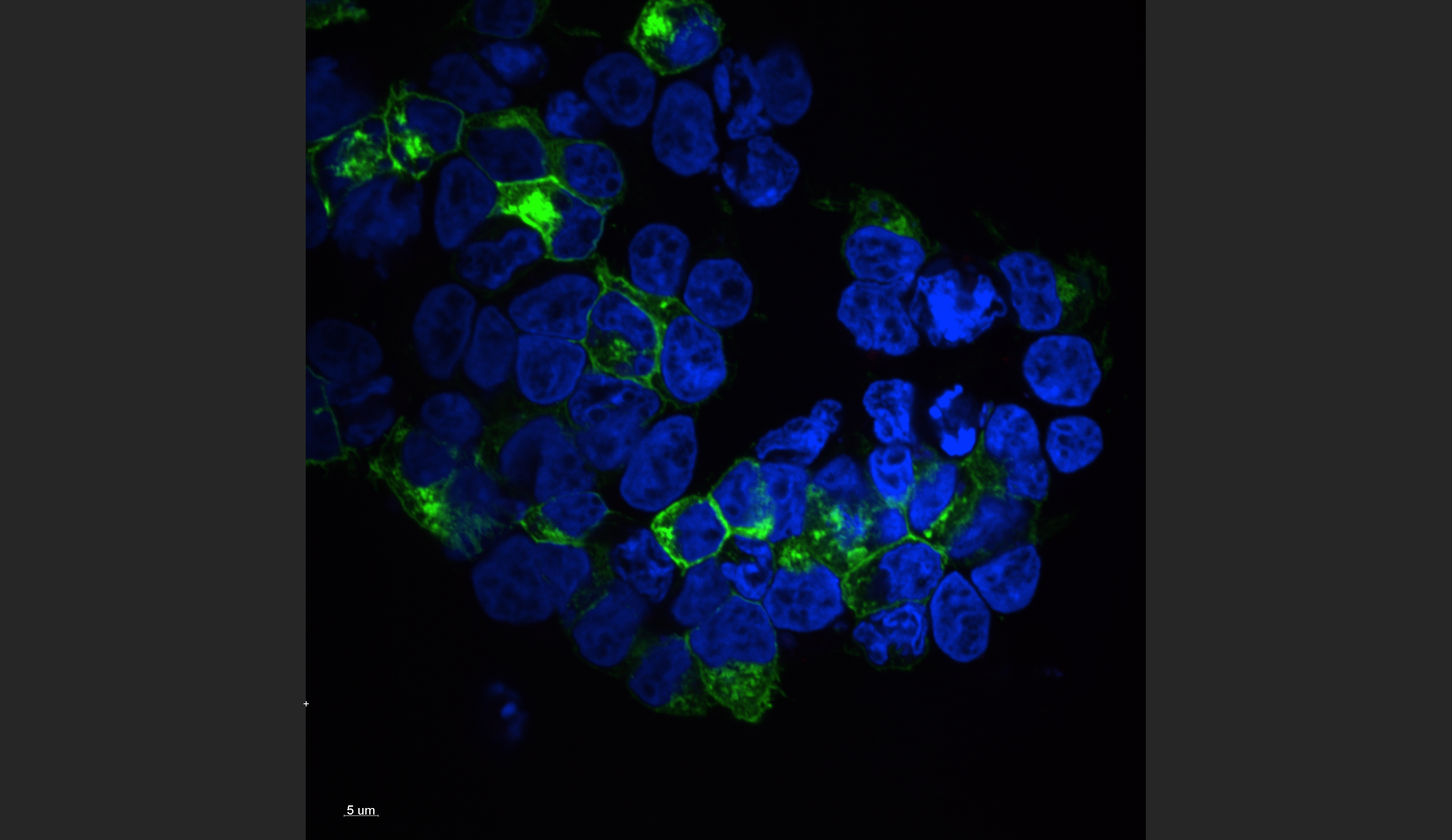

Supplement: Supplementary file 6 — Source data Fig. 4 [file 44321_2024_121_MOESM6_ESM.zip › Figure 4/Figure 4A/2020-07-11_11JUL20 HC LGR4 clone 1 45min_all.tif]

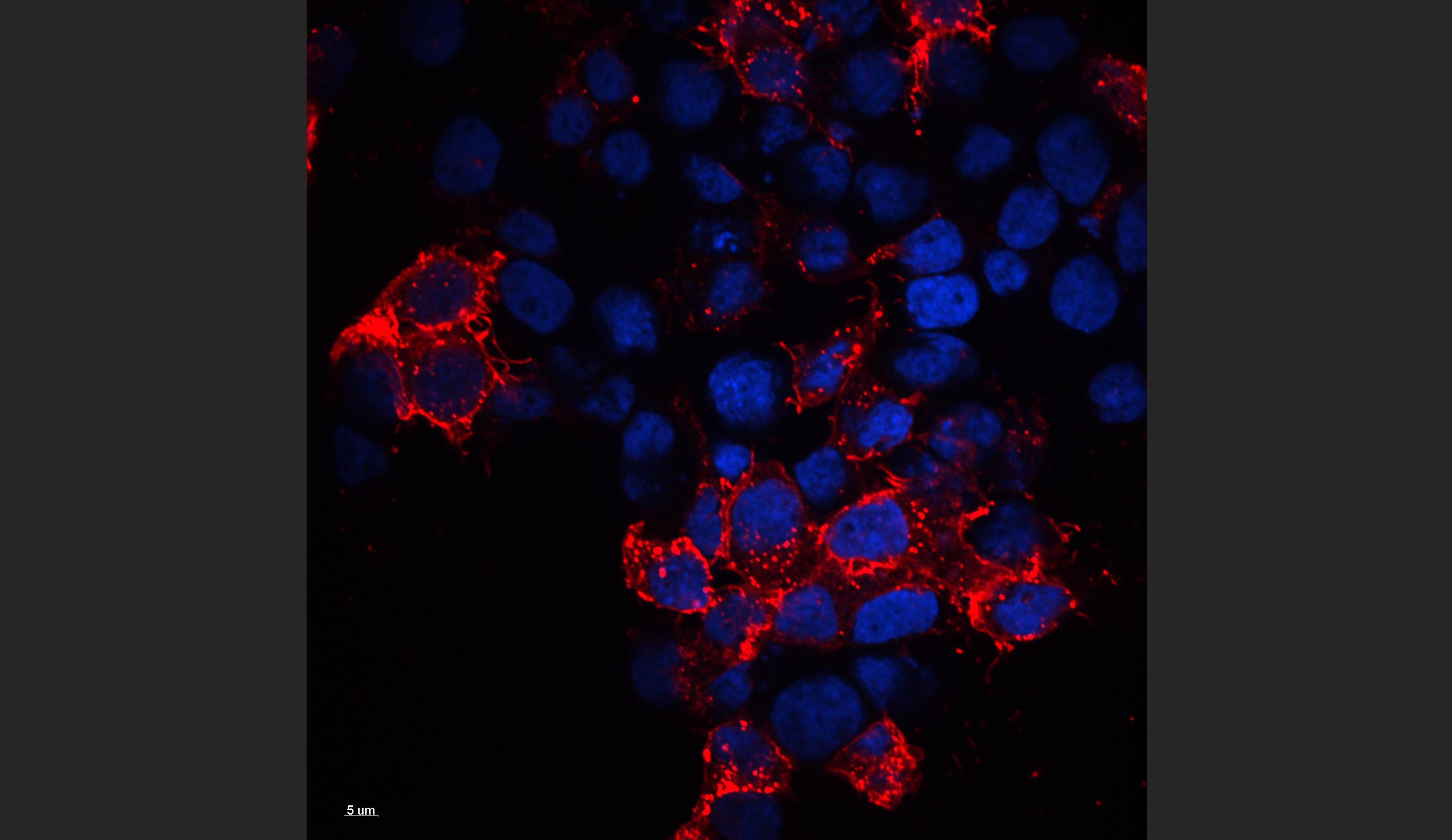

Supplement: Supplementary file 6 — Source data Fig. 4 [file 44321_2024_121_MOESM6_ESM.zip › Figure 4/Figure 4A/2020-07-11_11JUL20 HC LGR5 clone 1 45min_red.tif]

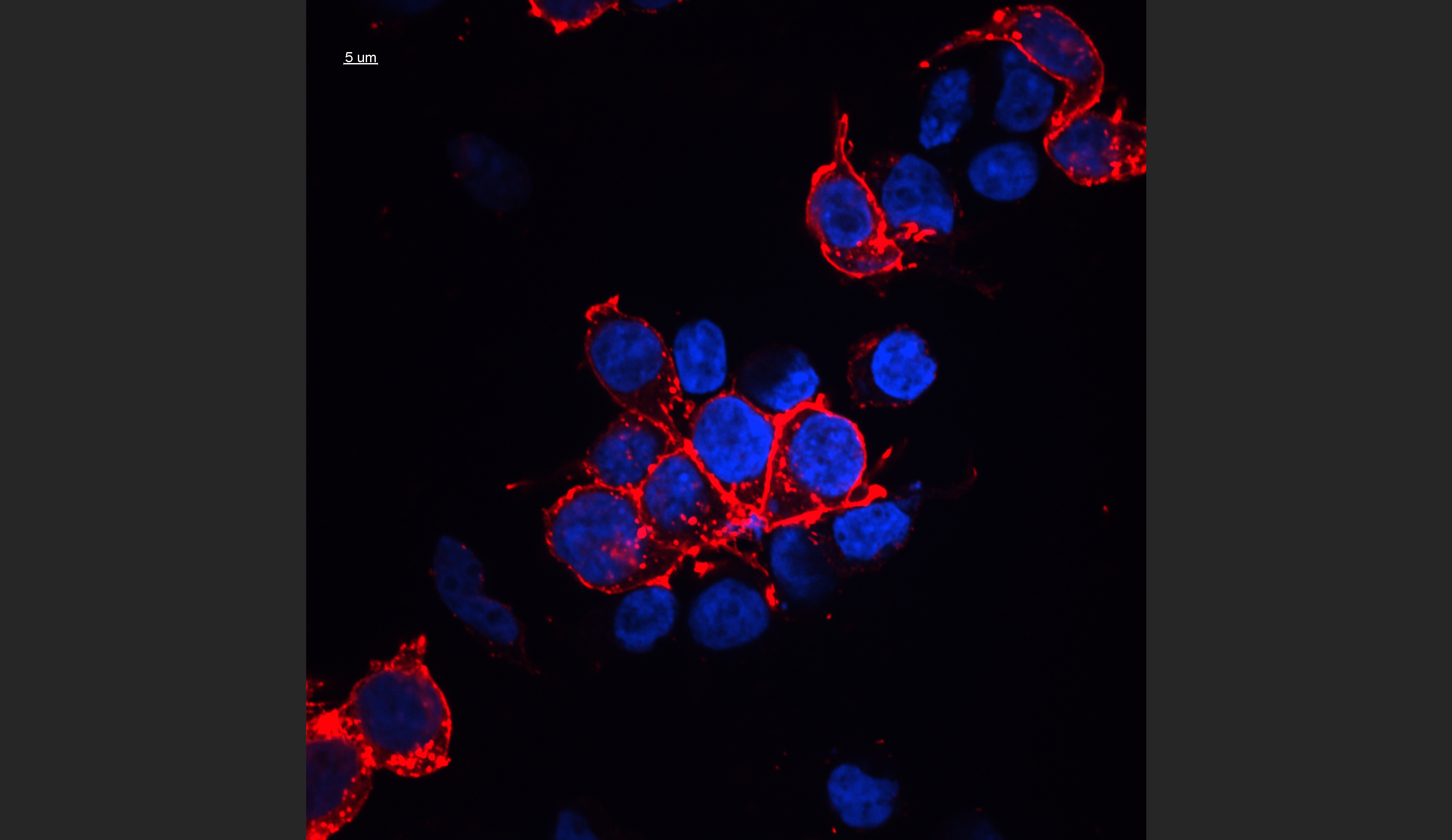

Supplement: Supplementary file 6 — Source data Fig. 4 [file 44321_2024_121_MOESM6_ESM.zip › Figure 4/Figure 4A/2020-07-11_11JUL20 HC LGR5 clone 1 15min_red.tif]

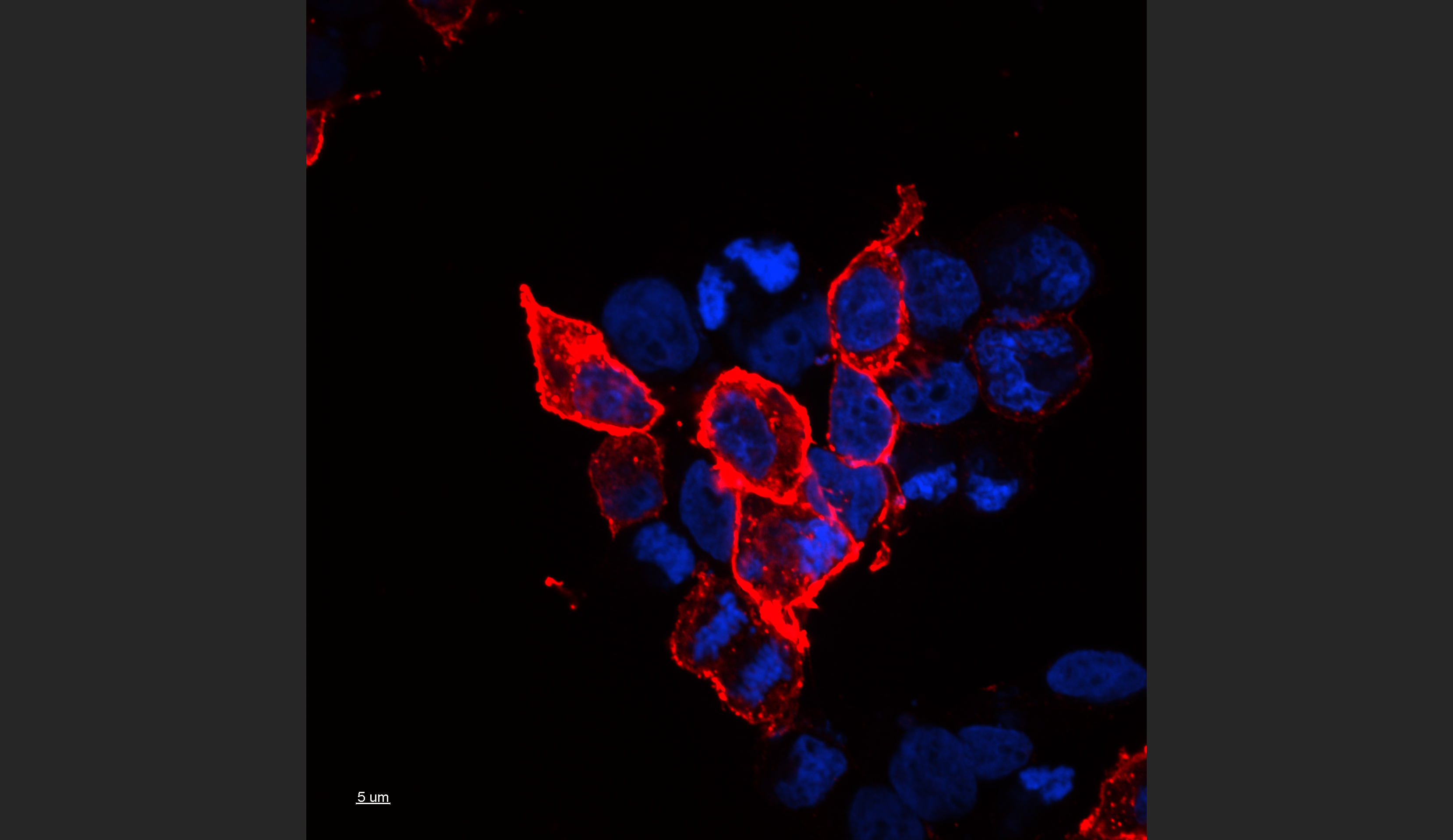

Supplement: Supplementary file 6 — Source data Fig. 4 [file 44321_2024_121_MOESM6_ESM.zip › Figure 4/Figure 4A/2020-07-11_11JUL20 HC LGR5 clone 1 5min_red.tif]

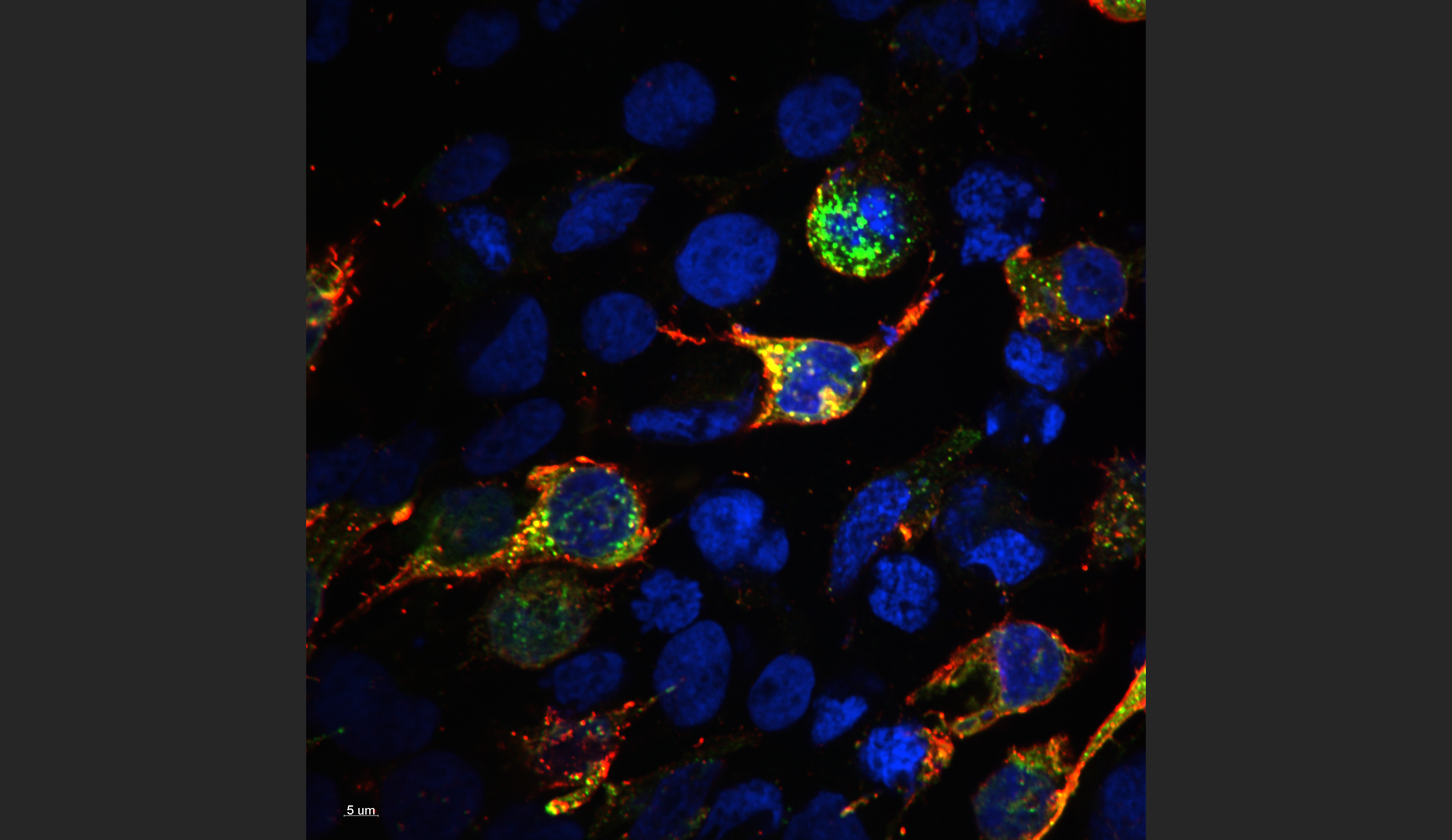

Supplement: Supplementary file 6 — Source data Fig. 4 [file 44321_2024_121_MOESM6_ESM.zip › Figure 4/Figure 4A/2020-07-11_11JUL20 HC LGR5 clone 1 30min_all.tif]

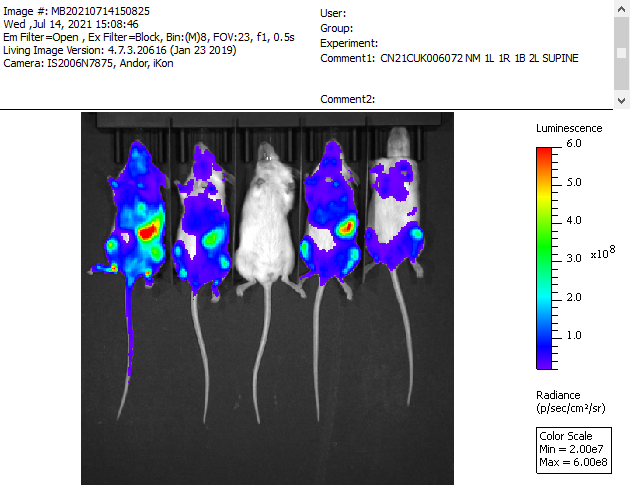

Supplement: Supplementary file 7 — Source data Fig. 5 [file 44321_2024_121_MOESM7_ESM.zip › Figure 5/Figure 5D_G/Cage6072.png]

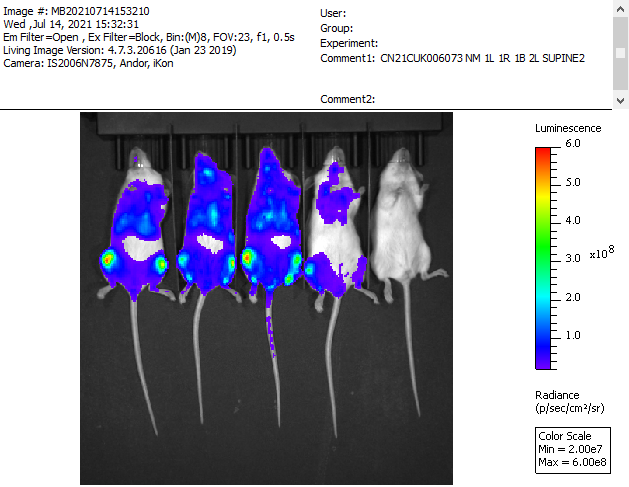

Supplement: Supplementary file 7 — Source data Fig. 5 [file 44321_2024_121_MOESM7_ESM.zip › Figure 5/Figure 5D_G/Cage6073.png]

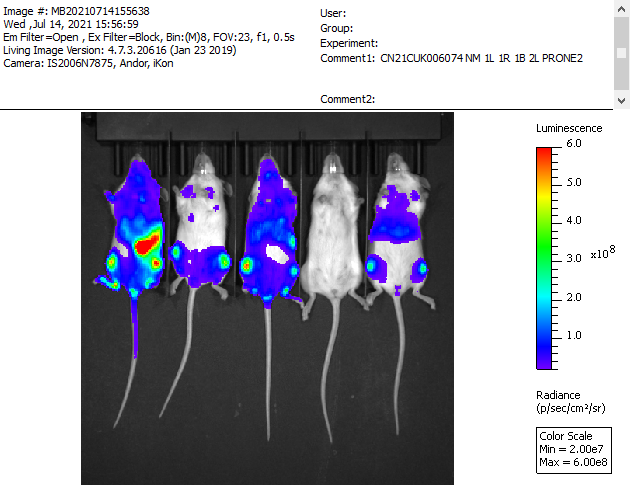

Supplement: Supplementary file 7 — Source data Fig. 5 [file 44321_2024_121_MOESM7_ESM.zip › Figure 5/Figure 5D_G/Cage6074.png]

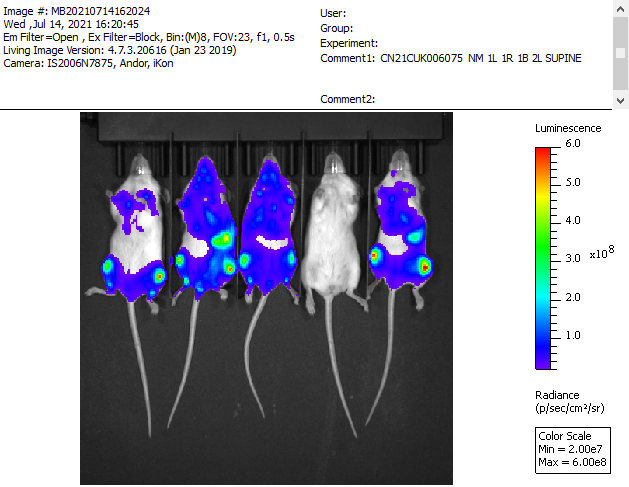

Supplement: Supplementary file 7 — Source data Fig. 5 [file 44321_2024_121_MOESM7_ESM.zip › Figure 5/Figure 5D_G/Cage6075.png]

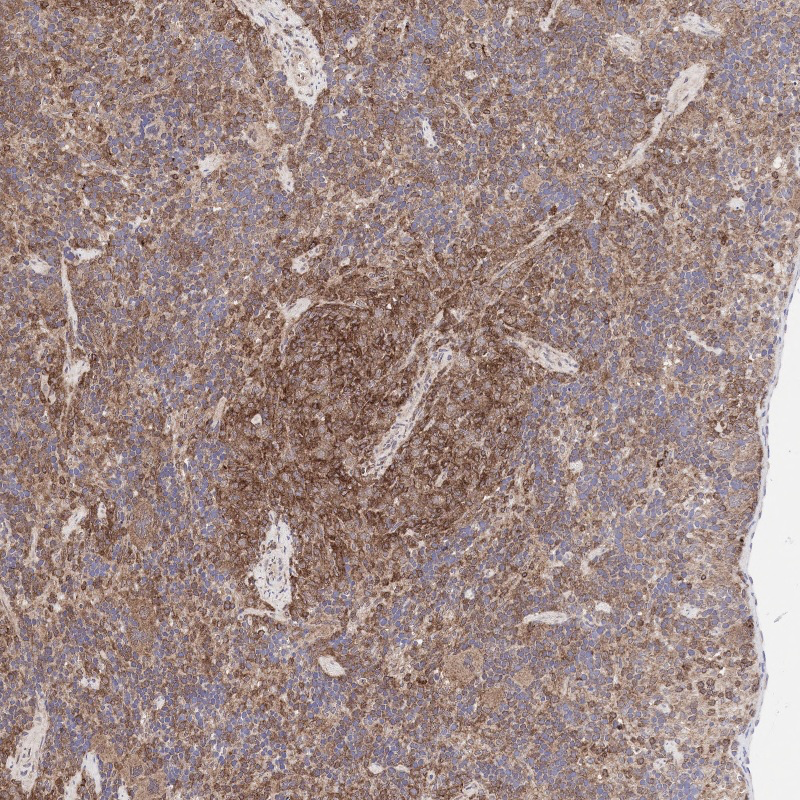

Supplement: Supplementary file 8 — Source data Fig. 6 [file 44321_2024_121_MOESM8_ESM.zip › Figure 6/Figure 6F/CL BiTE+PBMC LGR5 staining zoomed in.jpg]

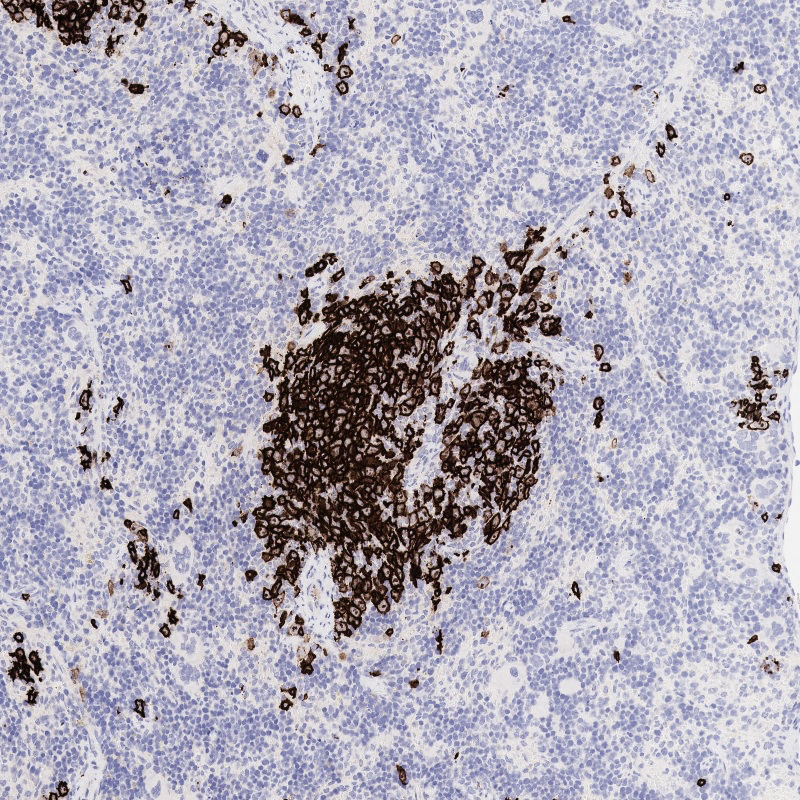

Supplement: Supplementary file 8 — Source data Fig. 6 [file 44321_2024_121_MOESM8_ESM.zip › Figure 6/Figure 6F/CL BiTE+PBMC CD20 staining zoomed in.jpg]

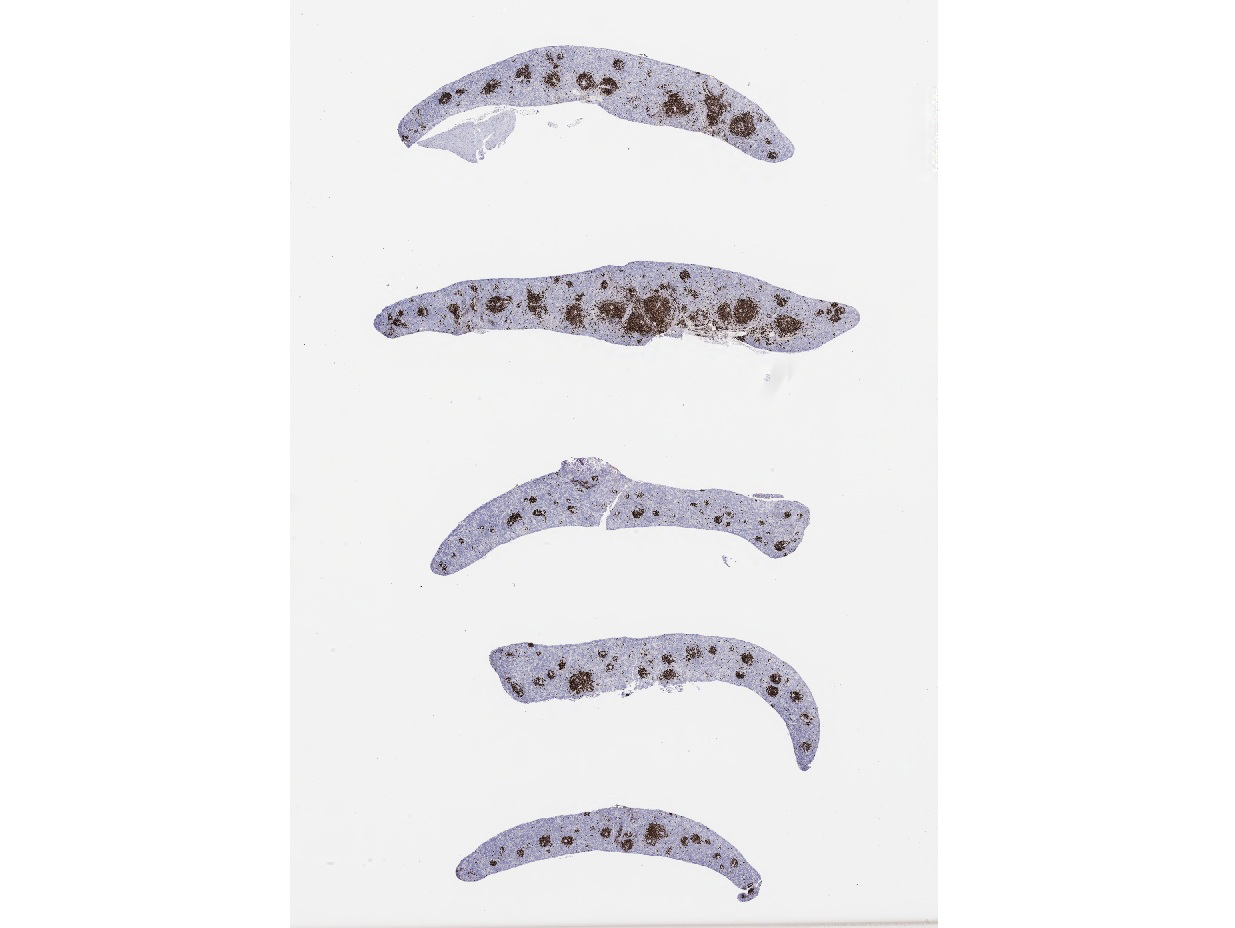

Supplement: Supplementary file 8 — Source data Fig. 6 [file 44321_2024_121_MOESM8_ESM.zip › Figure 6/Figure 6F/CL BiTE+PBMC CD20 staining.jpg]

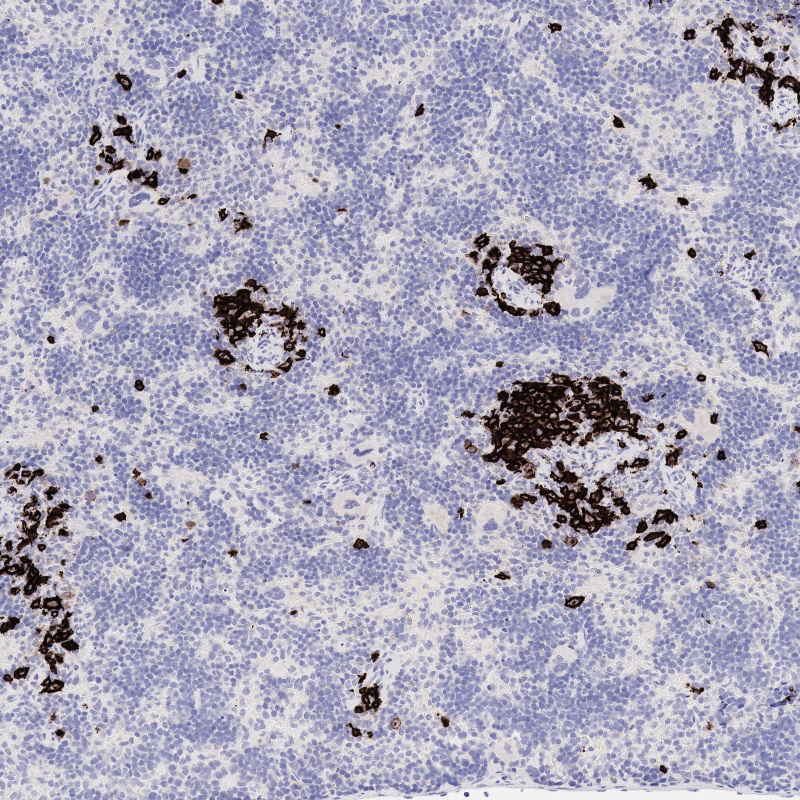

Supplement: Supplementary file 8 — Source data Fig. 6 [file 44321_2024_121_MOESM8_ESM.zip › Figure 6/Figure 6F/PBMC CD20 staining zoomed in.jpg]

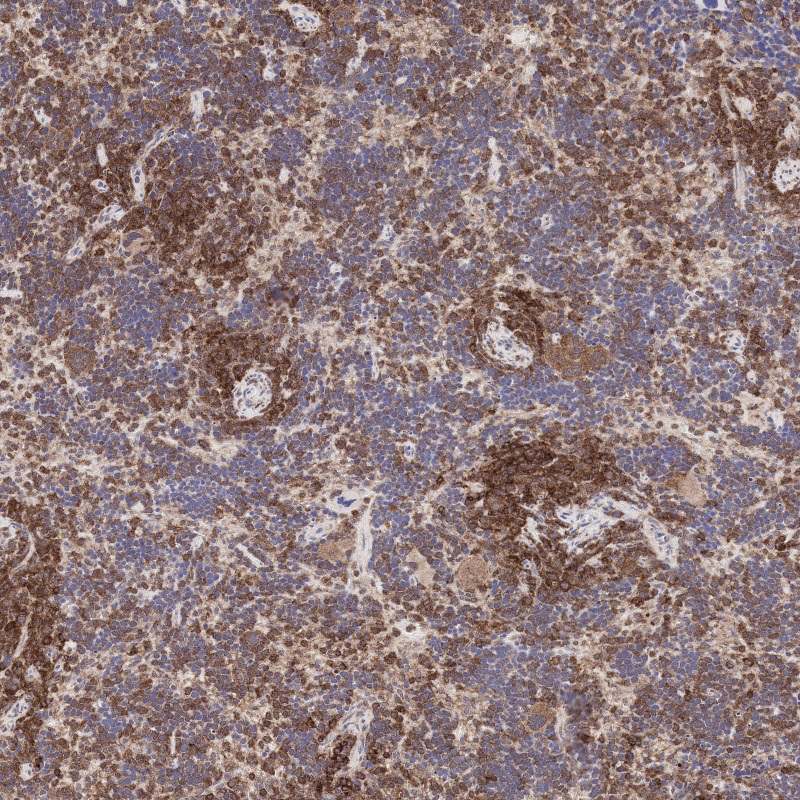

Supplement: Supplementary file 8 — Source data Fig. 6 [file 44321_2024_121_MOESM8_ESM.zip › Figure 6/Figure 6F/PBMC LGR5 staining zoomed in.jpg]

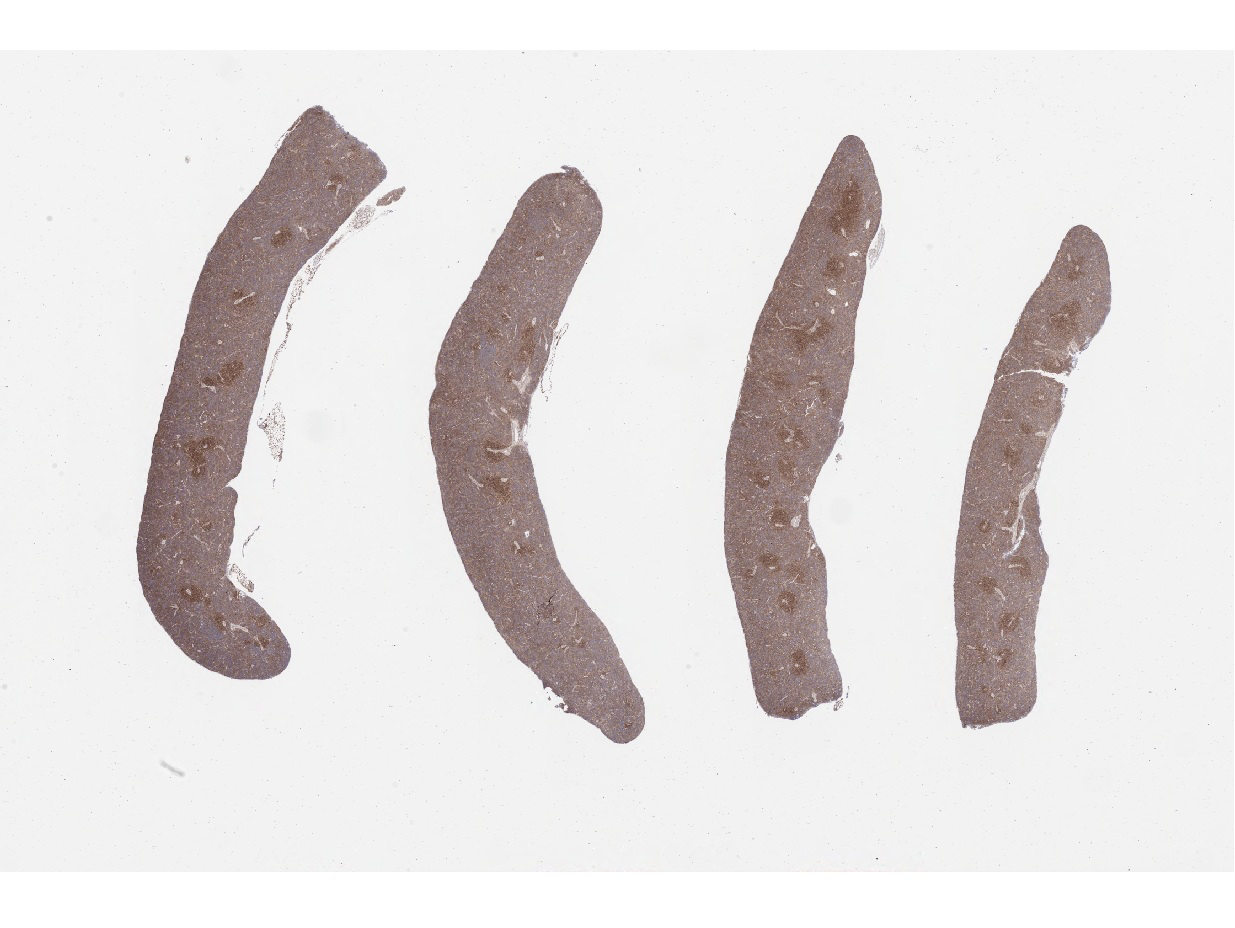

Supplement: Supplementary file 8 — Source data Fig. 6 [file 44321_2024_121_MOESM8_ESM.zip › Figure 6/Figure 6F/PBMC LGR5 staining.jpg]

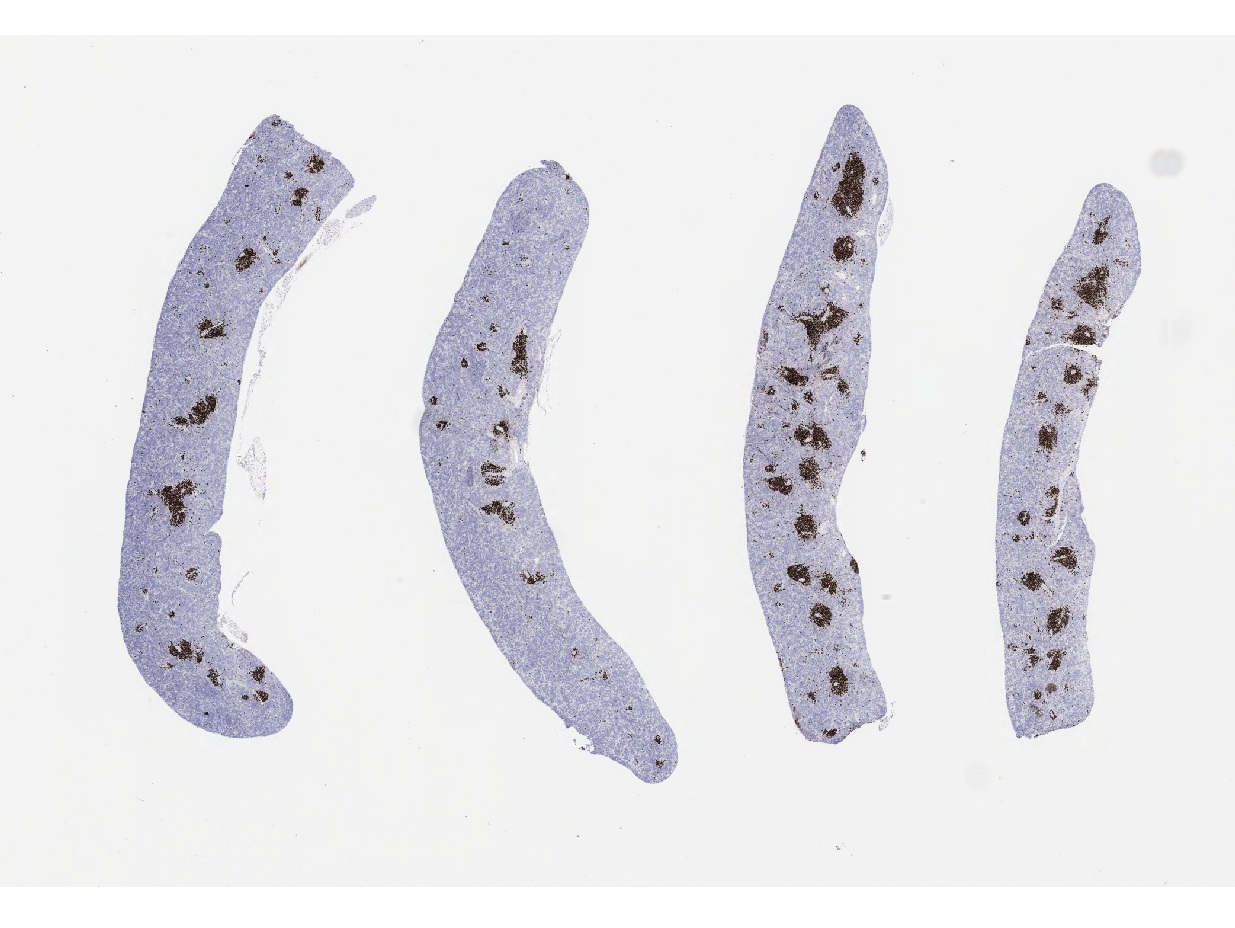

Supplement: Supplementary file 8 — Source data Fig. 6 [file 44321_2024_121_MOESM8_ESM.zip › Figure 6/Figure 6F/PBMC CD20 staining.jpg]

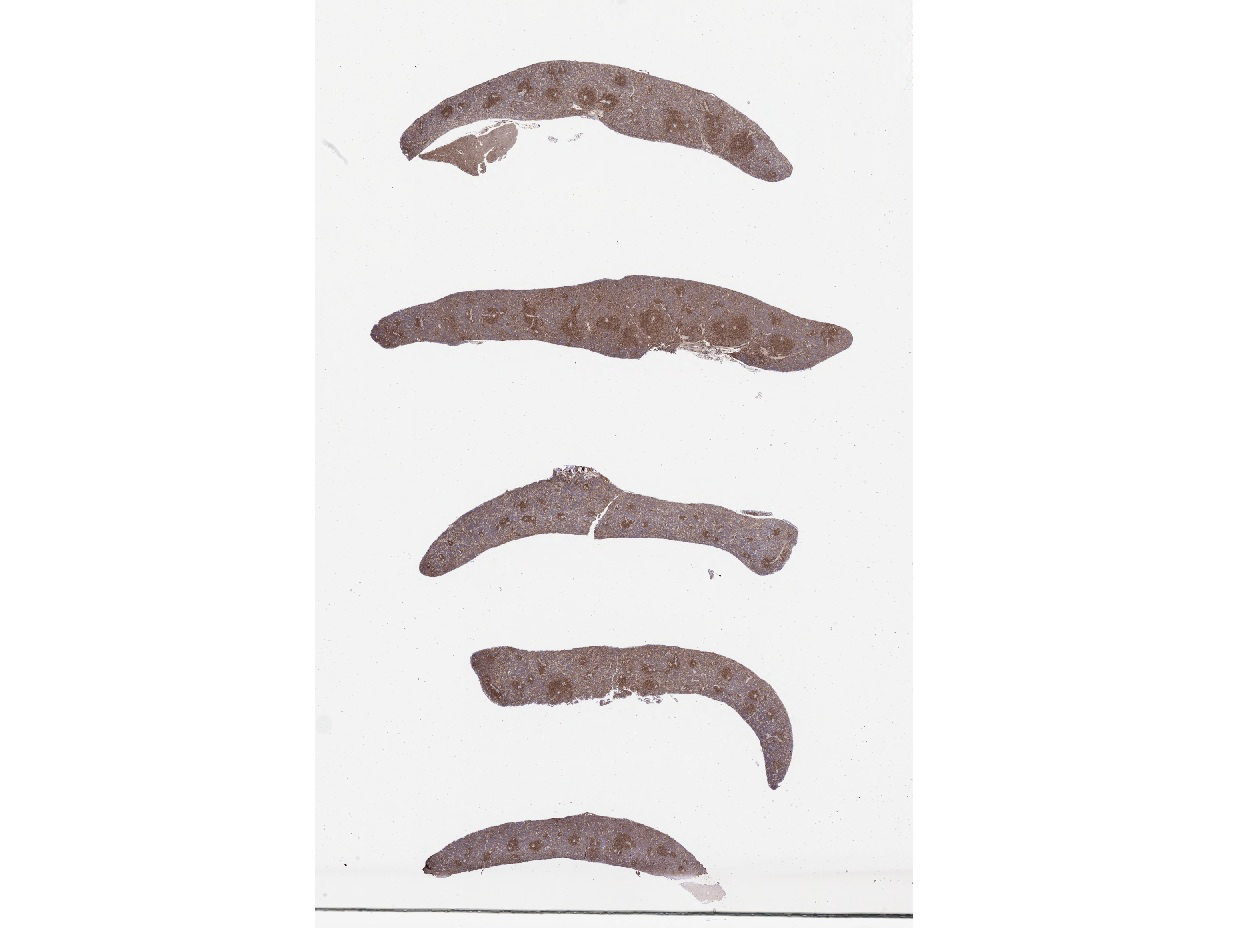

Supplement: Supplementary file 8 — Source data Fig. 6 [file 44321_2024_121_MOESM8_ESM.zip › Figure 6/Figure 6F/CL BiTE+PBMC LGR5 staining.jpg]

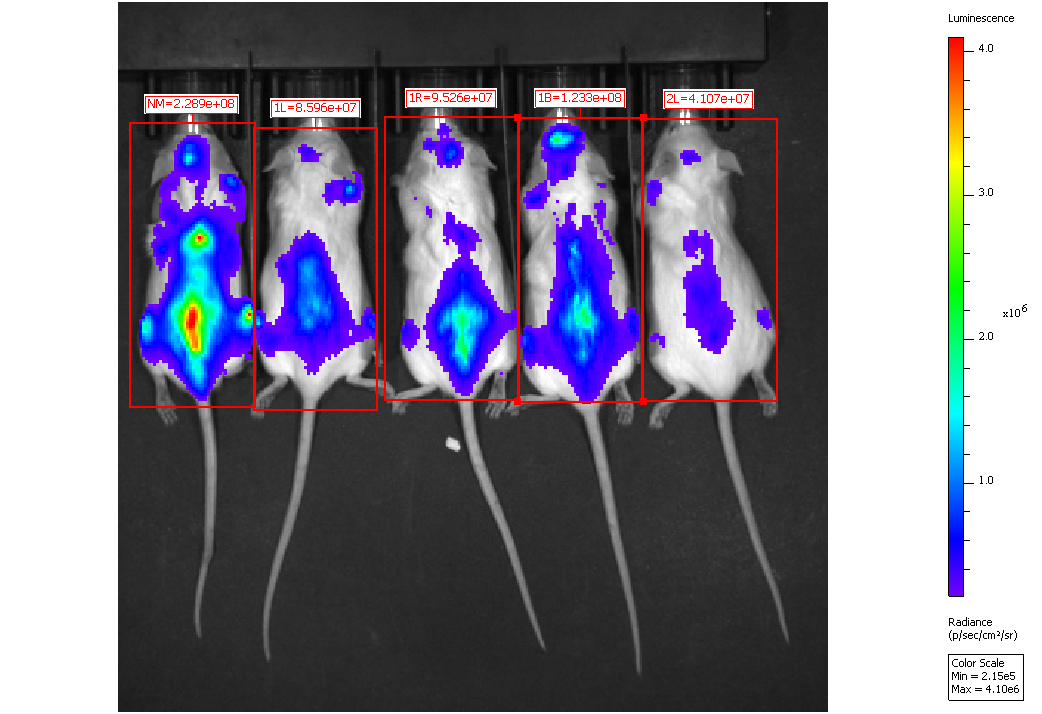

Supplement: Supplementary file 8 — Source data Fig. 6 [file 44321_2024_121_MOESM8_ESM.zip › Figure 6/Figure 6_D_E/AV20230609095159.PNG]

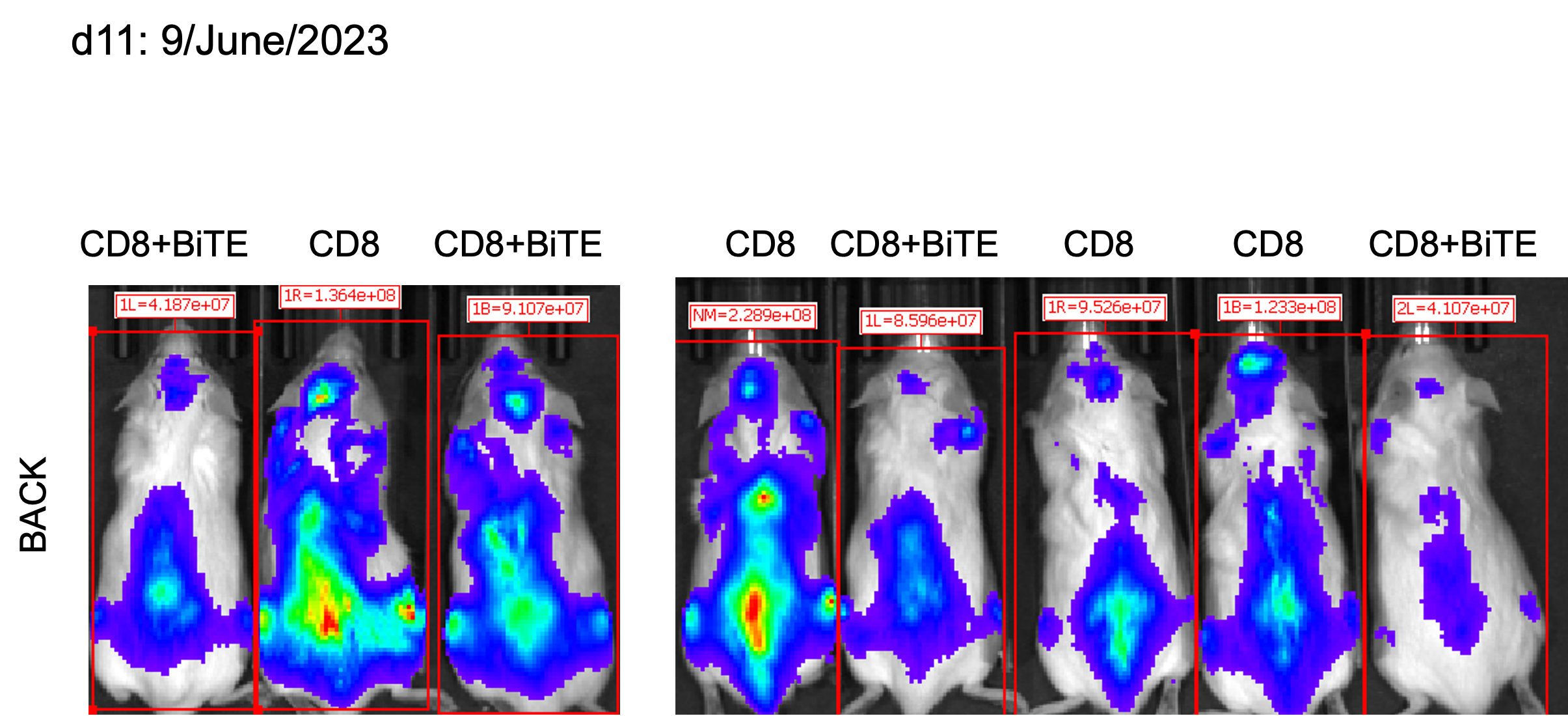

Supplement: Supplementary file 8 — Source data Fig. 6 [file 44321_2024_121_MOESM8_ESM.zip › Figure 6/Figure 6_D_E/Figure6_D_IVIS.png]

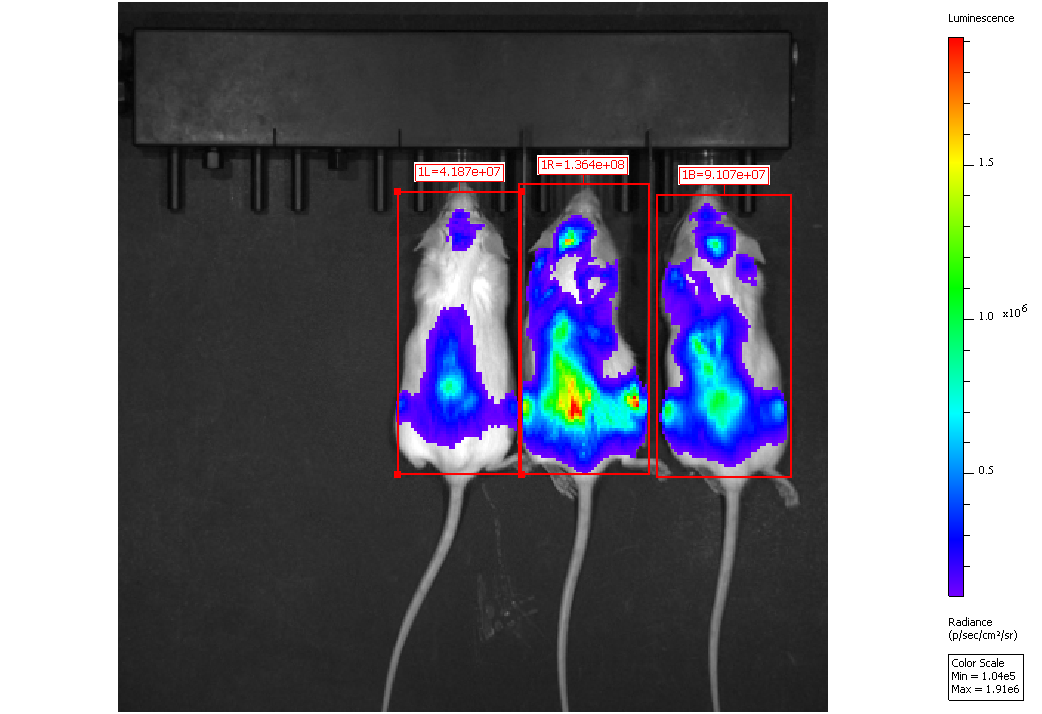

Supplement: Supplementary file 8 — Source data Fig. 6 [file 44321_2024_121_MOESM8_ESM.zip › Figure 6/Figure 6_D_E/AV20230609104148.PNG]

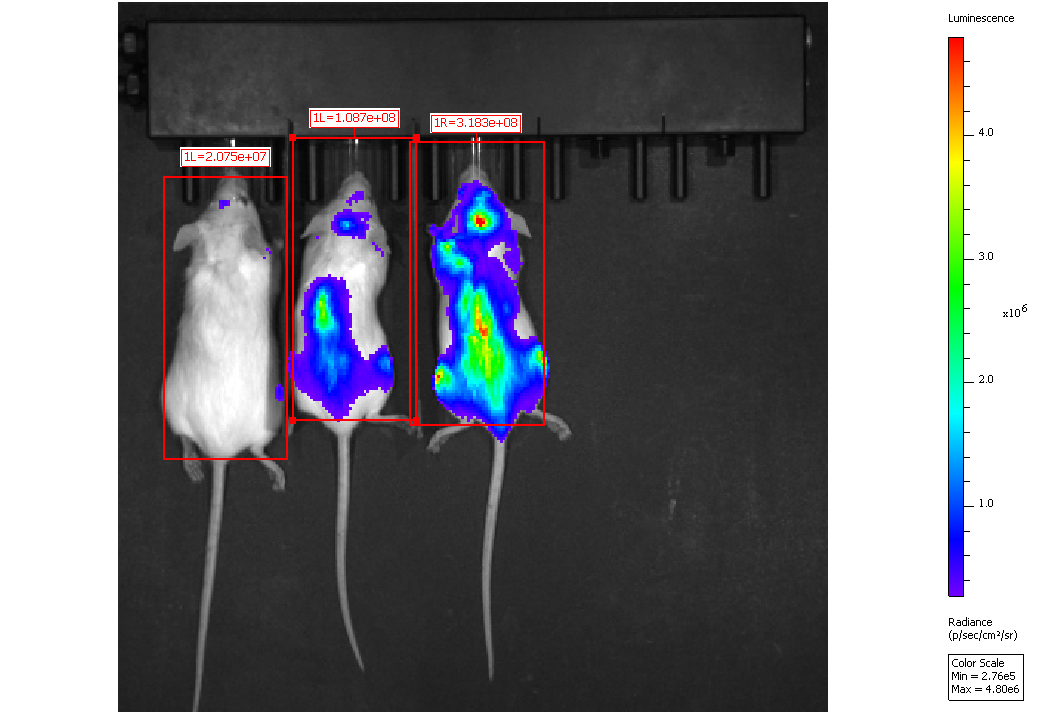

Supplement: Supplementary file 9 — Source data Fig. 7 [file 44321_2024_121_MOESM9_ESM.zip › Figure 7/Figure 7D/AV20230608113710.PNG]

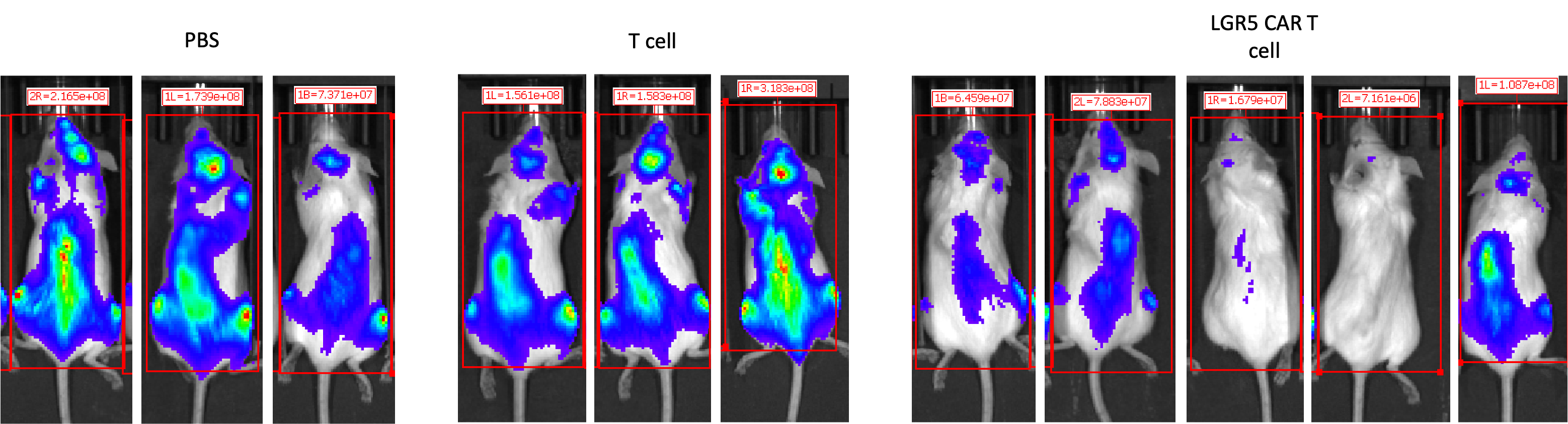

Supplement: Supplementary file 9 — Source data Fig. 7 [file 44321_2024_121_MOESM9_ESM.zip › Figure 7/Figure 7D/Figure 7D.png]

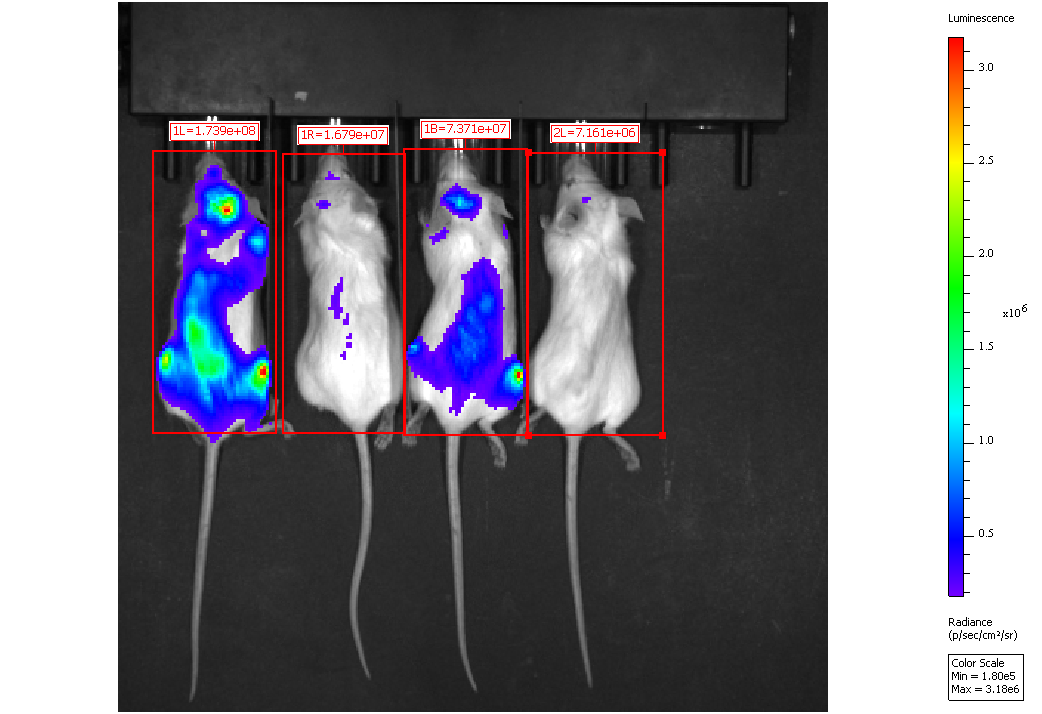

Supplement: Supplementary file 9 — Source data Fig. 7 [file 44321_2024_121_MOESM9_ESM.zip › Figure 7/Figure 7D/AV20230608111653.PNG]

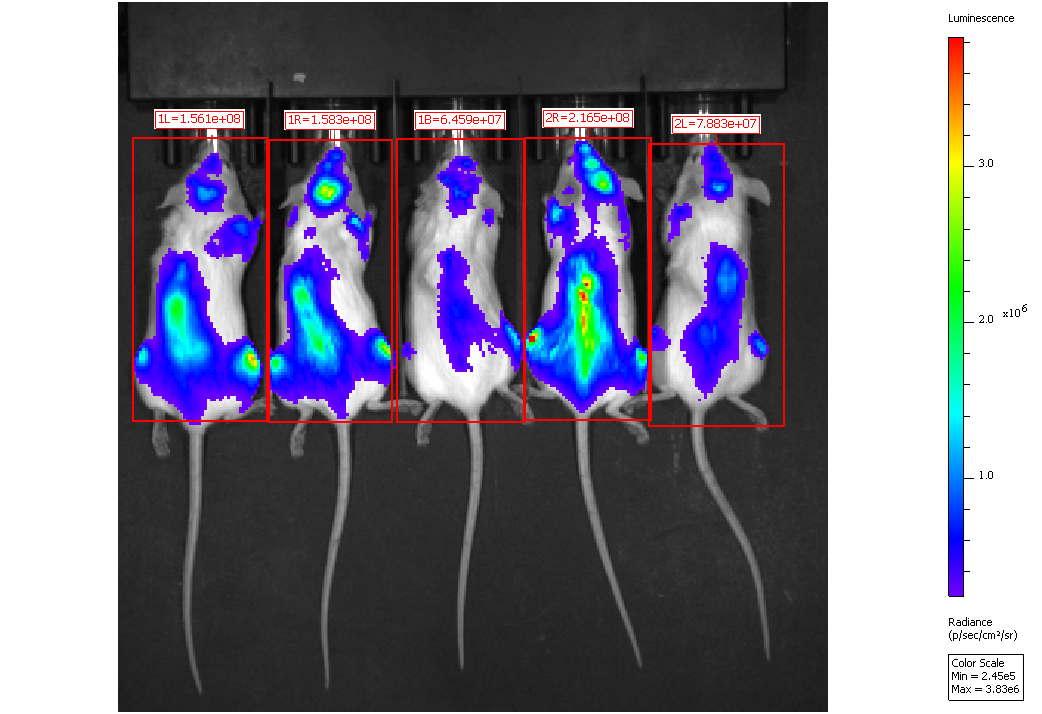

Supplement: Supplementary file 9 — Source data Fig. 7 [file 44321_2024_121_MOESM9_ESM.zip › Figure 7/Figure 7D/AV20230608110414.PNG]
