# Supplementary material for: Novel immunotherapeutics against LGR5 to target multiple cancer types
Source: EMBO Mol Med. 2024 Aug 21;16(9):2233–61. doi: 10.1038/s44321-024-00121-2 (PMC11393416; doi:10.1038/s44321-024-00121-2)
Supplement: Supplementary file 5 — Source data Fig. 3 [file 44321_2024_121_MOESM5_ESM.zip › Figure 3/Figure 3B/Figure3B_compiled_Western_Blots_ROI_highlighted_in_red.pdf]

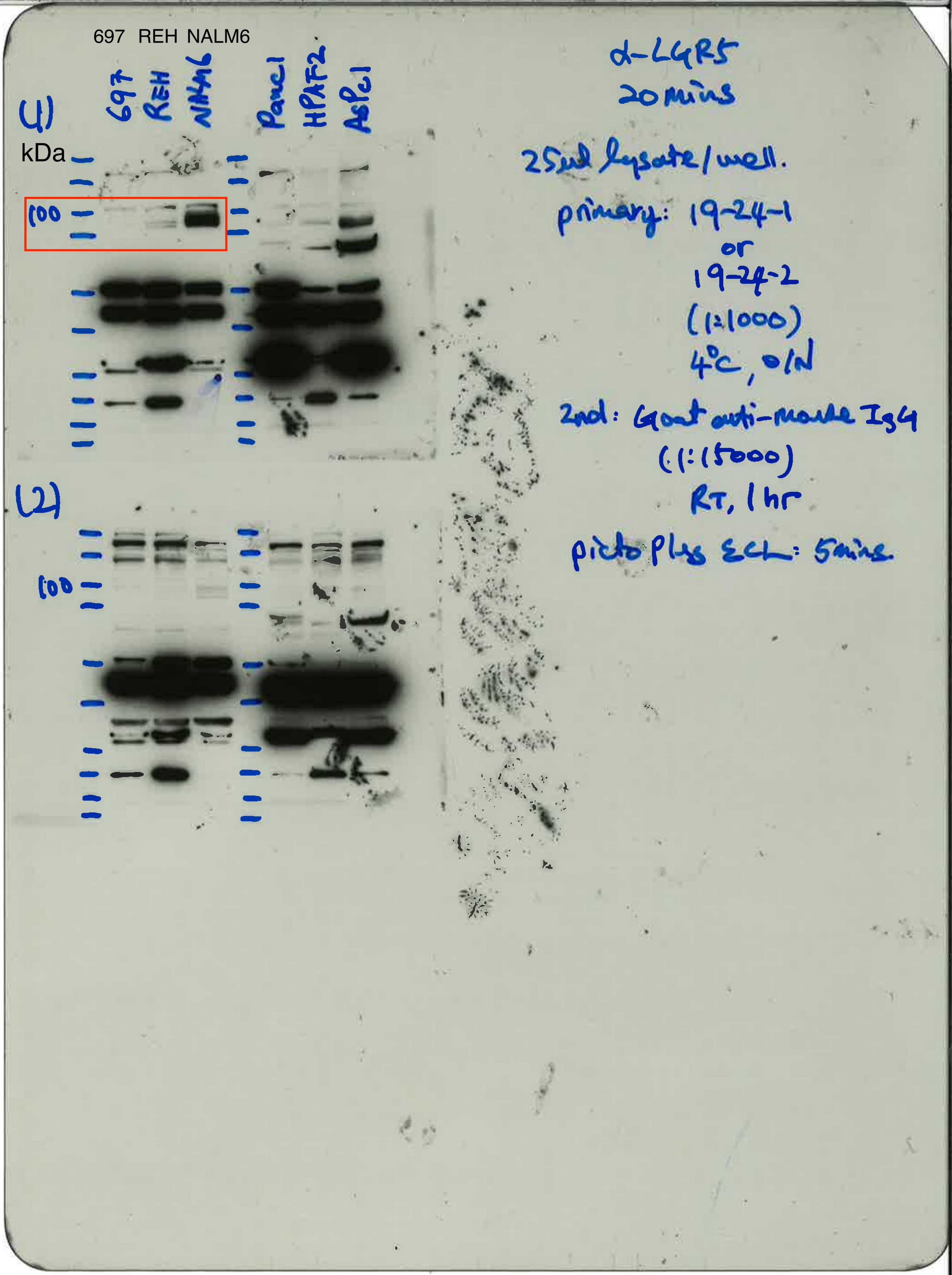
697 REH NALM6
(1)
kDa
697
REH
NALM6
Panc1
HPAF2
AsPc1
100
(2)
100
α-LGR5
20mins
25ul lysate/well.
primary: 19-24-1
or
19-24-2
(1:1000)
4°C, o/N
2nd: Goat anti-mouse IgG
(1:15000)
RT, 1hr
picoPlus ECL: 5mins.

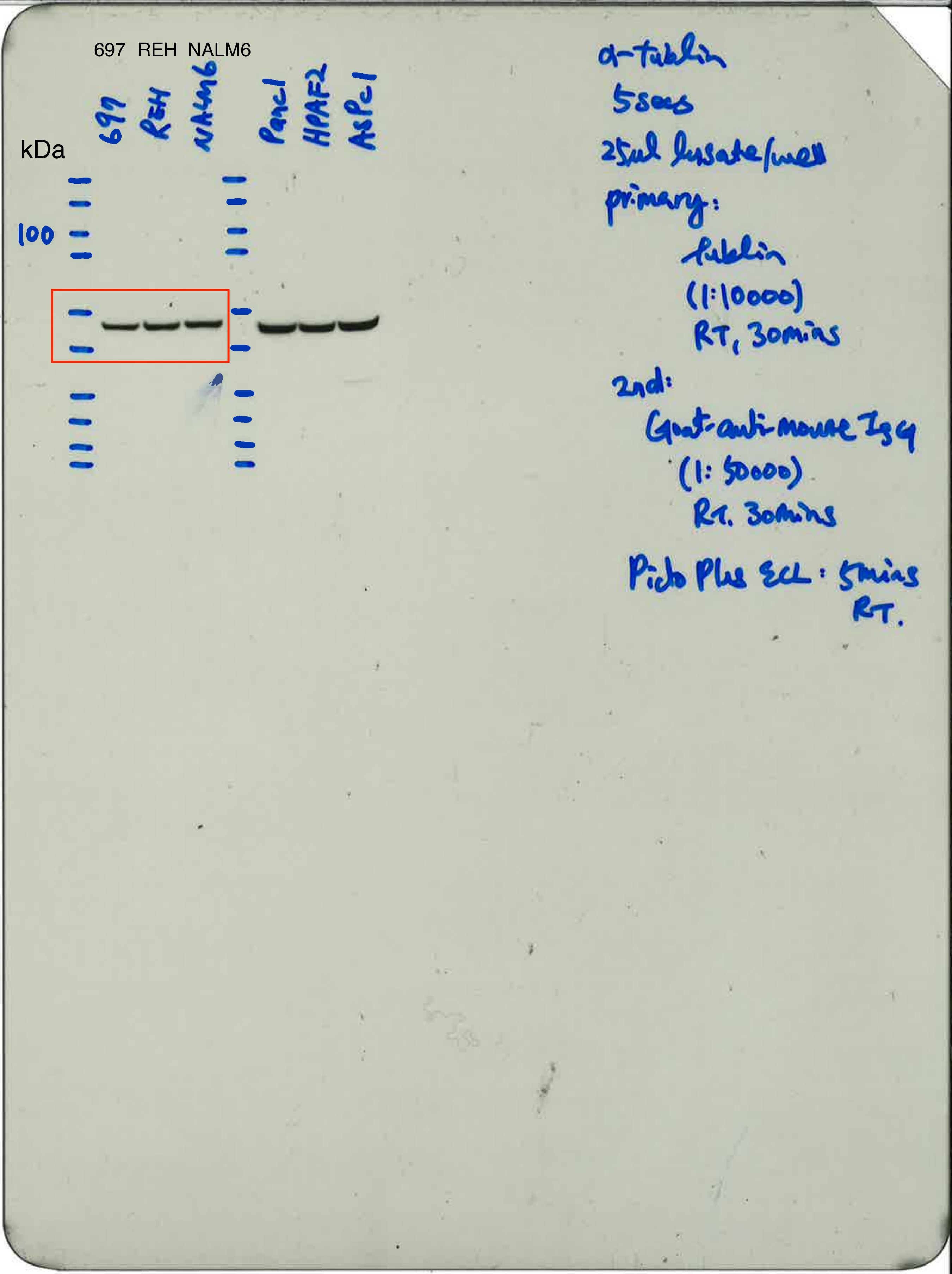
697 REH NALM6
kDa
100
697
REH
NALM6
Panc1
HPAF2
AsPc1
α-Tubulin
5secs
25ul lysate/well
primary:
tubulin
(1:10000)
RT, 30mins
2nd:
Goat-anti-mouse IgG
(1: 50000)
RT. 30mins
Pico Plus ECL: 5mins
RT.
