# Supplementary material for: Novel immunotherapeutics against LGR5 to target multiple cancer types
Source: EMBO Mol Med. 2024 Aug 21;16(9):2233–61. doi: 10.1038/s44321-024-00121-2 (PMC11393416; doi:10.1038/s44321-024-00121-2)
Supplement: Supplementary file 11 — Expanded View Figures [file 44321_2024_121_MOESM11_ESM.pdf]

Expanded View Figures

Figure EV1. Specificity of LGR5 antibodies generated in the study.

(**A**) Amino acid sequence of the human LGR5 antigen used for mouse immunisation and generation of α-LGR5; numbering starts at Gly1 in the processed human LGR5 (hLGR5), lacking the signal sequence. The sequence is annotated with the Fragments used in the RAD display experiments that map the α-LGR5 epitope to Frag1A. Fragments do not cover sequences that match the murine Lgr5. *Below* – location of the antigenic region (in red) within the structure of the extracellular domain of LGR5 (atomic coordinates for the model taken from (Peng et al, 2013). (**B**) Configuration of the LGR family transgenic constructs used in the study. All expressed LGR proteins contain a common N-terminal hemagglutinin (HA) tag and fusion at the C-terminus to the vasopressin V2 receptor C-terminal tail (V2R) followed by eGFP. (**C**) Western blot analysis of HEK293T lysates expressing the murine LGR5 (mLGR5), the human LGR5 (hLGR5) and the *cynomolgus* LGR5 (cLGR5) probed with α-LGR5 hybridoma clones 1, 3 and 4 and antibodies to HA and vinculin, as noted. No specific immune reactivity was observed when probing the western blots with the other 14 hybridoma clones. (**D**) Sequence conservation amongst the α-LGR5 hybridoma clones within the complementary determining regions (CDRs). Conserved amino acids relative to α-LGR5 clone 1 for clones 2–4 are represented by a dash. Amino acid differences are indicated "X". (**E**) Western blot analysis of the Fragments delineated above (Fig. EV1A) as RAD-displayed fusion peptides using α-LGR5 hybridoma clones 1 (top panel), 3 (middle panel) and 4 (bottom panel). (**F**) Sequence alignment of the N-terminal 15 amino acids of human LGR5, corresponding to Frag1A, with the corresponding region in the other LGR family members. Sequences were aligned based on three invariant cysteine residues denoted by asterisks. The amino acid difference in the *cynomolgus* sequence is underlined. (**G**) Wnt pathway reporter assays (TopFlash assays) for HEK293T cells transfected with either eGFP or human LGR5-eGFP (hLGR5-eGFP), treated with Wnt3A ligand, R-spondin and either IgG1 or α-LGR5 at levels of approximately 10-fold molar excess over Wnt3A ligand. ns, no significant difference. Data is presented as mean expression, $+/-$ SD for 3 biological replicates. *ns*, no significant difference in Wnt pathway reporter activity. (**H**) Immunofluorescent detection of HEK293T cells expressing transgenic LGR4-eGFP or LGR5-eGFP (*left panels*, green) using Fl-α-LGR5 (*middle panels*, red). *Right panels* merged fluorescent signals. Scale bars, 10 µm. (**I**) Flow cytometric analysis of HEK293T cells expressing mLGR4-eGFP (*top left*), mLGR5-eGFP (*top right*) and hLGR5-eGFP (*bottom panels*) using Fl-α-LGR5. For analysis of the hLGR5-eGFP expressing HEK293T cells, Fl-α-LGR5 was pre-incubated with either RAD-Frag1A or RAD-Frag1B (*bottom left and right*). Source data are available online for this figure.

© The Author(s)

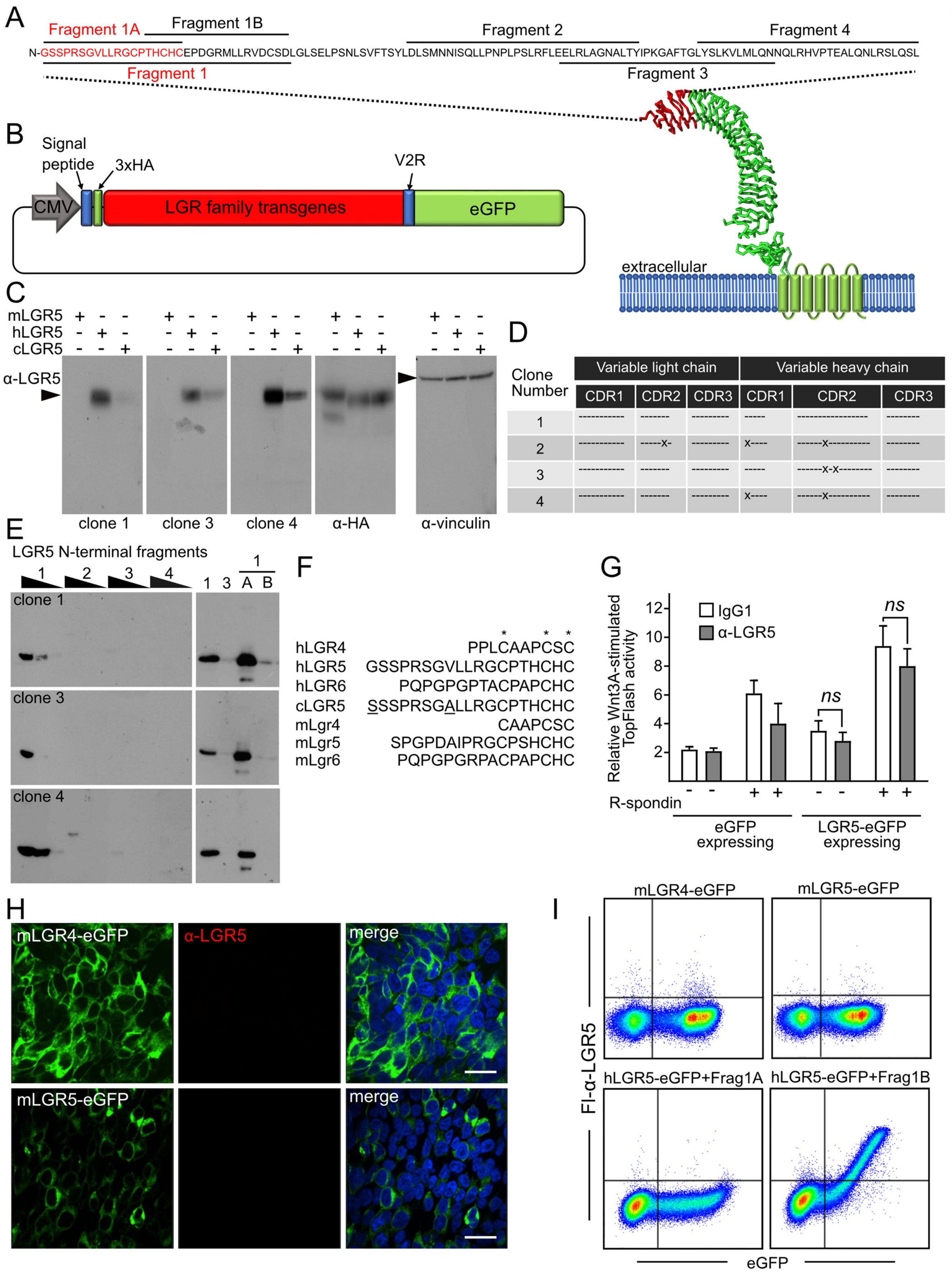
A
Fragment 1A
Fragment 1B
Fragment 2
Fragment 4
N-GSSPRSGVLLRGCPTHCHCEPDGRMLLRVDCSDLGLSELPSNLSVFTSYLDLSMNNISQLLPNPLPSLRFLEELRLAGNALTYIPKGAFTGLYSLKVLMLQNNQLRHVPTEALQNLRSLQSL
Fragment 1
Fragment 3
B
Signal peptide
3xHA
V2R
CMV
LGR family transgenes
eGFP
extracellular
C
mLGR5
hLGR5
cLGR5
α-LGR5
clone 1
clone 3
clone 4
α-HA
α-vinculin
D
Clone Number
Variable light chain
Variable heavy chain
CDR1
CDR2
CDR3
CDR1
CDR2
CDR3
1
2
3
4
E
LGR5 N-terminal fragments
1 2 3 4
1 3 A B
clone 1
clone 3
clone 4
F
hLGR4 PPLCAAPCSC
hLGR5 GSSPRSGVLLRGCPTHCHC
hLGR6 PQPGPGPTACPAPCHC
cLGR5 SSSPRSGALLRGCPTHCHC
mLgr4 CAAPCSC
mLgr5 SPGPDAIPRGCPSHCHC
mLgr6 PQPGPGRPACPAPCHC
G
Relative Wnt3A-stimulated TopFlash activity
IgG1
α-LGR5
ns
ns
R-spondin
eGFP expressing
LGR5-eGFP expressing
H
mLGR4-eGFP
α-LGR5
merge
mLGR5-eGFP
merge
I
mLGR4-eGFP
mLGR5-eGFP
hLGR5-eGFP+Frag1A
hLGR5-eGFP+Frag1B
FI-α-LGR5
eGFP

 © The Author(s)

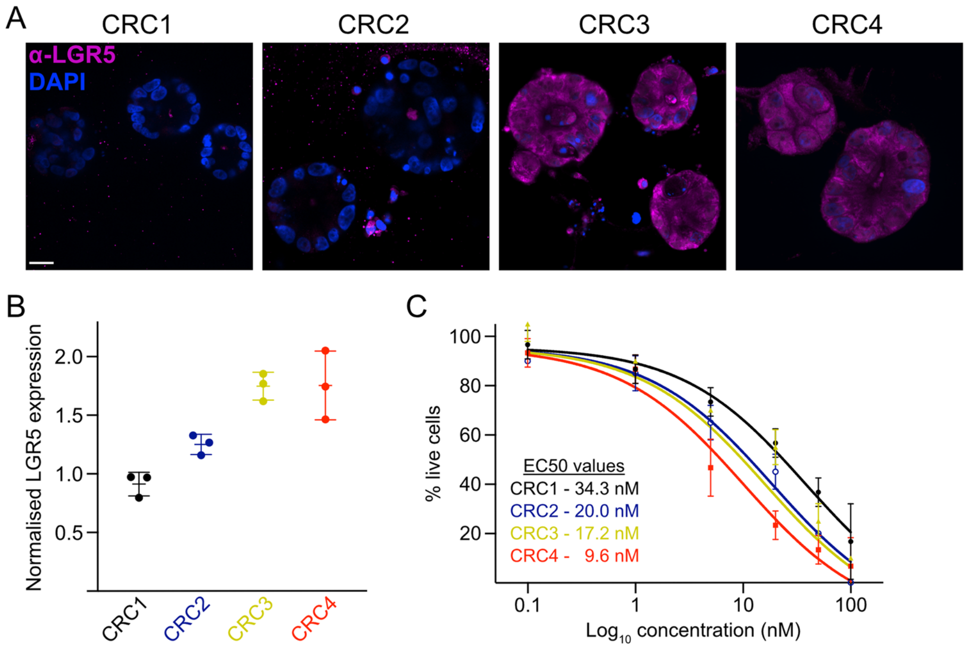

Figure EV2. Sensitivity of CRC organoid models expressing variable LGR5 levels to α-LGR5v4-ADC treatment.

(**A**) Immunofluorescent imaging of LGR5 in CRC organoid models. Images are representative of 2 independent experiments. Scale bar, 20 µm. (**B**) Relative *LGR5* transcript levels in the CRC organoids models measured by quantitative qRT-PCR with *TBP* as a reference gene. Data is presented as mean expression, +/− SD for 3 biological replicates. (**C**) CRC organoid model killing with α-LGR5v4-ADC treatment quantified as the percent of CRC organoids at each treatment level that displayed more than 20% of cleaved caspase 3 positive component cells. Data for treatment of each organoid model is derived from a minimum of 10 datapoints at each concentration of α-LGR5v4-ADC with 2–3 independent biological replicates. Data is presented as mean expression, +/− SD. Source data are available online for this figure.

© The Author(s)

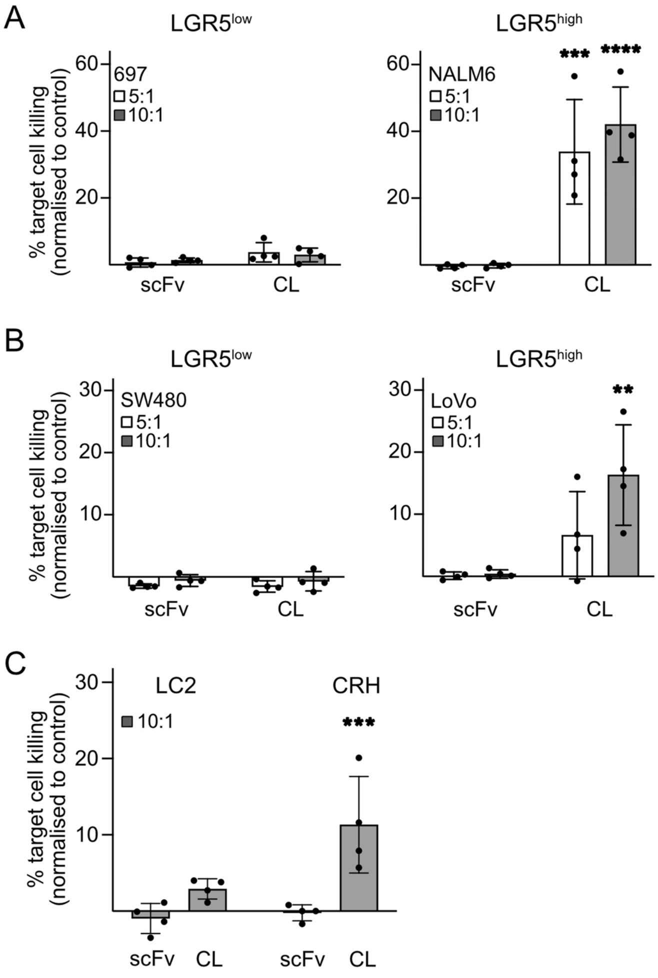

Figure EV3. Differential sensitivity of pre-B-ALL and CRC cell lines as well as B-ALL patient samples to treatment with PBMCs and CL-BiTE.

(**A**) $LGR5^{low}$ and $LGR5^{high}$ expressing human pre-B-ALL cell lines were incubated in the presence of cytotoxic $CD8^{+}$ T cells with either scFv control or CL-BiTEs. Killing was assessed after 6 h at effector to target cell ratios of 5:1 and 10:1. Data shown is from one experiment using $CD8^{+}$ T cells isolated from four individual healthy donors and is presented as mean expression, +/− SD. (**B**) $LGR5^{low}$ and $LGR5^{high}$ expressing human CRC cell lines were incubated in the presence of cytotoxic $CD8^{+}$ T cells with either scFv control or CL-BiTEs. Killing was assessed after 6 h at effector to target cell ratios of 5:1 and 10:1. Data shown is from one experiment using $CD8^{+}$ T cells isolated from four individual healthy donors and is presented as mean expression, +/− SD. (**C**) $LGR5^{low}$ and $LGR5^{high}$ expressing human pre-B-ALL patient samples were incubated in the presence of cytotoxic $CD8^{+}$ T cells with either scFv control or CL-BiTEs. Killing was assessed after 9 h at an effector to target cell ratio of 10:1. Data shown is from one experiment using $CD8^{+}$ T cells isolated from four individual healthy donors and error bars represent mean +/− SD. Source data are available online for this figure.

 © The Author(s)

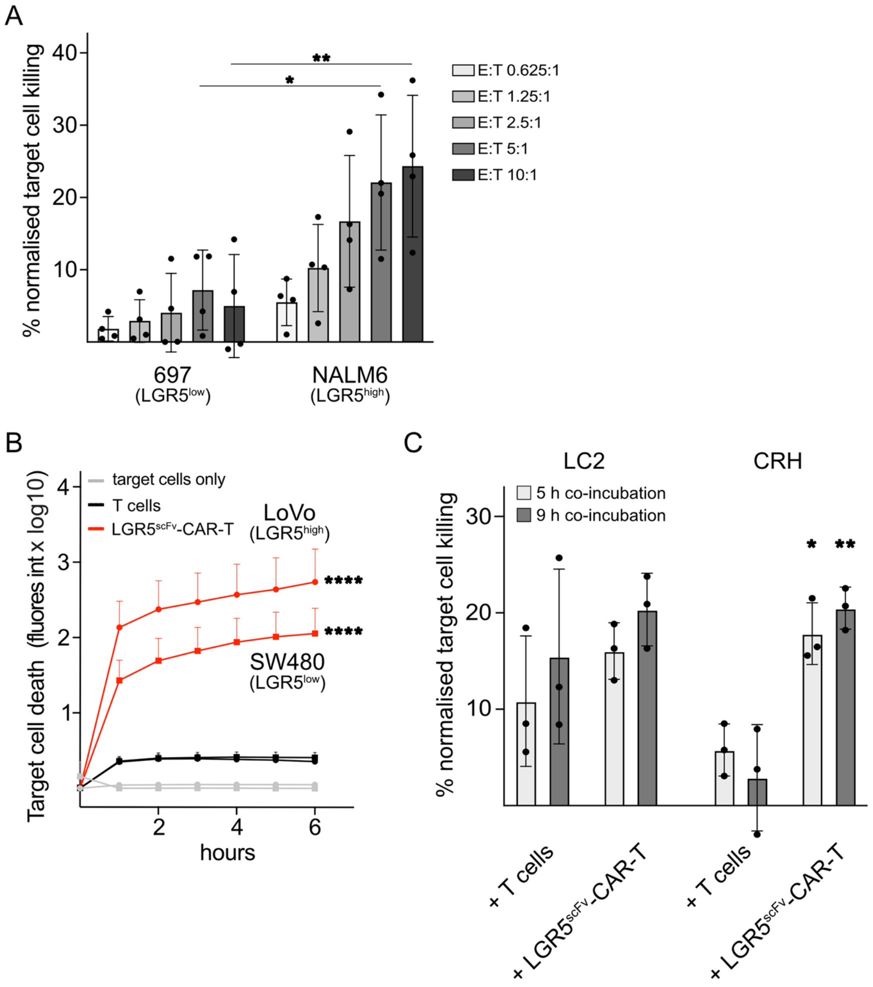

Figure EV4. Differential sensitivity of pre-B-ALL and CRC cell lines as well as pre-B-ALL patient samples to treatment with LGR5^{scFV}-CAR-T cells.

(**A**) Increased killing of LGR5^{high} preB-ALL NALM6 cells relative to LGR5^{low} 697 cells by LGR5^{scFV}-CAR-T cells. Data shown is from two experiments using LGR5^{scFV}-CAR-T cells generated from a total of four independent healthy donors and is presented as mean expression, +/− SD. (**B**) Reduced sensitivity to LGR5^{scFV}-CAR-T-cell killing of LGR5^{low} CRC SW480 cells relative to LGR5^{high} LoVo cells. Data shown is from two experiments using LGR5^{scFV}-CAR-T cells generated from a total of three independent healthy donors and is presented as mean expression, +/− SEM. (**C**) The LGR5^{high} CRH patient-derived pre-B-ALL cell model is more sensitive to killing by LGR5^{scFV}-CAR-T cells compared to LGR5^{low} pre-B-ALL LC2 patient cells. Data shown is from one experiment using LGR5^{scFV}-CAR-T cells generated from a total of three independent healthy donors and is presented as mean expression, +/− SD. Source data are available online for this figure.

© The Author(s)
